# Transcriptomic signature, bioactivity and safety of a non-hepatotoxic analgesic generating AM404 in the midbrain PAG region

**DOI:** 10.1038/s41598-024-61791-z

**Published:** 2024-05-15

**Authors:** Hernan A. Bazan, Surjyadipta Bhattacharjee, Madigan M. Reid, Bokkyoo Jun, Connor Polk, Madeleine Strain, Linsey A. St Pierre, Neehar Desai, Patrick W. Daly, Jessica A. Cucinello-Ragland, Scott Edwards, Javier Recio, Julio Alvarez-Builla, James J. Cai, Nicolas G. Bazan

**Affiliations:** 1 Section of Vascular/Endovascular Surgery, Department of Surgery, Ochsner Clinic, New Orleans, LA 70118 USA; 2 Neuroscience Center of Excellence, School of Medicine, Louisiana State University Health New Orleans, New Orleans, LA 70112 USA; 3 Department of Physiology, School of Medicine, Louisiana State University Health New Orleans, New Orleans, LA 70112 USA; 4 Department of Organic Chemistry and IQAR, University of Alcala, 28805 Alcala de Henares, Madrid Spain; 5 Department of Electrical and Computer Engineering, Texas A&M University, College Station, TX 77843 USA; 6 Department of Veterinary Integrative Biosciences, Texas A&M University, College Station, TX 77843 USA

**Keywords:** Non-opioid, CNS, Liver toxicity, scRNA-seq, Phase 1 trial, Drug discovery, Drug discovery and development

## Abstract

Safe and effective pain management is a critical healthcare and societal need. The potential for acute liver injury from paracetamol (ApAP) overdose; nephrotoxicity and gastrointestinal damage from chronic non-steroidal anti-inflammatory drug (NSAID) use; and opioids’ addiction are unresolved challenges. We developed SRP-001, a non-opioid and non-hepatotoxic small molecule that, unlike ApAP, does not produce the hepatotoxic metabolite N-acetyl-p-benzoquinone-imine (NAPQI) and preserves hepatic tight junction integrity at high doses. CD-1 mice exposed to SRP-001 showed no mortality, unlike a 70% mortality observed with increasing equimolar doses of ApAP within 72 h. SRP-001 and ApAP have comparable antinociceptive effects, including the complete Freund’s adjuvant-induced inflammatory von Frey model. Both induce analgesia via N-arachidonoylphenolamine (AM404) formation in the midbrain periaqueductal grey (PAG) nociception region, with SRP-001 generating higher amounts of AM404 than ApAP. Single-cell transcriptomics of PAG uncovered that SRP-001 and ApAP also share modulation of pain-related gene expression and cell signaling pathways/networks, including endocannabinoid signaling, genes pertaining to mechanical nociception, and fatty acid amide hydrolase (FAAH). Both regulate the expression of key genes encoding FAAH, 2-arachidonoylglycerol (2-AG), cannabinoid receptor 1 (CNR1), CNR2, transient receptor potential vanilloid type 4 (TRPV4), and voltage-gated Ca^2+^ channel. Phase 1 trial (NCT05484414) (02/08/2022) demonstrates SRP-001’s safety, tolerability, and favorable pharmacokinetics, including a half-life from 4.9 to 9.8 h. Given its non-hepatotoxicity and clinically validated analgesic mechanisms, SRP-001 offers a promising alternative to ApAP, NSAIDs, and opioids for safer pain treatment.

## Introduction

Worldwide, pain affects 27% of adults^1^, and given its high prevalence and disability sequelae, it is a global health burden. In the United States (U.S.), pain affects more adults than diabetes and cancer combined, with an estimated cost of $635 billion/year to the healthcare system^2^. Current medications are either addictive (e.g. opioids) or cause harm to the liver (e.g. acetaminophen/paracetamol or ApAP) or kidney (e.g. non-steroidal anti-inflammatory drugs; NSAIDs). Although ApAP is an effective pain reliever in various acute and chronic pain conditions^3–10^, its narrow therapeutic index due to the risk of hepatotoxicity limits its clinical utility. While ApAP is commonly used for mild to moderate pain relief, it is not generally considered as effective as opioids for treating severe pain conditions. Additionally, it's important to note that the efficacy of ApAP in managing chronic pain is limited, and its use is often constrained by its hepatotoxicity. ApAP hepatotoxicity remains the most common cause of acute liver failure in the U.S.^11^ and the United Kingdom (U.K.)^12^. Annually,  ~ 30,000 patients are hospitalized for ApAP hepatotoxicity in the U.S.^13^, and inadvertent hepatotoxicity is the etiology in half of the cases^14^. Although most patients experience only mild acute liver injury, such as a transient increase in liver transaminase release resulting in hepatitis and cholestasis, acute liver failure ensues in untreated patients who have ingested large doses. Some acute fulminant hepatic failure patients progress to convulsions, coma, and death. Though the proportion of liver transplants due to ApAP overdose varies depending on the country, it is generally acknowledged that ApAP overdose is a significant cause of acute fulminant hepatic failure leading to liver transplantation^15^. Notably, up to 20% of liver transplants in some centers are due to ApAP-associated liver failure^16^.

In response, some countries have implemented restrictions on the sale and availability of ApAP to reduce the risk of accidental and intentional overdoses. Australian regulators recently considered an outright ban on ApAP due to these concerns. However, they ultimately elected to implement restrictions on the maximum number of tablets per package and encourage retailers to limit the number of packages consumers can purchase^17^. In 1998, the U.K. introduced regulations to limit the sale of ApAP in non-pharmacy retail outlets to packs containing a maximum of 16 tablets^18^. In Canada, the risks of ApAP toxicity persisted despite labeling changes implemented in 2009 and 2016 to communicate the risks of ApAP overdose and promote safe use; monthly rates of hospital and intensive care unit admissions for accidental ApAP overdose were unchanged from April 2004 and March 2020^19^.

Because of ApAP’s hepatotoxicity, considerable efforts have been devoted to designing safer analgesic and antipyretic analogs^20–23^, including a recent triazole bioisostere^24^. However, none have proven effective beyond the pre-clinical phase. ApAP hepatotoxicity is associated with forming the electrophilic metabolite, *N*-acetyl-*p*-benzoquinoneimine (NAPQI)^25,26^, through an oxidative process mediated by CYP2E1 and CYP3A isoforms of CYP450. NAPQI is normally neutralized by a glutathione (GSH)-mediated Phase II metabolic pathway and eliminated as a mercapturic acid (Supplementary Fig. 1, Path A). During overuse, the conjugative Phase II metabolism becomes saturated, leading to GSH depletion and accumulation of NAPQI that react with nucleophilic macromolecules, triggering events that result in hepatotoxicity and hepatocellular death (Supplementary Fig. 1, Path B), referred to as acetaminophen-induced liver injury (AILI)^27^. To overcome the limitations of ApAP, we describe the synthesis of a novel non-hepatotoxic analog, SRP-001. We present a comprehensive evaluation that includes in vitro and in vivo assessments of hepatotoxicity, evaluations of antinociception and antipyretic efficacy, single-cell transcriptomics to understand the mechanism of action, and safety and pharmacokinetics from a Phase 1 clinical trial.

## Results

### Synthesis rationale of a non-hepatotoxic ApAP analog, SRP-001

**Figure 1:**
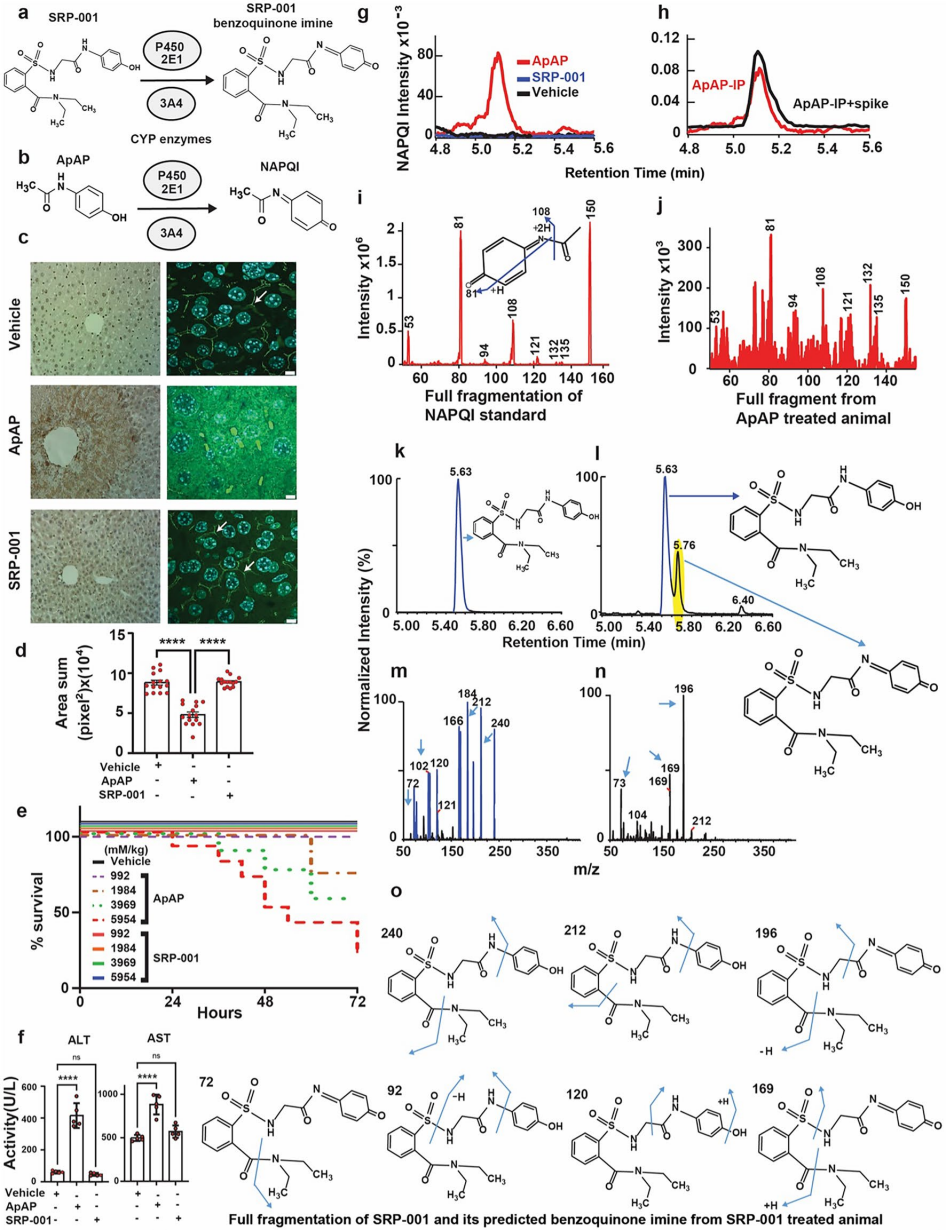
SRP-001’s absent hepatotoxicity is due to lack of NAPQI formation and maintenance of hepatic tight junction integrity. (**a**) Possible oxidation of SRP-001 to the corresponding N-acyl-p-benzoquinone imine by CYP enzymes (CYP P450 2E1 and 3A4). (**b**) ApAP is metabolized by oxidation into NAPQI by CYP P450 2E1 and 3A4. (**c**) Doses known to be hepatotoxic for ApAP (600 mg/kg) but not SRP-001 (600 mg/kg) demonstrates centrilobular hepatic necrosis in nitrotyrosine-labeled hepatic sections from CD1 mice (100x, (**c**) first column), [(*n* = *5*) mice per treatment group]. Hepatic tight junctions (ZO-1 label [3D-stacking, 1000x; scale bar = 5 µm] (**c**) second column) present between hepatocytes reveal a clear “chicken wire” configuration (vehicle) and are disrupted with toxic doses of ApAP but not SRP-001. (**d**) Quantification of the hepatic tight junctions by ZO-1 staining. There is a marked decrease in ZO-1 in ApAP-treated liver sections compared to vehicle and SRP-001, wherein ZO-1 staining is preserved. (**e**) Kaplan–Meier survival curves comparing equimolar doses of ApAP (150 to 900 mg/kg) and SRP-001 (402 to 2414 mg/kg) demonstrate a dose-dependent increase in mortality by 72 h for ApAP-treated groups (1/10 at 1984 mM/kg, 3/10 at 3969 mM/kg, and 7/10 at 5954 mM/kg), with no mortality observed in the SRP-001 and placebo cohorts; n = 10 per group (CD-1 male mice). (**f**) ApAP exposure but not SRP-001 led to an increase in the liver transaminases and 75% (15 out of 20) of ApAP treated animals were dead in 48 h and none in SRP-001 treated animals (data not shown). (**g**) ApAP-treated (red) but not SRP-001-treated (blue) mice produce the hepatotoxic metabolite NAPQI. Serum NAPQI peak is shown at 5.1 min on the chromatogram. NAPQI peak appears after IP-injection with ApAP but not SRP-001 (600 mg/kg). (**h**) ApAP-IP sample (red) with a standard NAPQI spike (black) demonstrates the same chromatographic retention time. (**i**) Full fragmentation pattern of the NAPQI standard and its likely fragments. (**j**) The full fragmentation spectrum from IP-ApAP sample’s NAPQI peak. Note: The spectrum matches well the standard in (**i**). (**k**–**n**) LC–MS/MS retention time peak at 5.63 demonstrates SRP-001 (**k**) as it matches the full fragmentation pattern of SRP-001 from an *ip*-injected SRP-001 animal showing its fragmentation peaks (m); similarly, retention time 5.76 demonstrates the benzoquinoimine produced by SRP-001 (**l**); and (**n**) the resulting fragmentation pattern of the N-acyl-p-benzoquinone imine of SRP-001. (**o**) Structures matching full fragmentation pattern peaks of SRP-001 and its predicted benzoquinoimine.

To address ApAP’s narrow therapeutic index and the clinical need for safer non-opioid pain relievers, we explored ApAP analogs without hepatotoxicity^28^. Our goal was to circumvent toxicity by developing ApAP analogs that connect a saccharin moiety to ApAP's methyl group. To achieve this, we employed an effective synthesis method that involved opening the ring of the heterocyclic moiety, leading to moderately lipophilic compounds, with the R1 and R2 groups being adjustable to influence lipophilicity. SRP-001, detailed in Fig. 1a and the Experimental section of Methods, was selected as the lead clinical candidate due to the absence of in vitro hepatotoxicity and effective in vivo antinociception; the lack of in vitro hepatotoxicity was further validated in vivo.

### SRP-001 is devoid of hepatotoxicity due to lack of NAPQI formation and protection of hepatic tight junctions integrity

ApAP undergoes oxidation via CYP450 enzymes, specifically CYP 2E1 and 3A4, to generate NAPQI^28–31^ as illustrated in Fig. 1b, Supplementary Fig. 1. In experiments involving CD-1 mice, hepatotoxicity is evident following exposure to known toxic dosages of ApAP (600 mg/kg). Conversely, mice administered with SRP-001 at an equivalent dosage (600 mg/kg) exhibit no signs of hepatotoxicity^29,32–35^.

Liver sections of ApAP-treated mice displayed centrilobular necrosis and brown nitrotyrosine/diaminobenzidine-positive staining, while liver sections from mice treated with SRP-001 lack nitrotyrosine staining (Fig. 1c). Moreover, liver sections from SRP-001 treated mice (600 mg/kg) maintained “chicken wire” tight junctions between hepatocytes, as evidenced by Zonula Occludens (ZO-1) staining (Fig. 1c,d), known to be disrupted by ApAP-hepatotoxicity^36^. At a dosage recognized as toxic for ApAP (600 mg/kg), a significant reduction in ZO-1-stained tight junctions is observed, as quantified by the sum of area in pixels^2. In contrast, animals administered either the vehicle or SRP-001 (600 mg/kg) retain the integrity of the hepatic 'chicken wire' tight junction structure. Concurrently, in vivo assessments reveal elevated levels of liver injury biomarkers alanine aminotransferase (ALT) and aspartate aminotransferase (AST) in mice exposed to toxic dosages of ApAP (600 mg/kg), a phenomenon not observed in SRP-001-treated mice (Fig. 1f). Histological analysis further corroborates these findings, showing a marked presence of TUNEL-positive apoptotic nuclei in liver sections from ApAP-treated animals (600 mg/kg), unlike those from SRP-001-treated mice (600 mg/kg) (Supplementary Fig. 2b,c).

Next, we conducted an equimolar dose–response toxicity analysis to compare the toxicity profiles leading to mortality of ApAP and SRP-001. This analysis was critical for robustly comparing their toxic effects, with a particular focus on mortality at equivalent molar concentrations. Over 72 h, with assessments every 6 h, we evaluated the mortality associated with increasing equimolar doses of ApAP and SRP-001 in CD-1 male mice (n = 10 per group). The study utilized the established hepatotoxic threshold of ApAP at 600 mg/kg, as identified in our previous work^28^ and corroborated by other studies^9,29,37^, setting a benchmark for a dose–response curve ranging from 150, 300, 600, to 900 mg/kg of ApAP. Converting these doses to molar concentrations based on the molecular weights of ApAP (151.16) and SRP-001 (405.47), we obtained molar concentrations of 992, 1984, 3969, and 5954 mM/kg for ApAP, corresponding to SRP-001 concentrations of 402, 804, 1609, and 2414 mg/kg, respectively. Notably, the placebo and SRP-001 groups showed no mortality across all dosage levels. In contrast, the ApAP groups exhibited a dose-dependent increase in mortality, with 1/10 mice succumbing at 1984 mM/kg, 3/10 at 3969 mM/kg, and 7/10 at 5954 mM/kg by the end of the study period (Fig. 1e). For the dosing group of ApAP (1984 mM/kg) 1 mouse was found dead around the 60 h time point, for the ApAP (3969 mM/kg) group, 3 mice were dead at time points – 36 h, 48 h, and 60 h, and for the ApAP (5954 mM/kg) group, there was a total of 7 mice found dead; out of which 1 mouse was found dead at the respective time points – 24 h, 36 h, 42 h, 54 h, and 72 h, and 2 mice found dead at the 48 h time point.

We utilized the Cox proportional hazards regression model to evaluate the impact of increasing equimolar doses of ApAP compared to SRP-001 on mortality in CD-1 male mice. We excluded data from the placebo and lowest dose groups of both ApAP and SRP-001 due to the absence of mortality in these groups. Our primary focus was on the higher dosage levels where differences in mortality were evident. The analysis proceeded in two distinct phases: (1) Group Comparison: Initially, we compared mortality rates between the ApAP and SRP-001 groups across all doses. This comparison, conducted using the likelihood ratio test (LRT), revealed a significant disparity in mortality rates between these groups (χ^2^(3) = 19.2, P = 0.0002). Group comparisons via LRT at specific dosages showed significantly higher mortality rates in the ApAP group at 3969 and 5954 mM (P = 0.0315 and P = 0.0003, respectively). In comparison, mortality did not significantly differ between groups at the 1984 mM dose (p = 0.2369). (2) Dose–response analysis within ApAP: Subsequently, we examined mortality differences within the ApAP group at 3969 mM and 5954 mM dosages versus 1984 mM. This analysis highlighted a dose–response relationship, indicating that higher doses of ApAP were linked to increased mortality (χ^2^(2) = 8.4, P = 0.0151). However, post-hoc tests of individual dosages revealed that mortality is significantly increased only at the 5954 mM dose compared to 1984 mM (P = 0.0044).

Additionally, we used Kaplan–Meier analysis (Fig. 1e) to plot survival curves for the two groups across various dosages. This approach allowed for a detailed examination of survival probabilities over time, clearly representing how survival rates varied with different dosages. The log-rank test confirmed a statistically significant difference in survival, with the survival curves supporting the observed dose–response increase in mortality with ApAP. Animals treated with equimolar doses of SRP-001 did not exhibit this trend.

Finally, we uncovered that SRP-001’s absent hepatotoxicity is due to the lack of formation of the toxic quinonimine metabolite NAPQI (Fig. 1g–o and Supplementary Fig. 2a). Serum NAPQI peak is shown at the retention time of 5.1 min on the chromatogram (Fig. 1g). In CD-1 mice injected with a toxic dose (600 mg/kg) of ApAP, a NAPQI peak is seen after *ip*-injection with ApAP but not in the SRP-001. The control, i*p*-ApAP sample with a standard spiked NAPQI, demonstrates the same retention time in the chromatogram (Fig. 1h). LC–MS/MS full fragmentation spectrum from *ip*-ApAP sample’s NAPQI peak is depicted in (Fig. 1i). This was further confirmed with full fragmentation pattern of NAPQI standard. The spectrum matches the standard as the retention time peaks at 5.1 min on the chromatogram (Fig. 1j–l. We also identified the predicted SRP-001 benzoquinoimine from SRP-001-treated animals and full fragmentation pattern of SRP-001 in positive ionization mode in LC–MS/MS. The retention peaks at 5.76 min on the chromatogram indicates the predicted benzoquinoimine of SRP-001 (Fig. 1k–o).

### In vivo antinociception and antipyresis are comparable for ApAP and SRP-001

**Figure 2:**
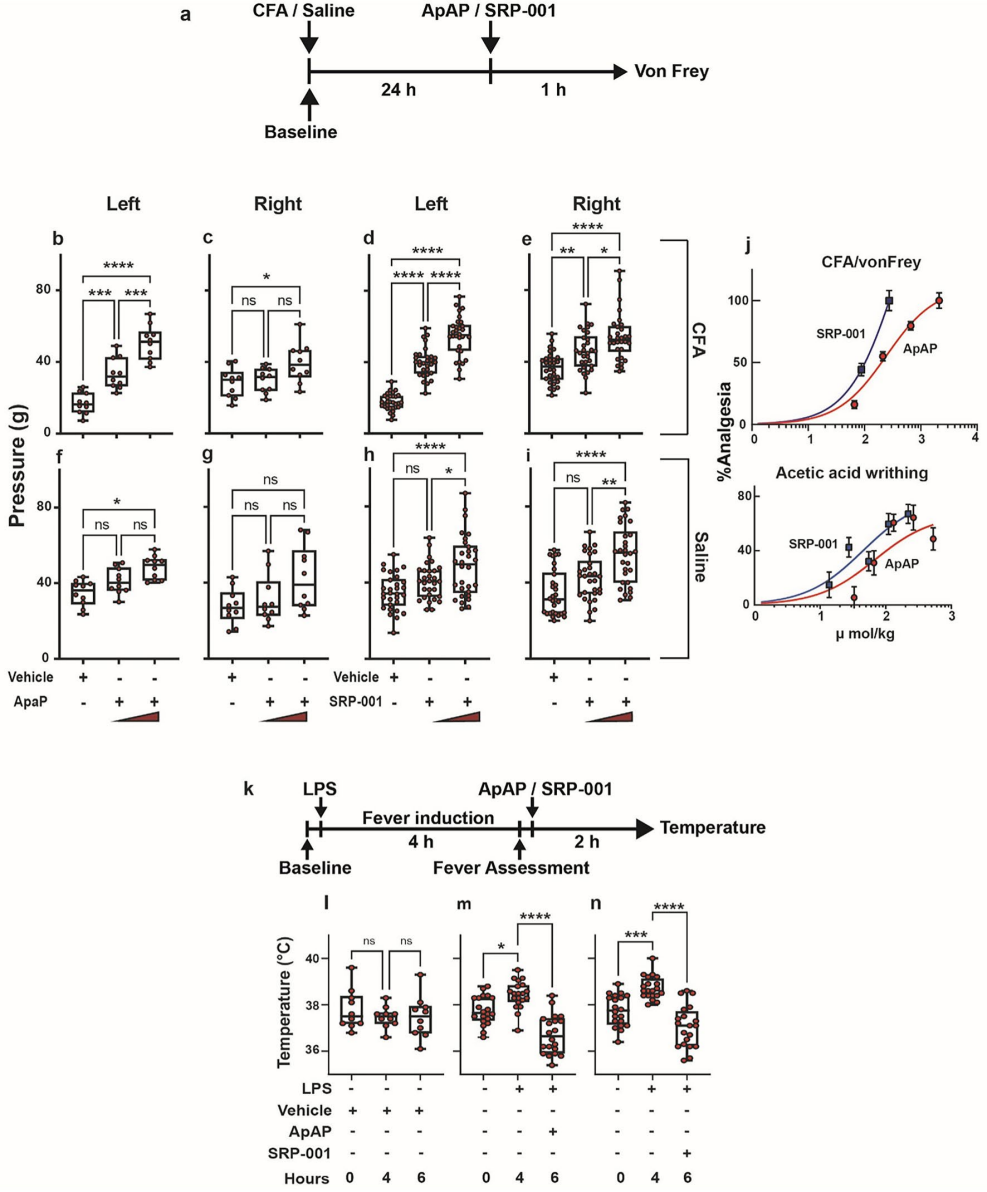
Both ApAP and SRP-001 induce analgesia in the von Frey hyperalgesia in vivo assay and are antipyretic. (**a**) Timeline outlining the experimental design for the von Frey with electronic detection hyperalgesia/allodynia in vivo assay. (**b**–**i**) Two separate doses of ApAP and SRP-001-32 and 100 mg/kg body weight—were tested using young male rats in von Frey. The threshold for paw withdrawal increased from 18 to 40 g and subsequently to 55 g in SRP-001-treated animals; it is more efficacious than ApAP at similar doses. (*n* = *10*) rats for ApAP, and (*n* = *20*) rats for SRP-001. Note: The left hind paw is treated with either CFA or saline, and the right hind paw is an internal control and is not injected. (**j**) SRP-001 has a more potent antinociceptive effect compared to ApAP at equimolar doses (µmol/kg). ED_50_ curves for SRP-001 are shifted to the left compared to ApAP in the hyperalgesia/allodynia (von Frey) and in a visceral pain model (acetic acid writing assay); see Supplementary Figs. 3 and 4 for detailed cohort data and Supplementary Fig. 5 for equimolar ED_50_ curves for each antinociceptive assay. (**k**) Timeline showing experimental design for antipyresis using LPS from *Escherichia coli* (100 μg/kg, 0111:B4). (**l**) there are no significant changes in body temperature of mice injected with 0.9% saline (vehicle) throughout the course of the experiment (n = 10) (**m**,**n**) SRP-001 and ApAP have comparable antipyresis. (n = 20) mice.

We measured in vivo antinociception in mice (CD1 and C57BL/6) and rats (Sprague Dawley) using complementary in vivo animal models of pain and nociceptive sensitivity. These included the complete Freund’s adjuvant (CFA) induced inflammatory pain/von Frey model^38–41^ (Fig. 2, Supplementary Fig. 3), tail flick somatic, and abdominal writhing visceral assays (Supplementary Fig. 4), along with electronic von Frey (eVF) and Hargreaves determination of mechanical and thermal sensitivity (Supplementary Figs. 6, 7). the cohort of young male rats, (Fig. 2b-i) the average baseline hind paw withdrawal threshold to the eVF filament was 35 g pressure. After CFA injection, the withdrawal threshold was 18 g for the injected left hind paw (indicating mechanical hyperalgesia), while for the un-injected right hind paw, the withdrawal threshold remained at 35 g pressure (Fig. 2b,c,f,g) for rats treated with ApAP, and (Fig. 2d,e,h,i) for the rats treated with SRP-001. SRP-001 and ApAP showed comparable antinociceptive activity for this cohort of young female rats (Supplementary Fig. 3a,c). In SRP-001-treated animals, for the CFA-injected paw, the threshold for paw withdrawal increased from 18 to 40 g and subsequently to 55 g (32 mg/kg and 100 mg/kg, respectively) for dose. In ApAP-treated animals, for the CFA-injected paw, the threshold for paw withdrawal increased from 18 to 34 g and subsequently to 50 g (32 mg/kg and 100 mg/kg) for dose, respectively. SRP-001 and ApAP showed comparable antinociceptive activity in a cohort of young and aged animals (Supplementary Fig. 3a–k). Equimolar (µmol/kg) ED_50_ curves were generated using non-linear regression analysis, showing that SRP-001 shifted curves to the left as compared to ApAP (Fig. 2j and Supplementary Fig. 5).

Next, we assessed SRP-001’s antinociception in two other pain models, the tail flick and acetic acid writhing assays in mice. We observed antinociceptive effects using the tail-flick somatic pain assay (Supplementary Fig. 4a–d), noting an increased tail withdrawal time (latency) in SRP-001 or ApAP-treated (32 and 100 mg/kg) CD1 (young male and female) or C57BL/6 (aged male) mice to cold stimulation of the tail tip. For the cold stimulation test, half of the tail was immersed in ice-cold water maintained at 4 °C. The acetic acid-induced abdominal writhing assay, a model of visceral pain in CD1 and C57BL/6 mice in which contraction of the abdominal muscles and stretching of the hind limbs is induced in response to *ip* injection of 0.4% acetic acid (Supplementary Fig. 4e–k). SRP-001 or ApAP (32 and 100 mg/kg)-treated CD1 (young male and female) or C57BL/6 (aged male) mice, but not vehicle, also experienced antinociception. Notably, all three antinociception models demonstrate comparable or improved ED_50_ for SRP-001 compared to ApAP at equimolar (µmol/kg) amounts (Supplementary Fig. 5). Antinociception was also confirmed in the Hargreaves assay in young female and male rats (Supplementary Fig. 6 and Supplementary Table 1) and aged male rats (Supplementary Fig. 7). Lastly, SRP-001 induces similar antipyresis to ApAP in the LPS fever induction model (Fig. 2k–n) and the baker’s yeast fever induction model in CD1 mice (Supplementary Fig. 8).

### Antinociception is mediated via N-arachidonoylaminophenol (AM404) production in the CNS periaqueductal gray area

**Figure 3:**
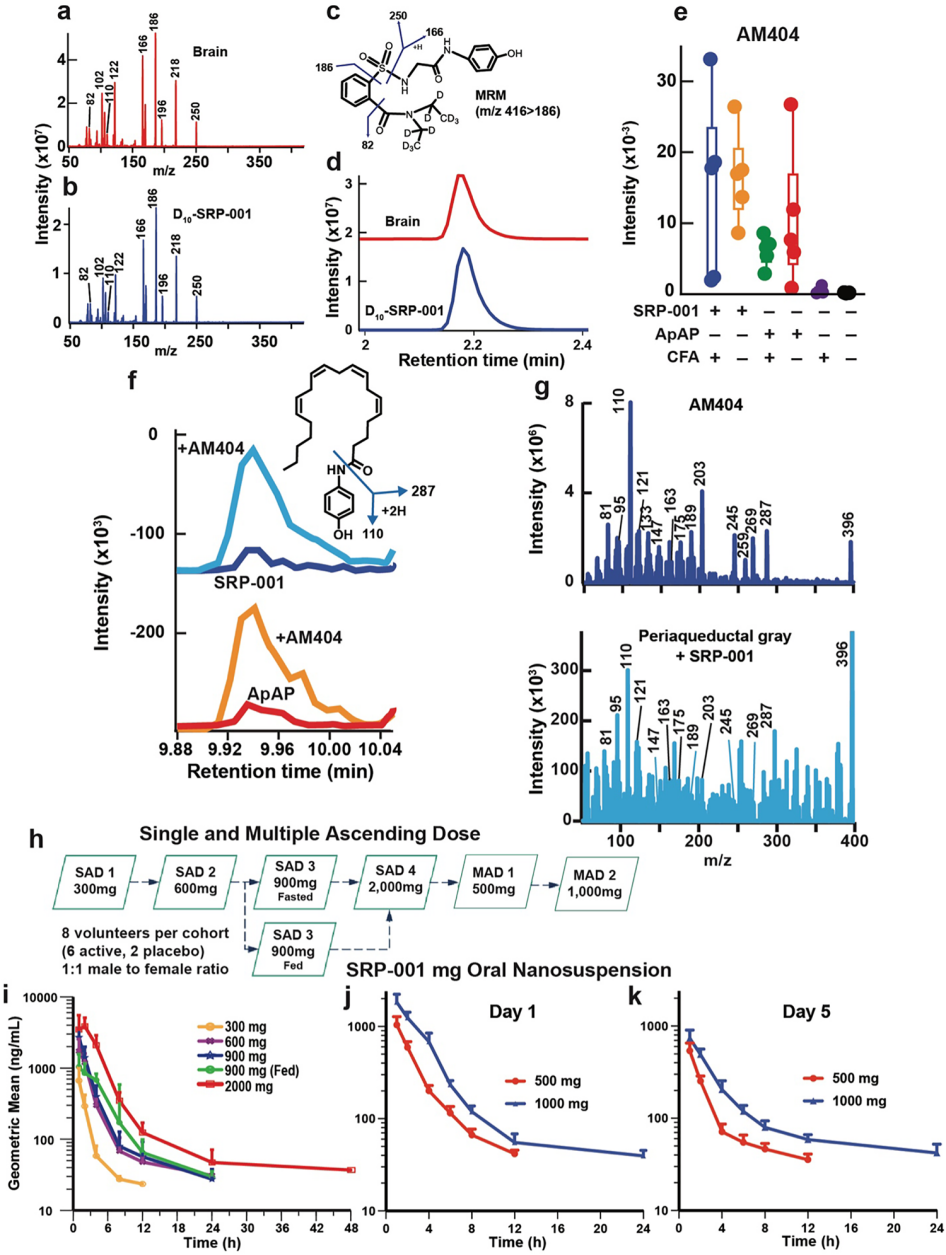
Antinociception/Analgesia is mediated via AM404 production in the periaqueductal gray (PAG) region of the brain and human single and multiple ascending doses (SAD and MAD) Phase 1 clinical trial reveals a favorable pharmacokinetic profile. (**a**) Detection of deuterated (D_10_-SRP-001) in the rat brain 30 min after IP injection. The full fragmentation spectrum of D_10_-SRP-001 detected in the brain (red) matches (**b**) the standard SRP-001 compound spectrum (blue). (**c**) The structure of D_10_-SRP-001 shows fragmentation patterns of D_10_-SRP-001 that match the spectra shown in the brain (red) and with the deuterated standard (blue). (**d**) The MRM (m/z 416 → 186) for D_10_-SRP-001 shows the elution time at ~ 2.2 min. (**e**) Quantification of AM404 production in the CNS periaqueductal gray region. (**f**) AM404 is expressed in the periaqueductal gray area of the brain following SRP-001 IP injection. LC–MS/MS detection of AM404 in the rat periaqueductal gray area after SRP-001 (top panel) and ApAP (bottom panel) IP injections. The AM404 structure and major fragments. (**g**) The peaks are confirmed to be AM404 by co-spiking AM404 standard to the samples (red and orange, respectively). The major peaks detected by LC–MS/MS demonstrate that the peak 9.9 min from LC is the AM404 compound. These peaks align to the (**e**) AM404 fragments. (**h**) Design of the first-in-human Phase 1 clinical trial for SRP-001. A randomized, double-blind, placebo-controlled study to assess the safety, tolerability, and pharmacokinetics of single and multiple ascending oral doses of SRP-001 and to characterize the effect of food on the pharmacokinetics of SRP-001 in healthy male and female subjects. Single ascending dose (SAD) escalation from 300 to 2000 mg (fasted state) and 900 mg (fed state). (**i**) and (**j**) Geometric mean SRP-001 plasma concentration–time profiles following oral administration (logarithmic plot) show a proportional or super-proportional increase in Cmax (peak time to concentration at 1 h post-dose) with a mono- or bi-phasic concentration decline in the SAD (**i**) and the MAD cohorts with an arithmetic mean T_1/2_ of 5.57 h and a geometric mean T_1/2_ of 4.92 h.

Next, we sought to define the uptake of SRP-001 in the nociception area of the brain, the midbrain’s periaqueductal (PAG) area^42,43^. Thirty min following *ip*-SRP-001 administration (32 mg/kg), the PAG was harvested and analyzed by LC/MS–MS. D_10_-SRP-001 was used to match the fragmentation pattern while monitoring analytes. The full fragmentation spectrum of D_10_-SRP-001 detected in the brain (red) matches the standard SRP-001 spectrum (blue) (Fig. 3a,b). Further, the structure of D_10_-SRP-001 (Fig. 3c) shows fragmentation patterns of D_10_-SRP-001 that match the spectra shown in the brain (red) and with the deuterated standard (blue) (Fig. 3d).

ApAP induces antinociception in the midbrain’s region via the formation of its metabolite, N-arachidonoylaminophenol (AM404)^44–46^. AM404 acts on transient receptor potential vanilloid-1 (TRPV1)^46,47^ and cannabinoid 1 receptors^48^ in the brain to produce antinociceptive effects. Thus, we quantified PAG AM404 production by LC–MS/MS, post-dosing with ApAP or SRP-001. Notably, the highest AM404 production in the PAG is in animals treated with SRP-001 compared to ApAP (Fig. 3e). Peak levels of AM404 are observed in animals co-treated with CFA and SRP-001, followed by those receiving a combination of vehicle (0.9% saline) and SRP-001. Notably, AM404 production is diminished in the periaqueductal gray region of rats administered CFA and ApAP. Minimal to undetectable levels of AM404 are found in the periaqueductal gray region of rats treated solely with either CFA or vehicle. The AM404 structure and its major fragments (Fig. 3f), wherein AM404 peaks are confirmed by co-spiking AM404 standard to the SRP-001 and ApAP injected samples (blue and orange, respectively). The top panel represents the AM404 standard, sourced from a pure analytical standard of AM404 (10 ng/mL, Cayman Chemical, Ann Arbor, MI). The bottom panel depicts AM404 levels following intraperitoneal (IP) injection of SRP-001 at a concentration of 32 mg/kg, with animals sacrificed 30 min post-injection, consistent with the CFA/von Frey analgesia model. Subsequent to whole-brain extraction, the periaqueductal gray region was specifically isolated for analysis. The major peaks detected by LC–MS/MS demonstrate that the peak 9.9 min on the chromatogram is the AM404 compound; these peaks align with the AM404 fragments (Fig. 3g).

### Phase 1 trial results

A randomized, double-blind, placebo-controlled Phase 1 trial (ClinicalTrials.gov identifier: NCT05484414) (02/08/2022) was conducted to assess the safety, tolerability, food effect, pharmacokinetics (PK), and pharmacodynamics (PD) of single and multiple ascending doses of SRP-001 (Quotient Sciences, Miami FL USA; Fig. 3h - k and Methods). The trial was conducted in accordance with the International Council for Harmonisation of Technical Requirements for Pharmaceuticals for Human Use (ICH), Guideline for GCP: Consolidated Guidance (E6), attained Institutional Review Board approval, and all applicable regulatory requirements for Phase 1, First in Human clinical trials. Safety measurements and plasma samples to determine PK parameters were collected throughout the study for all subjects and reviewed by the Safety Review Committee. PK stopping criteria were established to assure that systemic exposure to SRP-001 was less than or equal to the systemic exposure achieved at the NOAEL doses in the 28-day repeat-dose toxicology studies in the rat and dog. The trial’s Safety Advisory Committee has approved the publication of the Phase 1 results.

SRP-001 was administered as an oral nanosuspension to 56 healthy volunteers across five Single Ascending Dose (SAD) and two Multiple Ascending Dose (MAD) cohorts, with 42 subjects receiving SRP-001 and 14 receiving a placebo. SRP-001 was well tolerated, with no serious adverse events (SAEs) reported, as detailed in Table 1. Comprehensive safety assessments, including vital signs, physical examinations, continuous electrocardiogram monitoring, and a wide range of laboratory tests (encompassing liver and kidney function, clinical chemistries, coagulation profiles, hematology, and urinalysis), revealed no abnormalities, indicating a favorable safety profile.

**Table 1:** Summary of the results of in vitro and in vivo toxicity studies conducted with SRP-001.

Study	Species	Duration	Doses	Key findings									
Toxicology													
Dose-range- finding oral toxicity study^26^	Rat/Sprague Dawley	3 days	500, 1000, 1500, 2000 mg/kg	All animals appeared unremarkable at gross necropsy. Based on the results of this study, doses for the second phase were selected									
Single oral dose toxicity and PK study^27^	Dog/Beagle	Single dose	300 mg/kg	Bioanalytical analysis revealed poor bioavailability was achieved following dosing. Capsule dosing while well tolerated in dogs at 300 mg/kg was not considered suitable for future studies									
Dose-range- finding and 7- day repeat-dose oral toxicity^28^	Dog/Beagle	7 days	30, 100, 250, 500 mg/kg	Loose feces and emesis were observed following most doses however no dose-dependent trend was observed. No test article related effects on body weight or food consumption were observed									
28-day oral toxicity study with 14-day recovery^29^	Rat/Sprague Dawley	28 days	300, 900, 1500 mg/kg/day	Based on the parameters observed, the no-adverse effect level (NOAEL) of SRP-001 when administered once daily for 28 days to Sprague Dawley rats was 1500 mg/kg									
Two-day oral dose toxicity and PK study^21^	Dog/Beagle	2 days (dosed 3 times daily)	100, 900 mg/kg/day	Nearly all animals had emesis following each dose administration. Other clinical signs observed included loose or watery feces and salivation. Nearly all animals appeared normal prior to receiving their next dose. Body weight loss was also observed; however, no animal lost more than 4% of their body weight. Body weight loss correlated with decreased food consumption on Day 3. Bioanalytical analysis of plasma confirmed SRP-001 exposure in all animals. Overall SRP-001 was tolerated when administered 3 times daily for 2 days at 900 mg/kg/day and plasma exposure was observed									
28-day oral toxicity study with 14-day recovery^30^	Dog/Beagle	28 days	0, 100, 300, 900 mg/kg	No SRP-001 related changes were observed in body weights, food consumption, ophthalmology examination, electrocardiology examination, coagulation parameters or urinalysis parameters. Analysis of dogs following a 14-day recovery period, indicated that nearly all SRP-001 related changes observed on Day 29 were reversible. Only microscopic changes in Kupffer cell pigment and erythrophagocytosis and minimal increased erythroid cellularity of the bone marrow were still present in Group 4 animals following the recovery period. Based the clinical signs, microscopic changes and clinical pathology changes the no adverse effect level (NOAEL) for SRP-001 was 330 mg/kg/day when given T.I.D. for 28 days									
Genotoxicity													
AMES^31^	*Salmonella typhimurium* strains TA98, TA100, TA1535, TA1537, and *E. coli WP2 uvrA*	NA	6.67, 10.0, 33.3, 66.7, 100, 333, 667, 1000, 3333 and 5000 µg/plate	No positive mutagenic responses were observed with any of the tester strains in either the presence or absence of S9 activation									
In vitro mammalian chromosomal aberration assay^32^	Chinese Hamster ovary cells	4-h and 20-h	Doses tested ranged from 0.0405 to 405 µg/mL (1 mM)	Neither statistically significant nor dose dependent increases in numerical (polyploid or endoreduplicated cells) aberrations were observed at any dose in treatment groups with or without S9 (p > 0.05; Fisher’s Exact and Cochran-Armitage tests). The induction of numerical aberrations was within the 95% control limit of the historical negative control data. These results indicate SRP-001 was positive for the induction of structural chromosomal aberrations and negative for the induction of numerical chromosomal aberrations in the presence and absence of the exogenous metabolic activation system									
In vivo mammalian micronucleus assay^33^	Peripheral blood reticulocytes	2 days	500, 1000, 2000 mg/kg	Under the conditions of this study, the administration of SRP-001 at doses up to and including a dose of 2000 mg/kg/day was concluded to be negative in the Micronucleus assay									
In vivo mammalian alkaline comet assay^34^	Rat liver and stomach cells	NA	500, 1000, 2000 mg/kg	Under the conditions of this study, the administration of SRP-001 at doses up to and including a dose of 2000 mg/kg/dose did not cause a significant increase in DNA damage in liver and stomach relative to the concurrent vehicle control. Therefore, SRP-001 was concluded to be negative (non-DNA damaging)									

Organ systems evaluated	Species	Route	Doses	Key findings									
Safety pharmacology													
Cardiovascular (hERG assay)	Human embryonic kidney cells	In vitro	0, 0.1, 1, 10, 100 µM	Weak blocker of hERG current at the highest concentration tested (100 µM) blocking by 21.4 ± 3.9%									
Central nervous^23^ system	Rat/Sprague Dawley	Oral	0, 220, 440, 660	No apparent neuropharmacological effects. Decreased body temp in the high dose group									
Respiratory^24^	Rat/Sprague Dawley	Oral	0, 220, 440, 660	No significant findings									
Cardiovascular^25^	Dog/Beagle	Oral	0, 30, 90, 150	No significant findings									

Participant characteristics for single ascending dose (SAD) phase 1 clinical trial pharmacokinetic findings													
Cohort	Regimen	Treatment	N	T_max_ (h)	C_max_ (ng/mL)	AUC_0-last_ (ng.h/mL)	AUC_0-24_ (ng.h/mL)	AUC_0-inf_ (ng.h/mL)	T_1/2_ (h)	Frel C_max_ (%)	Frel AUC_(0–24)_	Frel AUC_(0- last)_	Frel AUC_(0-inf)_
											(%)	(%)	(%)
1	A	SRP-001 Oral suspension, 100 mg/mL, or matching placebo; Fasted	6	1 (1.00–1.00)	665 (55.5)	1320 (53.4)	1620 (37.8)	1630 (37.4)	2.38 (65.9)	NA	NA	NA	NA
2	B	SRP-001 Oral suspension, 100 mg/mL, or matching placebo; Fasted	6	1 (1.00–2.00)	1740 (48.7)	4940 (38.3)	5660 (25.3)	5840 (29.5)	5.04 (107.8)	NA	NA	NA	NA
3	Period 1: C	SRP-001 Oral suspension, 100 mg/mL, or matching placebo; Fasted	6	1 (1.00–1.00)	2700 (35.1)	6810 (40.0)	6870 (39.3)	6970 (39.5)	3.26 (62.1)	NA	NA	NA	NA
	Period 2: D	SRP-001 Oral suspension, 100 mg/mL, or matching placebo; Fed	6	1 (1.00–8.00)	1200 (32.4)	6130 (25.4)	6250 (22.3)	5910 (17.1)	3.79 (74.5)	NA	NA	NA	NA
4	E	SRP-001 Oral suspension, 100 mg/mL, or matching placebo; Fasted	6	1 (1.00–2.00)	4590 (34)	19,100 (26.2)	18,900 (25.7)	19,600 (25.7)	5.96 (65.9)	48.4a (21.9)	99.2a (19.0)	98.4a (16.3)	101a (20.3)

Participant characteristics for single ascending dose (SAD) phase 1 clinical trial safety findings													
Parameter	Clinically significant abnormality (N)												
	Cohort 1 (Regimen A)	Cohort 2 (Regimen B)	Cohort 3 (Regimen C)	Cohort 4 (Regimen E)									
	(300 mg SRP-001)	(600 mg SRP-001)	(900 mg SRP-001)	(2000 mg SRP-001)									
	Fasted	Fasted	Fasted	Fasted									
	[n = 6]	[n = 6]	[n = 6]	[n = 6]									
Clinical chemistry	0	0	0	0									
pharHematology	0	0	0	0									
Urinalysis	0	0	0	0									
Vital signs	0	0	0	0									
ECG	0	0	0	0									
Physical exam	0	0	0	0									
Adverse events	0	0	0	0									

Participant characteristics for single and multiple ascending dose cohorts (SAD and MAD) Phase 1 clinical trial pharmacokinetic findings. Participant Characteristics for SAD Phase 1 clinical trial safety findings.

Pharmacokinetic (PK) analysis showed a dose-proportional or super-proportional increase in the geometric mean maximum concentration (C_max_) and area under the curve (AUC_(0-∞)_) values across dosing ranges from 300 to 2000 mg, followed by a mono- or bi-phasic decline in concentrations (Fig. 3i–k). As SRP-001 is increased from 300 to 2000 mg, the maximum and overall plasma exposure of SRP-001 based on geometric mean (geometric CV%) C_max_, AUC_(0-∞)_, and geometric mean half-life (T_1/2_) were: 665 ng/mL, 1630 ng.h/mL and 2.4 h (300 mg fasted); 1740 ng/mL, 5840 ng.h/mL and 5.0 h (600 mg fasted); 2700 ng/mL, 6970 ng.h/mL, and 3.3 h (900 mg fasted); 1200 ng/mL, 5910 ng.h/mL, and 3.8 h (900 mg fed); and 4590 ng/mL, 19,600 ng.h/mL, and 6.0 h (2000 mg fasted), respectively.

In the MAD cohorts, the geometric mean T_1/2_ was 3.06 h on Day 1 and 4.92 h on Day 5 for the 500 mg dose, and 4.05 h and 9.77 h, respectively, for the 1,000 mg dose on Day 1 and Day 5.

### Cell heterogeneity in midbrain PAG area

**Figure 4:**
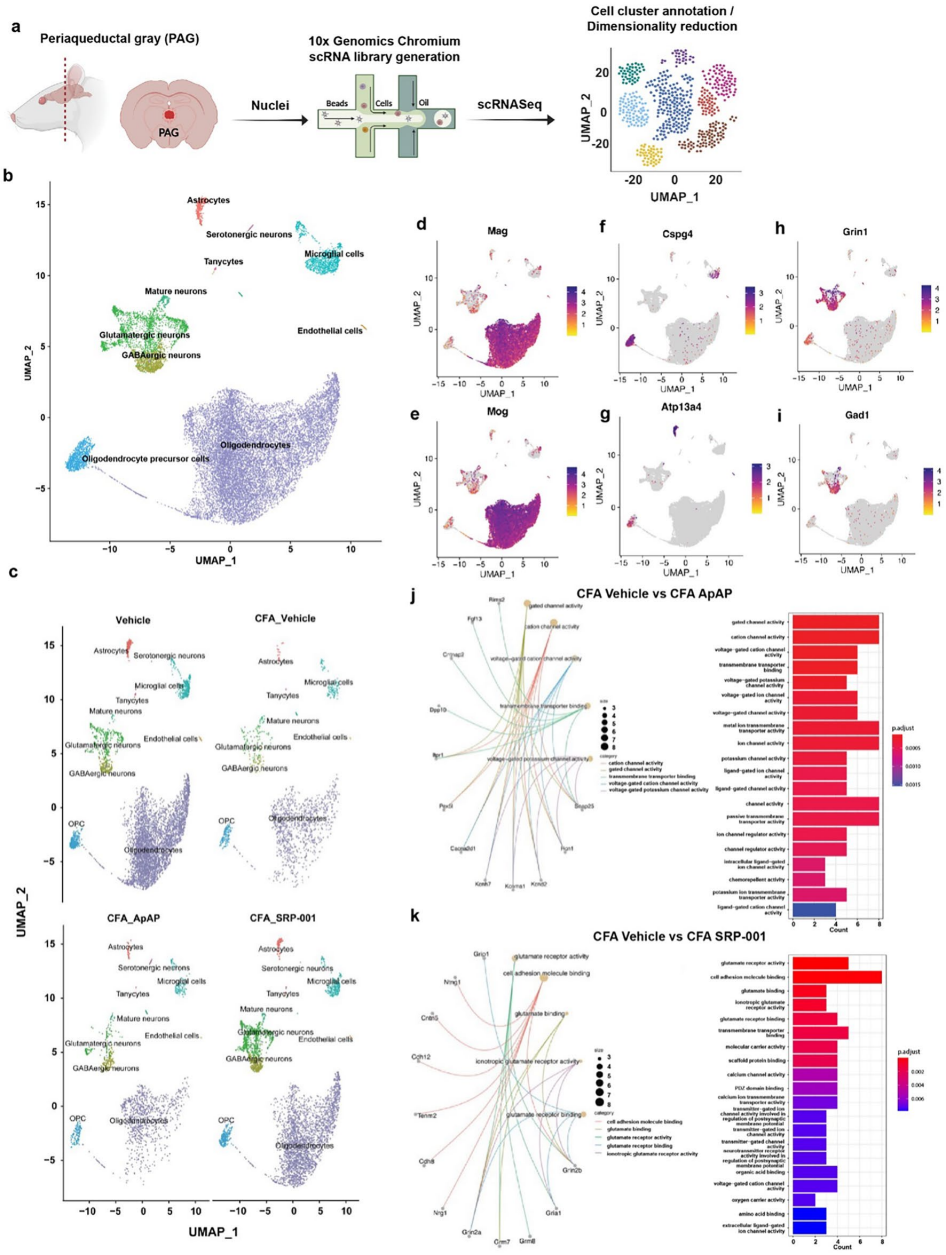
Single-cell transcriptomics of midbrain periaqueductal gray (PAG) cells in CFA-induced chronic inflammatory pain model. (**a**) Overview of single-cell RNA sequencing (scRNA-seq) experimental design to define the mechanism of action (MOA) of ApAP and SRP-001. The periaqueductal gray (PAG) midbrain region was dissected to isolate cell nuclei for sequencing library generation using the 10 × Genomics Chromium platform. Single cell data was embedded after dimensionality reduction using uniform manifold approximation and projection (UMAP). Cell clusters were annotated, followed by differential gene expression analysis. (**b**) UMAP plots showing the distribution of annotated cell clusters color-coded by each cluster – astrocytes, excitatory neurons, inhibitory interneurons, oligodendrocytes, oligodendrocyte precursor cells (OPC), and microglia, from the aggregated clusters of all the 4 samples. (**c**) UMAP plots showing the distribution of annotated cell clusters generated by the workflow of Seurat across 4 different samples – Vehicle, CFA_Veh, CFA_ApAP, and CFA_SRP-001, respectively. (**d**–**i**) Marker gene feature plots showing selected distinctive marker genes used for cell annotation of single cell clusters into 6 different cellular subpopulations. (**d**,**e**) Oligodendrocytes (**f**), Astrocytes (**g**) OPC (**h**) Glutamatergic neurons i GABAergic neurons. (**j–k**) GO enrichment analysis results for top 50 differentially expressed genes between CFA_Vehicle and CFA_ApAP, and between CFA_Vehicle and CFA_SRP-001 – gene concept network showing the linkage between the DE genes and GO terms, and Barplot of the enriched GO terms from the selected genes.

Subsequently, we endeavored to explore the single-cell transcriptome of the eVF rat mid-brain PAG region to further define the mechanisms of action (MOA) of chronic inflammatory pain and, ultimately, SRP-001- and ApAP-mediated pain modulation. Left hind paws of Sprague Dawley rats were injected with 150 µl of 50% complete Freund’s adjuvant (CFA), freshly diluted with sterile saline on the day of administration, to induce conspicuous long-standing inflammatory pain in the rats’ hind paws, along with mechanical hyperalgesia tested using eVF.to induce local inflammation and a chronic pain state. We dosed the rats with ApAP or SRP-001 (100 mg/kg), and after 1 h, they were euthanized to dissect the PAG from flash-frozen brains for 10xGenomics single-nuclei RNA library preparation (Fig. 4a).

### Data pre-processing results

Uniform Manifold Approximation and Projection (UMAP) was used to perform dimensionality reduction, and the embedded cells were arranged according to expression profile similarity in a 2-D UMAP scatter plot to show their distribution. The UMAP plot shows that for each sample – Vehicle, CFA_Vehicle, CFA_ApAP, and CFA_SRP-001 – similar cell clusters were retained and annotated into 10 principal cell types – astrocytes, serotonergic neurons, glutamatergic neurons, GABAergic neurons, mature neurons, tanycytes, endothelial cells, microglia, oligodendrocytes, and oligodendrocyte precursor cells (OPCs) (Fig. 4b,c). Cells were annotated with differentially expressed marker genes using R package scType described in Methods (Fig. 4d–i). Feature plots for the top marker gene in each major cluster were made, and UMAP plots for annotated clusters for each group show differences in cell type distribution and quantity (Fig. 4d–i).

### Gene ontology enrichment analysis

Gene ontology (GO) utilizes the GO knowledgebase to interpret large-scale molecular biology experiments with the endpoint of defining statistically significant similarities or differences between experimental conditions^49^. The knowledgebase provides computational representation of the function of genes and how the genes contribute to a biologic process. Differential expression of genes between Vehicle and CFA_Vehicle treated groups showed enrichment in tau protein binding, molecular carrier activity, peptide binding, GTP binding, and amyloid-beta binding as well as other GO terms (Supplementary Fig. 18a). The linkage between these terms based on similar genes are shown, and the specific gene role, in particular, GO terms was visualized (Supplementary Fig. 18b,c). Differential expression of genes involved in multiple types of gated channel, cation/ion channel, and potassium channel activity was observed from the comparison of CFA_Vehicle and CFA_ApAP (Fig. 4j). The comparison between CFA_Vehicle and CFA_SRP-001 defined differentially expressed genes enriched in GO terms for glutamate receptor activity, glutamate binding, calcium channel activity, and multiple other terms related to ion channel activity like the observed terms from CFA_ApAP (Fig. 4k).

### Principal component analysis

Dimensionality plots from principal component analysis (PCA) show the top 30 differentially expressed genes in principal clusters 1–10 selected based on the elbow plot (Supplementary Fig. 9). Highly variable genes were identified in each group, and the top 10 from each group were visualized (Supplementary Fig. 10).

### Differential gene expression of neurons and other cell clusters of PAG

**Figure 5:**
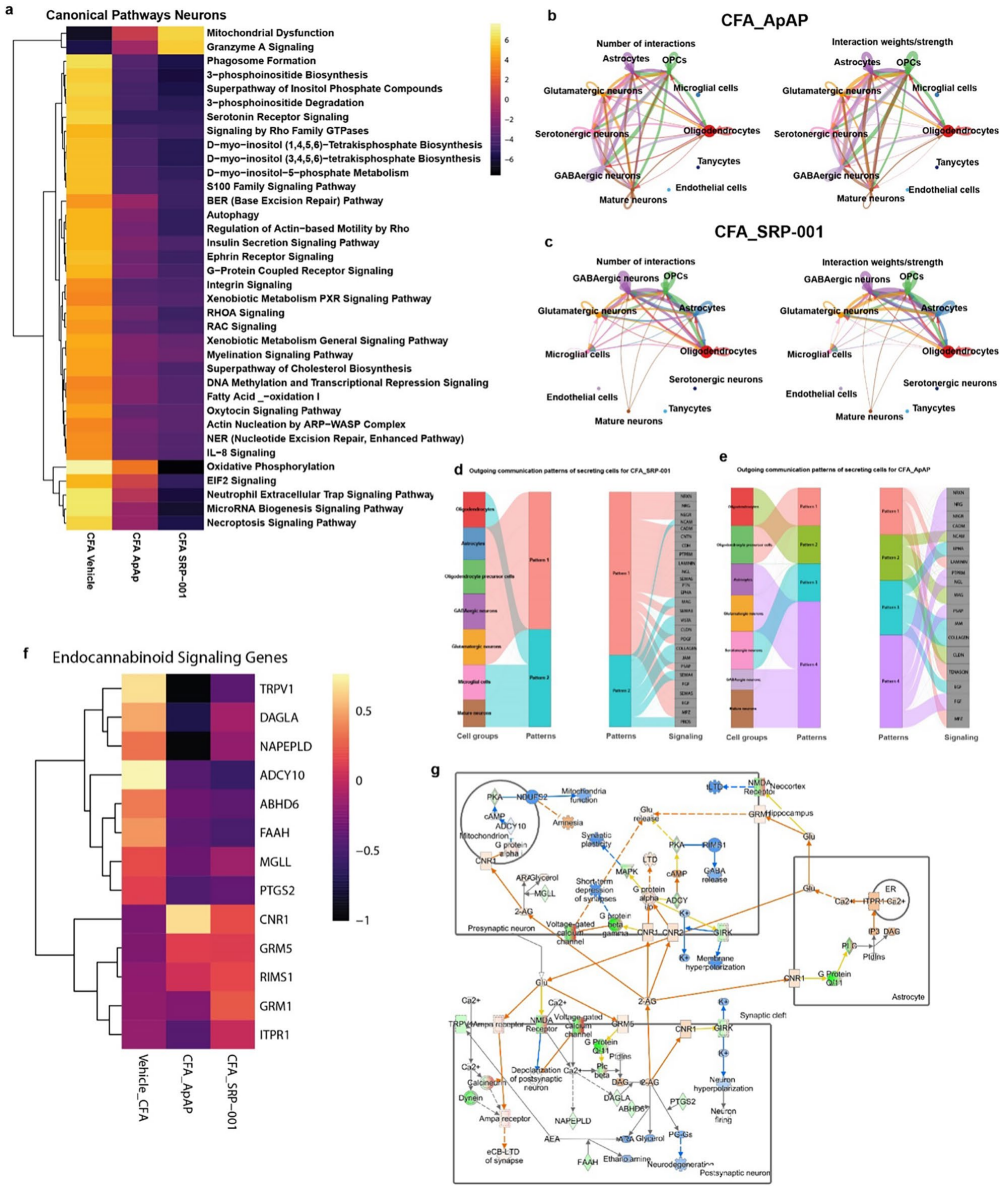
Cell annotation, marker gene feature plots, and endocannabinoid signaling pathway from neurons of PAG region cells in CFA-induced chronic inflammatory pain model with ApAP and SRP-001 treatments. (**a**) Heatmap of top 40 canonical pathways for neuronal clusters across samples – CFA_Veh, CFA_ApAP, and CFA_SRP-001 with the comparisons between them as follows – Vehicle vs CFA_Vehicle, CFA_Vehicle vs CFA_ApAP, and CFA_ApAP vs CFA_SRP-001. The heatmap shows that when CFA_Vehicle is compared to Vehicle, there is upregulation of several genes that are predicted to affect cellular signaling related to these pathways highlighted in the heatmap, and when CFA_ApAP and CFA_SRP-001 are compared to CFA_Vehicle, there is downregulation of those upregulated pathways in both cases, with more downregulation for CFA_SRP-001 than CFA_ApAP. (**b**,**c**) CellChat computed cell–cell interaction network for CFA_ApAP and CFA_SRP-001 group datasets showing number and strength of interactions describing similarities between the two groups. (**d**,**e**) Outgoing communication patterns in individual secreting cell types for CFA_ApAP and CFA_SRP-001. (**f**) Heatmap of DESeq2-normalized expression values from scRNA-seq data for genes involved in endocannabinoid signaling genes. (**g**) Endocannabinoid signaling pathways generated by Qiagen Ingenuity Pathway Analysis (IPA) with an overlay based on DESeq2-normalized expression values of differentially expressed genes (DEGs) in neuronal clusters CFA_Vehicle vs CFA_SRP-001 treatments, respectively.

Differential expression analysis between groups Vehicle vs. CFA_Vehicle, CFA_Vehicle vs. CFA_ApAP, and CFA_Vehicle vs. CFA_SRP-001 was performed using DESeq2, and the top 50 DE genes are shown in heatmaps illustrating that both ApAP and SRP-001 are predicted to regulate similar gene regulatory pathways and that SRP-001 changes the effects based on fold regulation prediction scores (Supplementary Fig. 11). A comparative analysis of the top 40 canonical pathways across neuronal clusters was conducted, generating a heatmap for visual representation of the following sample comparisons: Vehicle vs CFA_Vehicle, CFA_Vehicle vs CFA_ApAP, and CFA_ApAP vs CFA_SRP-001 (Fig. 5a). Pathways depicted in the heatmap were identified using Qiagen's Ingenuity Pathway Analysis (IPA) tool, applied to a normalized matrix of differentially expressed genes between the neuronal clusters under comparison. The heatmap reveals an upregulation of genes impacting cellular signaling pathways when comparing CFA_Vehicle to Vehicle. Conversely, both CFA_ApAP and CFA_SRP-001 exhibit downregulation of these upregulated pathways when compared to CFA_Vehicle, with a more pronounced downregulation observed for CFA_SRP-001. We also investigated a compendium of known and validated pain-related genes^50,51^ and defined the differential expression of these genes across sample comparisons – CFA_Vehicle vs Vehicle, CFA_ApAP vs CFA_Vehicle and CFA_SRP_001 vs CFA_Vehicle and also across different cell clusters between these samples. Dot plots show that several of these genes are differentially expressed across samples and similar among CFA_ApAP and CFA_SRP-001 treatment groups. Genes were separated based on classification as ion channel-, G-protein coupled receptor-, or transcription factor-associated (Supplementary Figs. 12–14).

### Comparing unbiassed signaling pathways and networks between samples to discern the MOA and cellular signaling between SRP-001 and ApAP

Next, CellChat^52^ analysis was employed to computationally model anticipated cell–cell interactions, utilizing single-cell data to elucidate potential mechanistic parallels between the CFA_ApAP and CFA_SRP-001 groups. The cell–cell interaction maps demonstrating number and strength of interactions is shown in Fig. 5b,c. River plots for outgoing communication patterns of secreting cells and the corresponding pathways were determined for CFA_ApAP and CFA_SRP-001 from this analysis as well (Fig. 5d–e). Next, we explored well-defined ApAP-related target networks and signaling pathways: endocannabinoids; mechanical nociception; and fatty acid amide hydrolase (FAAH) using Qiagen’s IPA analysis tool (Fig. 5f–g and Supplementary Figs. 15, 16). These analyses demonstrate that the gene expression profiles for ApAP-targeted pain-related genes in these pathways are activated and inhibited in a similar manner between SRP-001 and ApAP. Heatmaps were generated from exported IPA tables containing the pathways’ analysis values and corresponding gene symbols. This highlights the similarities in gene regulatory activity of endocannabinoid signaling genes TPRV1, ADCY10, ABHD6, FAAH, PTGS2, CNR1, GRM5, RIMS1, GRM1, and ITPR1 between ApAP and SRP-001(Fig. 5f). Genes implicated in mechanical nociception—including TRPV1, NTRK1, ASIC3, TRPA1, TMEM120A, CX3CL1, KCNA1, IL6, GRIN2B, GRM8, PAWR, KCNT1, CASKIN1, and IL18—exhibit comparable modulatory efficacy between SRP-001 and ApAP, as detailed in Supplementary Fig. 16a). Similarly, FAAH signaling genes were more significantly regulated by SRP-001 than ApAP, specifically: FOS, ATP5PD, Rps3a1, RPL17, RPS2, RPL5, RPS18, and TRH (Supplementary Fig. 16b). Moreover, the expression of these pain-related genes is higher in SRP-001 as compared to ApAP. In summary, canonical pathway analysis with predicted inhibition or activation shown in heatmaps highlights the similarities between CFA_ApAP and CFA_SRP-001 in different cell clusters – neurons (Fig. 5a), oligodendrocytes, astrocytes, microglia, and interneurons (Supplementary Fig. 17a–d) respectively.

### Genotoxicity, safety pharmacology, and in vivo non-clinical toxicology

To transition SRP-001 into the clinic for human use, a full set of in vivo and in vitro genotoxicity studies were performed to assess the potential genotoxicity of SRP-001. This included a full battery of International Conference on Harmonization (ICH) compliant studies, including in vitro Ames and mammalian chromosomal aberration assays, in vivo mammalian micronucleus and Comet assays indicating that SRP-001 is not genotoxic. Further, SRP-001 has no effects on pulmonary function or cardio-telemetry and is non-cardiotoxic (Table 1 and Methods).

To determine the appropriate doses, maximum tolerated dose studies (non-GLP) were conducted prior to the definitive GLP systemic toxicity studies. SRP-001 was evaluated by a full battery of GLP-compliant toxicity studies. Systemic toxicity was evaluated in Sprague–Dawley rats and Beagle dogs by oral administration over 28 days (Supplementary Fig. 19 and Supplementary Table 2). Importantly, no treatment-related adverse effects were seen during 28 days of daily oral doses of SRP-001 in rats at doses up to 1500 mg/kg/day or in dogs at doses up to 330 mg/kg/day. Single doses of up to 2000 mg/kg in the rat and 1200 mg/kg in the dog of SRP-001 did not result in any mortality, whereas all ApAP-treated mice expired following a 900 mg/kg dose (Methods).

## Discussion

The use of opioids for pain management is linked to opioid use disorder (OUD) due to their abuse potential^53^. In 2021, the U.S. saw 107,000 drug overdose deaths^54^, with over 80,000 attributed to opioids. Many of these cases originated from prescription opioid misuse for pain management, leading to opioid use disorder (OUD) and even transitioning to heroin use. According to the National Institute on Drug Abuse (NIDA), around 21–29% of patients prescribed opioids for chronic pain misuse them, and about 8–12% develop OUD^55^. Although ApAP is most used to treat short-term pain, hepatotoxicity is a risk from overuse, and it is the most common cause of fulminant hepatic failure in patients with compromised liver function. And NSAID overuse carries risks of GI bleeding and nephrotoxicity. The high levels of opioid addiction, misuse, and overdose and the toxicity profiles of currently available pain medications underscore the need for safe, effective, non-opioid pain medications.

While ApAP is generally deemed safe within prescribed dosages, it is crucial to acknowledge that certain populations—specifically, individuals with liver dysfunction or regular alcohol consumption—encounter a reduced therapeutic-to-toxic dose margin^56–59^. The U.S. FDA has taken steps to mitigate this risk, further underscoring the need for developing safer alternatives ^60,61^. Owing to ApAP’s hepatotoxicity, we characterized SRP-001 from a library^28^ of ApAP analogs that lacked hepatotoxicity and exhibited antinociception and antipyresis. SRP-001 is not hepatotoxic because it does not generate NAPQI or disrupt hepatic tight junctions, which are hallmarks of ApAP hepatotoxicity. An equimolar dose–response toxicity analysis comparing the mortality effects of ApAP and SRP-001 in CD-1 male mice revealed that while the placebo and SRP-001 groups showed no mortality across all dosages, the ApAP groups exhibited a dose-dependent increase in mortality. Employing the Cox proportional hazards regression model, we found significant differences in mortality rates between the ApAP and SRP-001 groups, particularly at higher ApAP concentrations. Kaplan–Meier analysis further supported these findings, illustrating a clear dose–response increase in mortality with ApAP reaching 70% within 72 h, in contrast to SRP-001, which showed no mortality in the treated animals.

For the in vivo antinociception studies, we used reliable, reproducible animal models and assays of acute and chronic pain used in the preclinical testing of novel and translationally relevant analgesics. CFA is a chronic inflammatory pain model, and eVF and Hargreaves are the detection methods to measure mechanical (eVF) and thermal (Hargreaves) nociceptive sensitivity, respectively. We employed a strategic array of pain models to comprehensively evaluate the analgesic efficacy of SRP-001. The von Frey test with electronic detection (eVF) explores its effects in an inflammatory pain setting, highlighting its potential to alleviate mechanical allodynia. The tail flick test assesses its rapid analgesic effects in acute somatic pain scenarios, indicating its ability to modulate thermal pain perception effectively. Lastly, we chose the acetic acid-induced writhing model to determine the SRP-001’s impact on visceral pain. Furthermore, we included both male and female animals, as well as young and aged cohorts, across the behavioral experiments to address potential sex- and age-related variations in pain perception and SRP-001 antinociceptive efficacy. Taken together, these pain models suggest that SRP-001 produces efficacious antinociception across various acute and chronic pain conditions, with a larger therapeutic index than ApAP and broad efficacy. As an extension of the current studies, future preclinical and clinical work should determine the efficacy of SRP-001 in reducing the negative affective dimension of pain^41^ as well as carefully screen for any undesirable side effects.

Despite being available since the 1950s, ApAP’s analgesia MOA remains to be fully discerned. Increasing evidence, however, strongly supports that AM404^44,45^ is fundamental to its analgesia. In the liver, ApAP is converted to p-aminophenol, which, in turn, is converted by FAAH in the brain in the presence of arachidonic acid to make AM404. Current understanding of ApAP’s MOA analgesia is through AM404 via the endogenous cannabinoids through CB1 receptors and activation of the TRPV1 channel-receptor signaling in the midbrain PAG^42,47^. Here, we demonstrate after *ip-*injection of D_10_-SRP-001 in rat brain and also that SRP-001 produces more AM404 than ApAP in the PAG where FAAH/CB1/TRPV1 channel triad co-locates^42^. Beyond this, the central mechanisms for ApAP’s analgesia are still unclear.

By examining FAAH gene expression interaction networks, we found similarities in gene modulation related to pain signaling between ApAP and SRP-001. Downregulation of FAAH, CORO2A, and RPL7L1 measured directly from scRNAseq was observed with both ApAP and SRP-001. FAAH is a key enzyme in pain signaling by modulating endocannabinoid levels and lipid-based signaling mediators that regulate physiological processes, including pain perception. The primary function of FAAH is degradation of anandamide (AEA), a main endocannabinoid involved in pain modulation. The role of FAAH in pain signaling has been extensively studied, and it has been found that inhibition of FAAH activity can lead to an increase in AEA levels, reducing pain perception. This process is mediated by the activation of CB1 and CB2 receptors^62^. In addition to AEA, FAAH also degrades other bioactive fatty acid amides, such as oleamide and palmitoylethanolamide, which possess antinociceptive properties^63^. Directly measured expression from scRNAseq data shows decreased expression of FAAH and genes related to the molecular function of FAAH by SRP-001 and ApAP, indicating that the bioactivity of SRP-001 produces antinociception through FAAH inhibition.

Mechanical nociception gene networks displaying genes that are involved in this biological function also demonstrate the similarities between gene modulatory mechanisms of SRP-001 and ApAP. Activation of vlPAG-located TRPV1 channels, which are expressed on glutamatergic neurons, is required by ApAP to exert analgesic effects via AM404. Activation of these receptors produces analgesic effects through increasing glutamate release to act on mGlu_5_ receptors. ApAP has recently been shown to increase glutamate and GABA levels in the presence of an acute pain stimulus in a FAAH-dependent manner^64^. Ion channels present another key target for pain modulation due to their localization in primary sensory neurons. Various receptors and ion channels play a critical role in determining neural excitability; PAG pain transmission through descending facilitatory effect can potentially contribute to the development and maintenance of hyperalgesia, which is dependent upon neuron network activity. Previously, calcium and sodium gated ion channels were the focus of pain signaling, but recently, potassium channels have emerged as a potential target for novel analgesics^65^. Potassium channels are necessary to sustain resting membrane potential and repolarizing neurons after an action potential; thus, several potassium channels have been implicated in pain modulation in the PAG area, including the ATP-sensitive potassium (K-ATP) channels, the calcium-activated potassium (KCa) channels, and the two-pore domain potassium (K2P) channels^66^. Concerted activation of proteins, including ion channels, can lead to peripheral sensitization, thus, inhibition of ion channel activity can reduce neuronal excitability and prevent this peripheral sensitization. Modulation of mechanical nociceptive genes TRPV4, TRPV1, ASICS3, TRPA1, KCNA1, and KCNT1 by SRP-001, provides an early indication of broad ion channel inhibition and potential MOA.

TRPV1 has emerged as a critical target not only in peripheral but also in central mechanisms of pain management. Ohashi et al.^67^ demonstrated TRPV1's broader implications in pain modulation and how ApAP derivatives can target TRPV1 to alleviate pain. This aligns with earlier pivotal work from Starowicz et al.^68^ that revealed TRPV1's significant role in central pain pathways, specifically within the PAG-Rostral Ventromedial Medulla pathway axis. These studies collectively highlight the dual functionality of TRPV1 in sensing and modulating nociceptive signals, proposing a more integrated approach in pain therapy that targets both peripheral and central aspects of pain regulation. Recognizing the role of TRPV1 within the broader CNS framework offers promising avenues for developing more effective analgesic strategies. Future research must continue to explore TRPV1's extensive roles within both the peripheral and central domains to leverage its therapeutic potential for comprehensive pain management fully.

We demonstrate that SRP-001 offers a favorable safety profile compared to ApAP, attributing its lack of hepatotoxicity to two distinct mechanisms: the absence of NAPQI formation and the maintenance of hepatic tight junction integrity. Unlike ApAP, which undergoes metabolic activation by CYP450 enzymes leading to NAPQI, a highly reactive hepatotoxic metabolite, SRP-001 does not follow this metabolic pathway and thus avoids the consequent hepatic injury. Furthermore, histological analyses have demonstrated that SRP-001 preserves the integrity of hepatic tight junctions, an attribute not observed with high dosages of ApAP, which disrupts these cellular structures and contributes to liver damage. Moreover, as a non-NSAIDs novel small molecule, SRP-001 does not share the nephrotoxicity and GI ulceration risks associated with NSAIDs overuse. As a non-opioid analgesic, SRP-001 lacks abuse potential, which later clinical trials will further confirm. These aspects of SRP-001’s safety profile underscore its potential as a significant advancement as a novel pain therapeutic candidate, aligning with the increasing clinical demand for safer analgesic options.

Despite the widely recognized need for safer and more effective pain medications is widely recognized, particularly considering the opioid epidemic in the U.S., the lack of innovation in this space has left patients with limited options. Backpedaling on 2016 guidelines urging physicians to restrict the use of opioids for moderate-to-severe acute and chronic pain, the Centers for Disease Control and Prevention recently updated its Clinical Practice Guidelines for Prescribing Opioids^69^. The high failure rate of novel pain therapeutics in clinical studies compared to other fields in medicine, the limited availability of investor funding due to this increased risk, and the poor appetite for pain therapeutics among pharmaceutical companies^70^ are causes for marginal innovation toward safer and effective pain therapeutics. In order to reduce the developmental risk, these considerations emphasize the significance of determining whether a novel pain therapeutic candidate alleviates pain via clinically validated MOA. Thorough pre-clinical safety evaluations, AM404 and transcriptomic studies showcasing a validated, clinically established MOA similar to ApAP, combined with Phase 1 safety data, collectively suggest that advancing the development of SRP-001 could potentially provide a safe and effective pain relief option for acute and chronic pain in humans, characterized by a large therapeutic window.

### Limitations

In the present study, we demonstrate a well-matched alignment between behavioral assays and the neurobiological region of focus, specifically the midbrain. Upcoming research will integrate pain-avoidance assays in rats, developed in-house, to more accurately quantify the motivational aspects of pain. These assays aim to capture higher limbic elements, such as cortical regions, associated with pain and pain-related negative affective-like behavior. Besides the PAG region, there are other brain regions in pain processing, notably the amygdala, which is also crucial in pain modulation, playing a pivotal role in the affective-emotional dimensions of pain. It intricately links with other forebrain structures that modulate the emotional aspects of pain and cognitive and motivational responses. While we focused on the PAG region due to its established role in the modulation of both opioid and non-opioid analgesia, the importance of broader limbic structures will be the subject of future work on SRP-001’s antinociceptive effects. Also, analgesic compounds such as opioids and alcohol consistently elevate paw withdrawal thresholds above baseline, corroborated by our findings with ApAP. For SRP-001, no overt alterations in locomotor activity or motor incoordination are evident. Future investigations will rigorously assess potential motor and other side effects of SRP-001 in both pre-clinical and human models. Finally, although pre-clinical data shows SRP-001's efficacy in three antinociception assays and we elucidate its mechanism of action, only upcoming Phase 2 trials can confirm its clinical analgesic efficacy.

## Methods

### Experimental

We recently synthesized a library of 2-(benzenesulfonamide)-N-(4-hydroxyphenyl) acetamide analgesics in search of non-hepatotoxic ApAP analogs^28^. From this library, **SRP-001** was chemically synthesized using readily available commercial analytical grade reagents of highest quality, which were purchased and used without further purification. Melting points were determined in open capillary tubes on a Stuart Scientific SMP3 melting point apparatus. Mercury VX-300, Bruker BioSpin GmbH 400 MHz, or Varian Unity 500 MHz spectrometers were used to record ^1^H and ^13^C NMR spectra at room temperature. Chemical shifts are given in ppm (δ) downfield from TMS. Coupling constants (*J*) are in hertz (Hz), and signals are described as follows: s, singlet; d, doublet; t, triplet; br, broad; m, multiplet. Mestrenova 12.0.4 software was used to analyze the NMR FIDs. Purity of the products was determined by chromatographic analysis using an Agilent 1200 with diode array detector and Agilent 1100 MSD-Q mass detector (Column C18 Luna, 100 mm × 4.6 mm × 3 µm. Mobile phase A: water with 0.1% of formic acid. Mobile phase B: MeOH with 0.1% of formic acid. Elution gradient: 5% B to 100% B in 20 min. Flow: 1 mL/min with Split 1:2 for MS detection. UV wavelengths: 214, 254 nm. Mass Detection: Scan 50–1000 m/z.). *N*-(4-Hydroxyphenyl)-2-(1,1,3-trioxo-1,2-benzothiazol-2-yl) acetamide **1** was synthesized as previously described^71^.

### N,N-diethyl-2-[[2-(4-hydroxyanilino)-2-oxo-ethyl]sulfamoyl]benzamide (SRP-001)

To *N*-(4-hydroxyphenyl)-2-(1,1,3-trioxo-1,2-benzothiazol-2-yl) acetamide **1** (0.165 g, 0.496 mmol), a solution of diethylamine (0.154 mL, 1.5 mmol) in acetonitrile (3 mL) was added. The mixture was refluxed for 16 h. Evaporation under reduced pressure gave a brown residue, which was purified by chromatography [silica gel60 F254, 70–200 mm, ethyl acetate:hexane (6:4)], followed by crystallization from ethyl acetate:hexane, yielding **SRP-001** as a white solid (0.132 g, 66%). mp 171 – 172 °C; ^1^H NMR (400 MHz; DMSO‑d_6_; Me_4_Si) δ (ppm): 9.69 (s, 1H), 9.18 (s, 1H), 7.91 (dd, *J* = 7.6, 1.5 Hz, 1H), 7.73 – 7.54 (m, 2H), 7.46 (brs, 1H), 7.43 (dd, *J* = 7.4, 1.5 Hz, 1H), 7.22 (d, *J* = 8.8 Hz, 2H), 6.64 (d, *J* = 8.8 Hz, 2H), 3.77 – 3.46 (m, 3H), 3.38 – 3.28 (m, 1H), 3.16 – 2.95 (m, 2H), 1.17 (t, *J* = 7.0 Hz, 3H), 1.02 (t, *J* = 7.1 Hz, 3H); ^13^C NMR (101 MHz, DMSO‑*d*_6_) δ (ppm): 168.4, 165.4, 153.5, 136.3, 135.3, 132.8, 130.0, 129.3, 128.6, 127.3, 121.0 (2C), 115.0 (2C), 45.6, 42.7, 38.3, 13.1, 11.9; purity by HPLC 99.1%; MS (ESI^+^) (m/z) 406.20 (MH^+)^. Anal. Calcd. for C_19_H_23_N_3_O_5_S: %C 56.28, %H 5.72, %N 10.36, %S 7.91. Found: %C 56.73, %H 5.85, %N 10.56, %S 8.17.
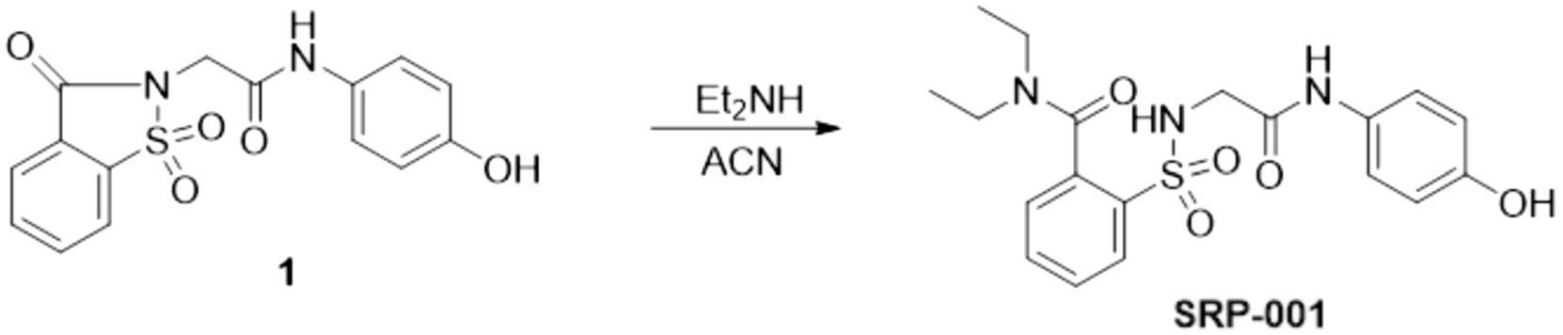


### Serum isolation for NAPQI identification, characterization of novel benzoquinoimine, and liver function tests (LFTs)

Male CD1 mice were fasted overnight for 15 h and dosed with ApAP or **SRP-001** or vehicle (0.9% saline) at doses of 600 mg/kg administered via *per os* (*PO*) injections with an administration volume of 10 mL/kg body weight. After drug administration, animals were returned to their respective cages and maintained with food and water provided ad libitum for the next 12 h. Animals were then euthanized under 5% isoflurane anesthesia after 12 h, and whole blood samples were collected transcardially in sterile microcentrifuge tubes without anti-coagulants. Whole blood samples were stored at room temperature (25 °C) for 30 min, allowing them to coagulate, which was centrifuged at 1000 g for 5 min at 4 °C to isolate serum samples from whole blood, which were collected, aliquoted, and stored at − 80 °C for identification of NAPQI, and liver function tests (LFTs).

### LC–MS/MS method for NAPQI identification and characterization of novel benzoquinoimine

N-acyl-p-benzoquinone imine (NAPQI) was extracted from the serum by adding 3 volumes of ethyl-acetate to 1 volume of serum and storing them on ice for 30 min, followed by centrifugation at 3000*g* for 30 min. Afterward, the supernatant was transferred into mass spectrometry vials and dried under N_2_ gas. The serum was then washed with another 3 volumes of ethyl-acetate, and the supernatant was added back to the same mass spectrometry vial, followed by evaporation under a stream of N_2_ gas. The sample was re-suspended with 50 µl of 1:1 MeOH:H_2_O for LC–MS/MS experimentation. The mass spectrophotometer was operated in multiple reaction monitoring (MRM) mode using positive ion electrospray. NAPQI was detected by monitoring the m/z transition 150.3 → 108.1. Pure NAPQI analytical standard (10 ng/mL) obtained from Cayman Chemical (Ann Arbor, MI) was used to characterize the full fragmentation pattern of NAPQI and determine the likely fragments.

### LC–MS/MS methods for detection of D10-SRP-001 and AM404 in in vivo pain models (changed order so that both LC–MS/MS methods are together)

Deuterated compound **D**_**10**_**-SRP-001** was synthesized by Olon Ricerca Bioscience (Concord, OH), and purity was determined to be > 99.9% by HPLC. This was used as pure analytical standard for detection of SRP-001 using an LC–MS/MS method. Similarly, pure analytical standard of AM404 (10 ng/mL) obtained from Cayman Chemical (Ann Arbor, MI) was used to characterize the full fragmentation pattern of AM404. Vehicle (0.9% saline), ApAP or **SRP-001** was injected *ip* at 32 mg/kg into Sprague–Dawley rats. Thirty min post-injection, the animals were sacrificed (same as in the CFA/von Frey model), their brains harvested, and the midbrain periaqueductal gray (PAG) region excised. **SRP-001** or AM404 were extracted from the PAG using a liquid–liquid extraction method using 1:1 MeOH:H_2_O for LC–MS/MS and were loaded onto a liquid chromatography-tandem mass spectrophotometer for analysis. The mass spectrophotometer was operated in multiple reaction monitoring (MRM) mode using positive ion electrospray. **D**_**10**_**-SRP-001** was detected by monitoring the m/z transition 416 → 186. AM404 was detected by monitoring the m/z transition 396 → 287.

### Liver function tests (LFTs)

Liver transaminases – Alanine Aminotransferase (ALT), and Aspartate Aminotransferase (AST), were measured using fresh serum samples by commercially available ELISA kits from Sigma-Aldrich and Abcam, according to the manufacturer’s suggested protocols. LFTs were done on fresh serum samples, and not on thawed frozen serum samples to avoid denaturing LFT transaminases due to freeze/thaw of frozen serum.

### Histology and immunohistochemistry for Nitrotyrosine, hepatic tight junctions, and TUNEL apoptosis assays

Male CD1 mice fasted overnight with only access to water ad libitum were dosed with either ApAP, or **SRP-001** or vehicle (0.9% saline) (n = 5 for each treatment group) via *PO* dosing at concentrations of 600 mg/kg (for both ApAP and SRP-001) with an injection volume of 10 mL/kg body weight. We selected this dosage based on its well-documented characterization in previous research as an effective measure for evaluating ApAP-induced hepatotoxicity in preclinical rodent models^33–35,59,72,73^.

Employing a consistent lethal dose for both compounds streamline the experimental design and facilitates data interpretation. This approach aligns with FDA guidelines on Bioequivalence and Bioavailability studies for estimating the maximum safe starting dose in humans, a strategy further substantiated by others^74,75^. At 12 h post-injection, mice were deeply anesthetized under 5% isoflurane for more than 5 min and formalin-fixed via transcardial perfusion with 10% Neutral Buffered Formalin (NBF) after exsanguination using 0.9% NaCl. Liver tissues were extracted and stored in NBF for 24 h post fixation, after which they were then transferred to 80% EtOH for storage prior to paraffin embedding, sectioning, and immunological staining. FFPE blocks were sectioned into 5 µm thick slices on regular frost-free plus slides and dried overnight on a slide warmer. After deparaffination, sections were stained with validated antibodies for nitrotyrosine and ZO-1 (tight junctions). For detection of apoptotic nuclei by TUNEL staining, we used Promega’s DeadEnd fluorometric TUNEL assay, following the manufacturer’s suggested protocol. Nitrotyrosine labeling was detected with 3′3’-Diaminobenzidine (DAB) staining, and nitrotyrosine labeled hepatic sections were imaged at 100 × magnification in brightfield using a Nikon Eclipse TS100 microscope with NIS-Elements BR 3.0 software (NIKON Inc, Melville, NY, USA). Slides with ZO-1 staining were imaged in z-stacks obtained at 20 × magnification using Olympus FV-1200 confocal microscope with Fluoview software FV10-ASW Version 04.02.02.09 (Olympus Corp Center Valley, PA, USA). Quantification of ZO-1 staining hepatic tight junctions was carried out by unbiassed image analysis calculating area sum of pixel^2^ of the green channel staining ZO-1using CellSens software (Olympus Corp Center Valley, PA, USA). TUNEL-positive apoptotic nuclei were counted in 15 random fields from hepatic sections obtained from mice treated with each compound—vehicle, ApAP, or **SRP-001—**using ImageJ software 1.48 (National Institutes of Health).

### Animal experiments

All animal protocols and procedures were completed under the pre-approved provisions of the Institutional Animal Care and Use Committee (IACUC) of Louisiana State University Health Sciences Center (LSUHSC), New Orleans. The experiments were performed under the approval by the Institutional Animal Care and Use Committee, LSU Health New Orleans, IACUC protocol #3739. In vivo antinociception was tested in two different strains of mice: CD1 and C57BL/6 mice and Sprague–Dawley rats. All laboratory rodents were purchased from a commercial vendor (Charles River); animals were acclimated to the LSUHSC New Orleans Neuroscience Center of Excellence vivarium for at least seven days before experimental protocols began. All animals were kept in a 12 h day-night cycle with food and water available ad libitum.

### Equimolar dose–response toxicity analysis

To accurately compare the toxicity profiles of ApAP and SRP-001, we conducted an equimolar dose–response toxicity analysis. CD-1 male mice obtained from Charles River Laboratories underwent a one-week acclimation period. They were housed under a 12-h day/night cycle, with unrestricted access to food and water. Before experimentation, mice were fasted for 15 h. Subsequently, they were administered either a vehicle (placebo), ApAP, or SRP-001 in equimolar doses of 992, 1984, 3969, or 5954 mM/kg, respectively, via oral gavage. This corresponds to an increasing concentration of 150, 300, 600, and 900 mg/kg for ApAP and 402, 804, 1609, and 2414 mg/kg for SRP-001. Each treatment group consisted of 10 mice. Post-dosing, the mice were monitored at 6-h intervals over 72 h for signs of becoming moribund. An investigator blinded to the treatment groups conducted the moribund state assessment.

### In vivo analgesia models and equimolar (µmol/kg) ED_50_ calculation

We explored the antinociceptive properties of **SRP-001** using the oral nanosuspension of **SRP-001**. The oral nanosuspension formulation containing 100 mg/mL **SRP-001** in preserved aqueous 1% hydroxypropyl cellulose (1% HPC) (**Lot# LPI-2021028**) was compared to Acetaminophen (ApAP) 100 mg/mL and a vehicle control (aqueous 1% HPC) also given as suspension, in three different in vivo pain animal models – namely, Complete Freund’s Adjuvant (CFA) induced inflammatory, tail flick somatic, and abdominal writhing visceral assays, along with electronic von Frey (eVF) and Hargreaves determination of mechanical and thermal (noxious heat) sensitivity, respectively.

The investigators were blinded till the completion of all the experiments. To ensure objectivity, investigators were blinded for the acetic acid and tail flick experiments. In the CFA/von Frey and Hargreaves assays, one investigator did the drug pre-treatments, and another investigator at another location who was not aware of the treatments conducted the behavioral testing. Moreover, these studies used the eVF device that electronically registers the grams of force necessary to elicit paw withdrawal, eliminating bias that confounded older versions of this test in which an investigator used a series of individual von Frey microfilaments to determine paw withdrawal thresholds. Six separate experimenters were involved in the data acquisition for behavioral testing, and another separate investigator performed all the statistical analyses using GraphPad Prism Version 9.1.2. Statistical significance was determined by *p* < *0.05*; one-way ANOVA followed by Sidak’s multiple comparisons post hoc test. Mixed-gender experiments were powered to test for gender effects, and different ages of rodents were also included for the experiments – young (2 months) and aged (20 months) to tease out whether there are any effects of age-related changes in nociceptive sensitivity under naïve, and inflammatory pre-clinical rodent pain models. For the aged rats and mice, we could only obtain male Sprague–Dawley rats and male C57BL/6 mice of 20 months old from all commercial animal vendors and from the National Institute of Aging (NIA). So, only these rodents were used for the older animal experimental cohort. Old female rats and mice were not available at the time when these experiments were conducted. For ED_50_ calculation, dose–response curves were calculated with regression analysis and log transformation using GraphPad Prism 9.1.2.

### CFA/von Frey with electronic detection (eVF)

First, we used male Sprague–Dawley rats and the CFA inflammatory pain assay with electronic von Frey detection to assess antinociceptive/anti-hyperalgesic efficacy of **SRP-001** compared to ApAP and vehicle control. In this model of mechanonociception, one hind paw at a time is stimulated with an electronic von Frey (eVF) filament (noxious source) until the animal retracts the paw from the mechanical stimulus. von Frey tests were conducted in a dedicated room at the LSUHSC New Orleans Neuroscience Center of Excellence vivarium. Rats were acclimated to their environment for 2 days for 30 min/day prior to testing. To obtain eVF pressure recordings, each animal was placed in an individual plastic observation compartment on a perforated metallic grid platform, which provided access to the plantar surface of the hind paws. After acclimation to the environment for 30 min, mechanical hypersensitivity was assessed by stimulating the mid-plantar area of each hind paw with a rigid tip von Frey filament attached to the eVF meter (Ugo Basile 38,450) until animals withdrew the paw from the filament. The withdrawal threshold was defined as the average force/pressure (g) required for the rat to withdraw the stimulated paw. A brisk withdrawal of the paw (often followed by a sustained retraction and/or licking) was considered a positive response, but paw withdrawals due to locomotion or weight shifting were not counted. The von Frey studies were conducted with an eVF device that electronically registers the grams of force necessary to elicit paw withdrawal in order to eliminate bias that confounded previous older versions of this test. Baseline withdrawal thresholds of both the right and left paws were recorded, and animals were assigned to different treatment groups so that each group had approximately equal withdrawal threshold averages in both paws. After baseline testing, treated animals received subcutaneous plantar injection (150 μl) of 50% CFA diluted and freshly mixed in sterile (0.9%) saline into the left hind paw unilaterally, while control animals received a plantar injection (150 µl) of 0.9% NaCl into their left hind paw. CFA induces inflammation, resulting in a left hind paw that is hypersensitive to mechanical stimulus, while the right hind paw serves as a within-subject baseline for each animal. In the experimental setup, the left hind paw of rats was administered either CFA or saline, while the right hind paw functioned as an uninjected control in CFA-treated animals for each pharmacological agent, be it ApAP or SRP-001. On the day of testing, each animal received the drugs – ApAP or **SRP-001** via *PO* administration based on their assigned treatment groups. Doses at concentrations of 32 mg/kg and 100 mg/kg were tested in a cumulative dose–response manner and given at an interval of 60 min, and paw withdrawal threshold readings were measured using eVF as described above.

Additionally, we determined ED50 values on an equimolar (µmol/kg) basis for both ApAP and SRP-001 across all in vivo antinociception assays (Fig. 2j, and Supplementary Fig. 5).

Drawing upon our previously published work, wherein dose–response curves for ApAP and its novel analogs were established across various dosages—10, 32, 100, and 320 mg/kg^28^, we selected median doses of 32 and 100 mg/kg for the current study. Our experimental design adhered to the principles of the 3Rs—Replacement, Reduction, and Refinement—as outlined by Russel and Burch, aiming to minimize 'unnecessary duplication' and reduce the number of animals needed for robust, replicable data of 3Rs^76–80^. Additionally, we determined ED_50_ values on equimolar (µmol/kg) basis for both ApAP and SRP-001 across all in vivo antinociception assays (Fig. 2j, and Supplementary Fig. 5).

#### Young male rats

In this cohort, n = 40 male Sprague–Dawley rats (2 months) were used, and two different doses of **SRP-001** oral nanosuspension and ApAP (32 and 100 mg/kg) were compared to a vehicle control.

#### Young female rats

In this cohort, n = 40 female Sprague–Dawley rats (2 months) were used, and two different doses of **SRP-001** oral nanosuspension and ApAP – 32 and 100 mg/kg were compared to a vehicle control.

#### Aged male rats

In this cohort, n = 20 male Sprague–Dawley rats (20 months) and two different doses of **SRP-001** oral nanosuspension and ApAP – 32 and 100 mg/kg were compared to a vehicle control.

### Tail flick

We used cold stimulation tail flick somatic pain assay testing 2 different doses of ApAP or **SRP-001** at 32 m/kg or 100 mg/kg to measure increased tail withdrawal time (latency) in CD1 (young male and female) or C57BL/6 (aged male) mice to cold stimulation of the tail tip. Half of the tail was immersed in ice-cold water maintained at 4 °C. For the tail-flick assays, we used n = 70 aged male mice, n = 90 young male mice, and n = 120 young female mice. Based on the availability of mice at the time of the experiments, different numbers of animals were assigned to different cohorts of experimental animals. Tail flick assay experiments were conducted on an open bench in a dedicated room at the LSUHSC New Orleans Neuroscience Center of Excellence vivarium. Mice were allowed to acclimate to the laboratory environment for 1 h prior to testing. To restrain the mice for the test, disposable plastic 50 mL screw-capped conical centrifuge tubes were cut at the tip to create a 0.5 cm opening to allow the mice to breathe freely. Another 0.5 cm opening was cut into the cap to allow access of the tail to the water bath. A 500 mL glass beaker was filled with 450 mL of ice-cold distilled water maintained at 4 °C with the addition of ice and determined with a glass thermometer. Mice were held over the opening of the water bath, and their tails submerged approximately halfway into the water. The nociceptive threshold was taken as the latency until the mice flicked their tail tip or removed the tail. The time from immersion to the attempted tail tip removal was measured to 1/10^th^ of a sec with a digital laboratory timer. To minimize damage to the tail, a 60-s cut-off was utilized. After baseline measurements, mice were dosed with ApAP or **SRP-001** via *PO* administration based on their assigned treatment groups. Injections were at doses of 32 mg/kg and 100 mg/kg. 30 min post-drug administration tail-flick/withdrawal latency was measured.

### Abdominal writhing assay

In this model of visceral pain, abdominal contraction (writhing), in which contraction of the abdominal muscles and stretching of the hind limbs is induced in mice in response to an *ip* injection of 0.4% acetic acid at a dose of 10 mL/kg 25 min after drug administration, is assessed. Specifically, the number of writhes is counted for 10 min, beginning 5 min after acetic acid injection^81^. All animals were fasted overnight (15 h) prior to testing, and the compounds were administered via *PO* administration to animals belonging to the treatment groups – ApAP or **SRP-001** – and tested at doses of 32 mg/kg and 100 mg/kg, respectively. CD1 (young male and female) or C57BL/6 (aged male) mice were used. For the abdominal writhing assays, we used n = 35 aged male mice, n = 70 young male mice, and n = 70 young female mice.

### CFA-induced inflammatory pain/Hargreaves thermal sensitivity antinociception assay

First, we used male Sprague–Dawley rats and the CFA inflammatory pain assay with eVF detection to assess antinociception/anti-hyperalgesic efficacy of **SRP-001** compared to ApAP and vehicle control. In these same cohorts of animals, we simultaneously measured their thermal nociception to hyperalgesia with the Hargreaves test using the plantar test apparatus (Ugo Basile 37,570). In this experimental setup, the rodents are placed in plastic cages on top of a glass surface, and their hind paws are subjected to an infrared heat stimulus. One hind paw at a time is stimulated with the light source (noxious stimuli) until the animal retracts the paw from the glass surface because of the stimulus. Baseline withdrawal thresholds (latency) of both the right and left paws were recorded, and animals were assigned to different treatment groups so that each group had approximately equal withdrawal threshold averages in both paws. After baseline testing, treated animals received subcutaneous plantar injection (150 μl) of 50% CFA into the left hind paw, while control animals received a plantar injection (150 µl) of 0.9% NaCl into their left hind paw. CFA induces inflammation, resulting in a left hind paw that is hypersensitive to the thermal stimulus, while the right hind paw serves as a within-subject baseline for each animal. To obtain Hargreaves withdrawal latency recordings, each animal was placed in an individual plastic observation compartment on a glass platform, which provided access to the plantar surface of the hind paws from underneath. After acclimation to the environment for 30 min, thermal hypersensitivity was assessed by stimulating the mid-plantar area of each hind paw with a bright light source attached to the meter (Ugo Basile 37,570) until animals withdrew the paw from the surface. The withdrawal threshold was defined as the average time (s) required for the rat to withdraw the stimulated paw. A brisk withdrawal of the paw (often followed by a sustained retraction and/or licking) was considered a positive response, but paw withdrawals due to locomotion or weight shifting were not counted. The Hargreaves studies were conducted with the plantar stimulation device that automatically registers the time (s) necessary to elicit paw withdrawal in order to eliminate bias in determining paw withdrawal thresholds. A cutoff of 20 s is pre-programmed so that the light source shuts off at that maximal period so as to not induce any burns on the plantar surface of the rodents. The Hargreaves test permits measurement of ipsilateral and contralateral heat thresholds, allowing each animal to serve as its own internal control in unilateral pain models. In addition, the Hargreaves test enables quantification of heat thresholds in unrestrained animals, reducing the likelihood of stress-induced responses.

#### Young male rats

In this cohort, n = 40 male Sprague–Dawley rats (2 months) were used, and two different doses of **SRP-001** oral nanosuspension and ApAP – 32 and 100 mg/kg were compared to a vehicle control.

#### Young female rats

In this cohort, n = 40 female Sprague–Dawley rats (2 months) were used, and two different doses of **SRP-001** oral nanosuspension and ApAP – 32 and 100 mg/kg were compared to a vehicle control.

#### Aged male rats

In this cohort, n = 20 male Sprague–Dawley rats (20 months) were used, and two different doses of **SRP-001** oral nanosuspension and ApAP – 32 and 100 mg/kg were compared to a vehicle control.

### In vivo antipyresis assays

Antipyresis experiments were carried out with n = 60 CD-1 male mice weighing between 45 and 50 g. All mice were kept in a 12-h day/12-h night cycle with free access to food and water ad libitum. After habituating and acclimating the mice for a week, each mouse was briefly anesthetized for 1 min with 1% isoflurane and implanted subcutaneously a transmitter probe that records core body temperature – Implantable Programmable Temperature Transponder (IPTT-300) by Bio Medic Data Systems (BMDS), DE, USA. The mice were allowed to recover for at least one week from the surgery before any recordings were made and observed in their home cages prior to pyrogen challenge for inducing fever. Temperatures were recorded using the BMDS DAS-8027-IUS data reading system.

### Pyrogen (LPS) injections

All mice were fasted overnight (15 h) with free access to water ad libitum prior to the pyrogen challenge. For the induction of fever, LPS from *E. coli* (100 μg/kg, 0111:B4) (MilliporeSigma, Billerica, MA, USA) was used. Mice received either LPS in 0.9% saline (sterile) (vehicle) or just 0.9% saline as an intraperitoneal (*ip*) injection. Mice were returned back to their home cages post-injection and observed, and core body temperatures were recorded at the 4-h mark post-injection, and with the development of fever (~ at least 1 degree change from basal core body temperature), febrile mice were selected and grouped in different groups for drug administration, and temperatures were recorded 2-h post drug administration to determine the antipyretic effects of ApAP or **SRP-001**. All mice that did not show a significant change in body temperature or appeared sick post-pyrogen challenge (n = 5) were excluded from subsequent drug administrations. No mice were excluded from the treatment groups that were dosed with either ApAP or SRP-001 (75 mg/kg).

#### Drug injection/concentration

All drugs and vehicle (10% DMSO: 10% labrasol :80% PEG400) were administered by oral gavage, *PO* dosing. Each dosing volume was ~ 0.3–0.4 mL per mouse. ApAP, SRP-001 and vehicle were all prepared as suspensions. The dose for ApAP and the novel compound, SRP-001, used for this trial was 75 mg/kg. We chose this dose to record antipyretic effect in a low dose that doesn’t cause hypothermia in mice. Febrile mice were grouped into different control and experimental groups as follows:

- Normal control (Baseline) – 0.9% Saline (*ip*) and 0.9% saline (*po*) (n = 10)
- Positive control (ApAP) – LPS (100 μg /kg) (*ip*) and APAP (75 mg/kg) (*po*) (n = 20)
- **SRP-001** – LPS (100 μg /kg) (*ip*) and SRP-001 (75 mg/kg) (*po*) (n = 20)

### Baker yeast-induced hyperthermia

15% yeast, 0.1 mL/kg (MilliporeSigma, Billerica, MA, USA) was used to induce fever. All animals used in this assay were male CD1 mice, which were fasted overnight (15 h) before the start of the assay.

Mice received either yeast in 0.9% saline (sterile) (vehicle) or just 0.9% saline as an *ip* injection. Mice were returned back to their home cages post-injection and observed, and core body temperatures recorded at the 4-h mark post-injection, and with the development of fever (~ at least 1degree change from basal core body temperature), febrile mice were selected and grouped in different groups for drug administration, and temperatures were recorded 2 h post drug administration to determine the antipyretic effects of ApAP or **SRP-001**. All mice that didn’t show a significant change in body temperature or appeared sick post-pyrogen challenge were excluded (n = 5) from subsequent drug administrations. No mice were excluded from the treatment groups that were dosed with either ApAP or SRP-001 (75 mg/kg).

#### Drug injection/concentration

All drugs and vehicle (10% DMSO: 10% labrasol :80% PEG400) were administered by oral gavage, *PO* administration. Each dosing volume was ~ 0.3–0.4 mL per mouse. ApAP, SRP-001 and vehicle were all prepared as suspensions. The dose for ApAP and the novel compound, SRP-001, used for this trial was 75 mg/kg. We chose this dose to record antipyretic effect in a low dose that doesn’t cause hypothermia in mice. Febrile mice were grouped into different control and experimental groups as follows:

- Normal control (Baseline) – 0.9% Saline (*ip*) and 0.9% saline (*po*) (n = 10)
- Positive control (ApAP) – yeast (15%) (*ip*) and APAP (75 mg/kg) (*po*) (n = 10)
- **SRP-001** – yeast (15%) (*ip*) and SRP-001 (75 mg/kg) (*po*) (n = 10)

### Cardiotoxicity assay (hERG) and cardiovascular effect in conscious telemetered beagle dogs (GLP study)

The effects of SRP-001 on hERG current amplitude at physiologic temperatures were evaluated over a nominal concentration range of 0.1 μM to 100 M. A vehicle group (10% DMSO) was included in the study for comparison, and dofetilide (10 nM) was used as a reference substance. The vehicle and positive controls indicated appropriate sensitivity of the test systems. SRP-001 was a weak blocker of hERG current with the highest concentration tested (100 μM) blocking by 21.4 ± 3.9%. Next, the potential acute effects of oral SRP-001 on cardiovascular function in conscious telemetered beagle dogs was done using a Latin Square design; 4 male naïve beagle dogs each received vehicle (10% DMSO: 10% labrasol: 80% PEG400) and three doses (30, 90, and 150 mg/kg) of SRP-001 nanosuspension by oral gavage. The animals were observed for clinical signs pre-dose, immediate post-dose, and approximately 24 h post-dose administration. A single dose was administered on the day of dosing, and a washout period of seven days was allowed between doses. One-min means of hemodynamic parameters, as well as ECG parameters and body temperature, were measured for a period of at least 24 h. One-min tracings of the ECGs were obtained at 15 min prior to dosing and at 30 min, 1, 2, 4, 8, 12, and 24 h post-dose. Qualitative evaluation of the ECGs was done by a board-certified veterinary cardiologist.

### Pulmonary function evaluation in conscious male Sprague–Dawley rats (GLP study)

The potential effects of orally administered SRP-001 on respiratory rate, tidal volume, and minute volume in conscious male Sprague–Dawley rats was studied in 24 male rats. The rodents were trained for two days in the head-out plethysmograph chamber for 15–17 min each prior to the experiment. On the day of dosing, each animal was weighed and placed in the plethysmograph chamber, and baseline respiratory parameters were obtained for 5 min following an approximately 5-min stabilization period. The rats were then removed from the chamber and dosed by oral gavage. Three groups of six male Sprague–Dawley rats each were orally administered SRP-001 at a single dose of 220, 440, or 660 mg/kg at a dose volume of 10 mL/kg. An additional group of six rats was administered the vehicle (10% Labrasol: 10% DMSO: 80% PEG400) at 10 mL/kg. Following dosing, each animal was returned to its designated plethysmograph chamber, and the respiratory parameters were recorded at 30 min (± 5 min), 2, 4, and 6 h (± 15 min). Animals were allowed to stabilize in the plethysmograph chamber for at least 5 min before each reading was taken. The following parameters were acquired, recorded, and analyzed using Ponemah Physiology Platform (Ponemah v.5.20 Pulmonary); Respiratory rate, Tidal Volume, and Minute Volume, Values for the test article treated group were compared to the vehicle control values using a two-way repeated measures ANOVA followed by a Bonferroni Multiple Comparison Test (SigmaStat, v. 4.0). Differences with p values ≤ 0.05 were considered statistically significant.

### Neuropharmacological profile in Sprague–Dawley rats (GLP study)

Any potential neuropharmacological effects of orally administered SRP-001 in rats were assessed in three groups of eight male Sprague–Dawley rats. Each was orally administered SRP-001 at a single dose of 220, 440, or 660 mg/kg. Vehicle (10% labrasol: 10% DMSO: 80% PEG400) was administered to an additional group of eight rats. The dose volume was 10 mL/kg. The rats were observed for neuropharmacological or other clinical signs using a modified Irwin/Functional Observational Battery test at 30 min and 1, 2, 4, and 24 h following treatment. All observations were made at ± 5 min for the observations through 1 h post-dose and ± 15 min for the later observations. Body temperature was measured 1 h following dose administration. No apparent neuropharmacological or clinical signs were observed through 24 h post-dose in any rats receiving either vehicle at 10 mL/kg or SRP-001 at 220, 440, or 660 mg/kg. Body temperature was not affected by the oral administration of SRP-001 at 220 or 440 mg/kg at 1 h post-dose when compared to the vehicle control group. However, the body temperature was significantly (p ≤ 0.05) lower in the high-dose group (660 mg/kg) compared to the vehicle group.

### Genotoxicity

Results from both the Ames and micronucleus assays indicate that SRP-001 is negative for genotoxicity induction (Supplementary Table 1). However, the chromosomal aberration assay results indicated that SRP-001 was positive for the induction of chromosomal aberrations in vitro. To further evaluate the potential genotoxicity of SRP-001 and determine the validity of the in vitro findings, a second in vivo assay, a GLP-compliant Comet assay, was conducted in accordance with ICH S2(R1) Guidance on Genotoxicity Testing and Data Interpretation for Pharmaceuticals Intended for Human Use. SRP-001 did not cause DNA damage in the liver or stomach of male rats administered up to 2,000 mg/kg and was concluded to be negative in the in vivo Comet assay. Based on the weight of the evidence, it is concluded that SRP-001 is negative for genotoxicity. This is based on the following:

#### A negative Ames mutagenesis demonstrates SRP-001 non-mutagenic

SRP-001 was tested to evaluate its mutagenic potential by measuring its ability to induce reverse mutations at selected loci of several strains of *Salmonella typhimurium* (*S. typhimurium*) and at the tryptophan locus of *Escherichia coli* (*E. coli*) strain WP2 *uvr*A in the presence and absence of an exogenous metabolic activation system (Bioreliance, Rockville, MD USA). Dimethyl sulfoxide (10% DMSO) was used as the vehicle. The dose levels tested in the preliminary toxicity assay were 6.67, 10.0, 33.3, 66.7, 100, 333, 667, 1000, 3333, and 5000 µg per plate. No precipitate was observed. Toxicity as a reduction in the revertant count was observed at 5000 µg per plate with tester strains TA1537 and WP2 *uvr*A in the absence of S9 activation. Based on these results, the maximum dose tested in the mutagenicity assay was 5000 µg per plate. The dose levels tested in the mutagenicity assay were 100, 333, 667, 1000, 3333, and 5000 µg per plate. Neither precipitate nor toxicity was observed. No positive mutagenic responses were observed with any of the tester strains in either the presence or absence of S9 activation. Collectively, these results indicate SRP-001 is negative for the ability to induce reverse mutations at selected loci of several strains of *S. typhimurium* and at the tryptophan locus of *E. coli* strain WP2 *uvr*A in the presence and absence of an exogenous metabolic activation system.

#### In vitro mammalian chromosomal aberration assay in chinese hamster ovary cells

In the preliminary toxicity assay, the doses tested ranged from 0.0405 to 405 µg/mL (1 mM), which was the limit dose for this assay (Bioreliance, Rockville, MD USA). Cytotoxicity (≥ 45% reduction in cell growth index relative to the vehicle control) was not observed at any dose in the non-activated and S9-activated 4-h exposure groups. Cytotoxicity was observed at doses ≥ 122 µg/mL in the non-activated 20-h exposure group. Based on these results, the doses chosen for the chromosome aberration assay ranged from 75 to 405 µg/mL for the non-activated and S9-activated 4-h exposure groups and from 5 to 125 µg/mL for the non-activated 20-h exposure group.

In the chromosome aberration assay, cytotoxicity (≥ 45% reduction in cell growth index relative to the vehicle control) was not observed at any dose in the S9-activated 4-h exposure group. Cytotoxicity was observed at 405 µg/mL in the non-activated 4-h exposure group and at doses ≥ 70 µg/mL in the non-activated 20-h exposure group. The doses selected for evaluation of chromosome aberrations were 150, 300, and 405 µg/mL for the non-activated and S9-activated 4-h exposure groups; and 10, 40, and 80 µg/mL for the non-activated 20-h exposure group. In the non-activated 4-h exposure group, statistically significant and dose-dependent increases in structural aberrations (4.3% and 12.7%) were observed at doses 300 and 405 µg/mL, respectively (p < 0.01; Fisher’s Exact test and p 0.05; Cochran-Armitage test). The induction of structural aberrations was outside the 95% control limit of the historical negative control data of 0.00% to 2.79%. In the S9-activated 4-h exposure group, statistically significant and dose-dependent increases in structural aberrations (3.7% and 3.3%) were observed at doses 300 and 405 µg/mL, respectively (p < 0.05; Fisher’s Exact and Cochran-Armitage tests). However, the induction of structural aberrations was within the 95% control limit of the historical negative control data of 0.00% to 4.28%. In the non-activated 20-h exposure group, a statistically significant and dose-dependent increase in structural aberrations (3.7%) was observed at 80 µg/mL (p < 0.01; Fisher’s Exact test and p < 0.05; Cochran-Armitage test). The induction of structural aberrations was outside the 95% control limit of the historical negative control data of 0.00% to 2.68%.

Neither statistically significant nor dose-dependent increases in numerical (polyploid or endoreduplicated cells) aberrations were observed at any dose in treatment groups with or without S9 (p > 0.05; Fisher’s Exact and Cochran-Armitage tests). The induction of numerical aberrations was within the 95% control limit of the historical negative control data. These results indicate SRP-001 was positive for the induction of structural chromosomal aberrations and negative for the induction of numerical chromosomal aberrations in the presence and absence of the exogenous metabolic activation system.

#### Clastogenicity and rat micronucleus; in vivo study: SRP-001 is deemed negative (non-clastogenic)

Male rats were dosed at 500, 1000, and 2000 mg/kg/d once per day on two consecutive days at 10 mL/kg via oral gavage using 10% labrasol, 10% DMSO, and 80% PEG400 as the vehicle (Bioreliance, Rockville, MD USA). Approximately 48 h after the second dose administration, peripheral blood was collected for flow cytometric analysis of micronuclei. There was no significant increase in the incidence of micronuclei in the test article-dosed animals compared to the concurrent vehicle control. The vehicle control value was compatible with the historical range of % micronucleated reticulocytes (MnRETs). There was a statistically significant increase in MnRETs in the positive control compared to the concurrent vehicle control. Statistically significant decreases in % RETs were observed in the treatment groups, indicating the compound induced cytotoxicity. Hence, SRP-001 was evaluated as negative (non-clastogenic) under the conditions of this study.

#### Second in vivo study, Comet assay GI tract: SRP-001 is non-DNA damaging

Male Sprague–Dawley rats were orally administered SRP-001 at 500, 1000, or 2000 mg/kg/dose for two consecutive days at a dose volume of 10 mL/kg/dose (Bioreliance, Rockville, MD USA). The vehicle control was 10% Labrasol, 10% DMSO, and 80% PEG400. The positive control for the Comet assay, ethyl methanesulfonate (EMS), was orally administered at 200 mg/kg/dose for two consecutive days at a dose volume of 10 mL/kg/dose. This positive control is an alkylating agent; the negative result for SRP-001 indicates that it did not alkylate DNA to a measurable extent under the conditions of this assay. Animals were euthanized 3–4 h after the last dose administration. The liver and glandular stomach were collected and processed for comet evaluation.

#### Liver

No statistically significant increases in % tail DNA were observed in SRP-001-treated groups. The group mean % tail DNA for the vehicle control was within the 95% control limit of the study historical data range, with one animal (#160) exceeding the individual animal 95% control limit but within the minimum/maximum range. Additionally, three animals (#173, 174, and 175) in the mid-dose (1000 mg/kg/dose) group likewise exceeded the individual animal 95% control limit but were within the minimum/maximum range. The group mean % tail DNA for the positive control was significantly increased when compared to the concurrent group mean % tail DNA for the vehicle control and was compatible with the positive control database for both the study and individual animals.

#### Glandular stomach

No statistically significant increases in % tail DNA were observed in the SRP-001-treated groups. The group mean % tail DNA for the vehicle control was within the 95% control limit of the study historical data range, with one animal (#158) exceeding both the individual animal 95% control limit and minimum/maximum ranges. One animal (#171) in the mid-dose (1000 mg/kg/dose) group and one animal (#180) in the high-dose (2000 mg/kg/dose) group exceeded just the 95% control limit range but were within the minimum/maximum range. One animal (#170) in the mid-dose (1000 mg/kg/dose) group and one animal (#179) in the high-dose (2000 mg/kg/dose) group exceeded both the individual animal 95% control limit and minimum/maximum ranges. The group mean % tail DNA for the positive control was significantly increased when compared to the concurrent group mean % tail DNA for the vehicle control and was compatible with the positive control database for both the study and individual animals. All valid assay criteria were met, and SRP-001 was determined to be negative (non-DNA damaging) in the Comet assay.

### Non-GLP and GLP toxicology

#### Dose-range-finding oral toxicity study with SRP-001 in Sprague–Dawley rats (non-GLP)

As part of a two-phase study, the maximum tolerated dose (MTD) of SRP-001 when administered via gavage once to Sprague–Dawley Rats using an ascending/descending dose design (Calvert Labs/Altasciences, Scott Township, PA USA). A total of 12 male Sprague–Dawley rats (3/dose group), approximately 8–9 weeks old and weighing 245–307 g, were administered single doses of 5000, 1000, 1500, or 2000 mg/kg SRP-001. Mortality/morbidity was performed twice daily and once prior to scheduled sacrifice. Clinical observations were evaluated prior to each dose administration and approximately 1–3 h post-dose, once daily on non-dosing days and additionally as needed. Body weights were recorded for animals in Groups 1–4 prior to randomization/selection, each dose administration, and scheduled sacrifice on day 3. Food consumption was recorded on day 3. Nearly all the animals appeared normal following dosing, and no test article-related effects on body weight or food consumption were observed. All animals appeared unremarkable at gross necropsy. Based on the results of this study, doses for the second phase were selected.

#### A single oral dose toxicity and pharmacokinetic study of SRP-001 in Beagle Dogs (non-GLP)

SRP-001 was prepared into capsules for oral administration. Three experimentally non-naïve Beagle dogs, at least 7 months old and weighing 7.7 to 10 kg prior to treatment initiation, were administered a single dose of 300 mg/kg SRP-001 (Calvert Labs/Altasciences, Scott Township, PA USA). On the day of dosing, animals were observed prior to dose administration, approximately 1–3 h post-dose, and once on day 2. Body weights were recorded for all animals prior to dose administration for the preparation of the capsules. Blood for bioanalytical evaluation was collected at selected timepoints on day 1. Following the completion of the study, surviving animals were not euthanized. They were returned to the Calvert colony for an appropriate washout period before possible use in a subsequent study. The dose was well tolerated, and all animals appeared normal following dosing. Plasma samples were collected at seven different time points post-dose. Bioanalytical analysis revealed poor bioavailability was achieved following dosing. Capsule dosing, while well tolerated in dogs at 300 mg/kg, was not considered suitable for future studies.

#### A dose-range-finding and 7-day repeat-dose oral toxicity study of SRP-001 in Beagle dogs (Non-GLP)

Two beagle dogs (1/sex) were administered 30, 100, 250, or 500 mg/kg SRP-001. Animals were observed for clinical signs of toxicity or effect prior to dose administration and approximately 1–3 h post-dose, additionally as needed, and daily on non-dosing days (Calvert Labs/Altasciences, Scott Township, PA USA). After a period of at least 2 d (minimum of 44 h) following the previous dose of test article, additional dose levels of SRP-001, as indicated above, were administered by oral gavage to the same 2 dogs (1 male and 1 female). Each dose was based on the dog’s most recent body weight. Mortality/morbidity was observed twice daily and once prior to scheduled sacrifice. Body weights were recorded prior to randomization/selection, prior to each dose administration, and prior to scheduled sacrifice. At scheduled sacrifice, a gross necropsy was performed, and the tissues were appropriately discarded. Loose feces and emesis were observed following most doses primarily observed within 4 h post dosing at higher doses, however, no dose-dependent trend was observed. No test article-related effects on body weight or food consumption were observed. At gross necropsy, Phase I animals had some red discoloration in the GI tract, as observed in the duodenum, jejunum, ileum, and rectum. Doses for Phase II of the study were set based on Phase I results. The actual dose amounts selected for Phase II of the study were 0, 30, 100, or 300 mg/kg/d of SRP-001. These doses were administered once daily for seven consecutive days to 1 Beagle dog/sex/dose.

#### A dose-range-finding oral toxicity study with SRP-001 in Sprague–Dawley rats (Non-GLP)

As part of a two-phase study, repeat-dose toxicity and toxicokinetics of SRP-001, when administered via oral gavage once daily to Sprague–Dawley rats for 5 d, was done (Calvert Labs/Altasciences, Scott Township, PA USA). The doses selected for Phase II were based on the results of Phase I. SRP-001 was administered to 20 naïve rats (5/group in Groups 5–8) once daily for 5 days via oral gavage for toxicology evaluation and once to 18 naïve rats (6/group in Groups 9–11) for toxicokinetic evaluation.

Mortality/morbidity was performed twice daily and once prior to sacrifice. Clinical observations were evaluated prior to each dose administration and approximately 1–3 h post-dose for animals in Groups 5–8. Body weights were recorded prior to randomization/selection, prior to dose administration on day 1 for animals in Groups 5–11, and prior to dose administration on day 5 for Groups 5–8. Food consumption was recorded for animals from Groups 5–8 on day 5. Blood for evaluation of hematology, coagulation and clinical chemistry and urine for urinalysis was collected from animals in Groups 5–8 on day 6. All animals in Groups 5–8 were sacrificed on day 5. Selected tissues were harvested at necropsy, selected organs weighed, and selected tissues preserved. Blood for toxicokinetic evaluation was collected from animals in Groups 9–11 at selected timepoints on day 1 to ensure comprehensive data capture. These timepoints include 0 h (pre-dose) and post-dose intervals of 1, 2, 4, 8, and 24 h on both Day 1 and Day 28 of the study. This schedule allows for a robust assessment of SRP-001's pharmacokinetic profile, including absorption, distribution, metabolism, and excretion and is in line with current FDA-guidelines for GLP studies.

Clinical signs included soft feces, brown staining of the anogenital area, and a few instances of red staining of the muzzle. These signs were observed in all groups and are likely related to the vehicle (10% Labrasol: 10% DMSO: 80% PEG400) used. Test article-related increases in absolute and relative liver weights and increased cholesterol levels were observed. Dark discoloration of the liver was observed in the majority of control animals and all experimental animals at gross necropsy. No SRP-001-related differences in body weight, food consumption, hematology, or coagulation parameters were observed. AUC and Cmax levels increased in a dose-dependent manner. Half-lives were 6.19 and 6.86 h at mid and high doses, respectively. Tmax ranged from 3 h at the low dose to 4.5 h at mid and high doses. Based on these observations, SRP-001 was considered to be tolerated at 1200 mg/kg when administered once daily for 5 consecutive days.

#### A 28-day oral toxicity study of SRP-001 in Sprague–Dawley rats with 14-day recovery (GLP study)

We next set out to determine the toxicity and toxicokinetics of SRP-001 when administered via oral gavage once daily to Sprague–Dawley Rats for 28 d (Calvert Labs/Altasciences, Scott Township, PA USA). The study also assessed the reversibility of any toxicity observed with a 14-d recovery period. One hundred thirty-six experimentally naïve Sprague–Dawley rats (68 males and 68 females), 8 weeks old and weighing 167–295 g for males and females at the outset of the study, were assigned to toxicology treatment groups (Groups 1–4) or toxicokinetic groups (Groups 5–7). For toxicology groups, 10 animals/sex/dose were administered 0, 300, 900, or 1500 mg/kg/d SRP-001. An additional 5 animals/sex/dose in Groups 1 and 4 were targeted for a 14-d recovery period and euthanized on day 43. For toxicokinetic groups, 6 animals/sex/dose were administered 300, 900, or 1500 mg/kg/d SRP-001. Mortality/morbidity was performed twice daily and once prior to scheduled sacrifice. Animals in Groups 1–4 were observed prior to each dose administration and 1–3 h post-dose. Animals in Groups 1–4 were also observed once daily on non-dosing days and once prior to scheduled sacrifice. Body weights were recorded prior to randomization/selection, prior to dose administration on day 1, and weekly thereafter. Food consumption was determined weekly for animals in Groups 1–4. Ophthalmology examinations were performed before treatment initiation and during the last week of dosing for animals in Groups 1–4. The functional observational battery was assessed for 5 animals/sex/group in Groups 1–4 once prior to dosing and at 1–2 h following dose administration on day 1. Blood for evaluation of hematology, coagulation, and clinical chemistry and urine for urinalysis was collected from animals in Groups 1–4 on day 29 or day 43. Blood for toxicokinetic evaluation was collected from animals in Groups 5–7 at selected timepoints on days 1 and 28. All surviving animals in Groups 1–4 were sacrificed on day 29 or day 43. Selected tissues were harvested at necropsy, selected organs weighed, and selected tissues evaluated microscopically.

Following dosing, the reversibility of any toxicity observed was assessed with a 14-d recovery period of animals receiving 0 or 1500 mg/kg of SRP-001. SRP-001-related clinical signs included red staining around the forepaw, forelimbs, mouth, muzzle, and nose and brown-staining in the anogenital area. To some extent, these clinical observations appeared to be vehicle-related; however, they were observed at an increased frequency and duration correlating with increasing dose levels. The majority of clinical signs were observed during the first 3 weeks of dosing, and by day 28, the majority of the animals appeared normal. Hematology and serum chemistry analysis on day 29 identified non-adverse SRP-001-related increases in globulin, albumin, and total protein and decreases in aspartate aminotransferase, alkaline phosphatase, and mean corpuscular hemoglobin concentration. These differences remained within or close to the historically normal ranges. Statistically significant differences in total protein, globulin, and mean corpuscular hemoglobin remained present in males after the recovery period, although at a smaller magnitude. SRP-001-related microscopic findings were limited to the liver and included minimal to mild centrilobular hepatocellular hypertrophy in both sexes at ≥ 300 mg/kg/d. This finding correlated with statistically significant increases in liver weights in both sexes. Hepatocellular hypertrophy was considered to be an adaptive response and non-adverse due to limited severity, lack of histological evidence suggestive of structural damage (such as a concurrent dose-dependent increase in hepatocellular necrosis or inflammation), and lack of dose-dependent and biologically significant increases in clinical chemistry parameters suggestive of hepatobiliary damage.

There were no SRP-001-related effects on body weight, food consumption, functional observation battery, ophthalmological findings, coagulation parameters, or urinalysis parameters. Exposure to SRP-001 following oral gavage dosing at 300, 900, and 1500 mg/kg SRP-001 was dose-dependent, increasing with escalating doses following a single dose. Exposure to SRP-001 following 28 consecutive days of oral gavage doses at 300, 900, and 1500 mg/kg/d SRP-001 was dose-dependent but did not increase as much as seen on day 1. Based on the parameters observed, the no-adverse effect level (NOAEL) of SRP-001, when administered once daily for 28 d to Sprague–Dawley rats, was 1500 mg/kg.

Cardiotoxicity is also absent with SRP-001. This stems from no cardiovascular effects in 28-day GLP studies in both beagle dogs or rats and in a telemetered safety pharmacology study in dogs. It did not result in a relevant signal in the in vitro hERG assay in human embryonic kidney cells at dosages where acetaminophen causes significant changes in these markers is absent with SRP-001. SRP-001 was a weak blocker of hERG current with the highest concentration tested (100 μM) blocking by 21.4 ± 3.9% (Supplementary Fig. 1d). Oral SRP-001 administration at doses of 220, 440, and 660 mg/kg did not induce any significant effects on respiratory rate, tidal volume, or minute volume in conscious male Sprague–Dawley rats. No apparent neuropharmacological or clinical signs were observed through 24 h post-dose in any rats receiving either vehicle at 10 mL/kg or SRP-001at 220, 440, or 660 mg/kg, except for a lower body temperature in the high dose group (660 mg/kg). Oral administration of SRP-001 at doses of 30, 90, and 150 mg/kg in conscious telemetered beagle dogs did not induce any biologically relevant effects on blood pressure, heart rate, ECG morphology, ECG measurements, or body temperature.

#### A two-day oral dose toxicity and pharmacokinetic study of SRP-001 in Beagle Dogs (Non-GLP)

SRP-001 was supplied by Olon Ricerca Bioscience as an off-white powder and then prepared as dosing suspensions for oral administration via gavage (Calvert Labs/Altasciences, Scott Township, PA USA). Six experimentally non-naïve male Beagle dogs (3/dose), at least 7 months old and weighing 7.2 to 10.2 kg prior to treatment initiation, were administered 100 or 900 mg/kg/d SRP-001. Animals were dosed three times daily (3–5 h between doses) for 2 d. Mortality/morbidity was performed twice daily. On the day of dosing, animals were observed prior to each dose administration and approximately 1–3 h after each dose. Animals were also observed once on day 3. Body weights were recorded for all animals prior to the first dose administration on days 1 and 3, and food consumption was recorded daily. Blood for bioanalytical evaluation was collected at selected timepoints on day 1. Following the completion of the study, surviving animals were not euthanized and were returned to the Calvert colony.

Nearly all animals had emesis within 1 -2 h following each dose administration. Other clinical signs observed included loose or watery feces and salivation. Nearly all animals appeared normal prior to receiving their next dose. Body weight loss was also observed; however, no animal lost more than 4% of their body weight. Body weight loss correlated with decreased food consumption on day 3. Bioanalytical analysis of plasma confirmed SRP-001 exposure in all animals. Overall, SRP-001 was tolerated when administered 3 times daily for 2 d at 900 mg/kg/d, and plasma exposure was observed.

#### A dose-range-finding and 7-day repeat-dose oral toxicity study of SRP-001 in Beagle dogs (Non-GLP)

In Phase II of this study, 0, 30, 100, or 300 mg/kg/d of SRP-001 was administered once daily for seven consecutive days to 1 Beagle dog/sex/dose (Calvert Labs/Altasciences, Scott Township, PA USA). Mortality/morbidity was observed twice daily and once prior to scheduled sacrifice. Clinical observations were evaluated prior to each dose administration and at approximately 1–3 h post-dose and once prior to scheduled sacrifice on day 8. Body weights were recorded on days 1 and 7, and a fasted body weight was recorded prior to scheduled sacrifice on day 8. Food consumption was recorded daily. Blood for evaluation of hematology, coagulation, and clinical chemistry parameters and urine for urinalysis was collected from all animals prior to treatment initiation and from all Phase II animals prior to scheduled sacrifice on day 8. Blood for toxicokinetic evaluation was collected at selected time points on days 1 and 7. Selected tissues were harvested and weighed at necropsy. No microscopic evaluations were performed. Clinical signs observed included loose or soft feces and emesis. While soft or loose feces were observed at similar frequencies in all animals, emesis was observed at a higher incidence and greater volume in animals that received higher dose levels. The clinical observations corroborated with findings of red discoloration in the duodenum of the control group female, the jejunum of the low-dose male, and the colon of both high-dose dogs. No SRP-001-related differences in body weight, food consumption, hematology, clinical chemistry, or coagulation parameters were observed.

AUC and Cmax levels increased in a dose-dependent manner, with day 1 levels higher than those from day 7. The reason for this is unclear; however, it may be due to a low sample size (n = 2 per dose group). Half-lives ranged from approximately 1 to 3.5 h and increased with dose and time. Tmax ranged from 0.5 to 1.5 h. Based on these observations, SRP-001 was considered to be tolerated at 300 mg/kg when administered once daily for 7 consecutive days. Our toxicokinetic analysis indicates elevated systemic exposures to SRP-001 on Day 1 relative to Day 7. This trend is corroborated by a decline in both AUC and Cmax values on Day 28, aligning with our prior 28-day rat toxicology study that reported analogous reductions in systemic exposures at daily dosages of ≥ 1500 mg/kg/day. This is further supported by human pharmacokinetic data from the Phase 1 MAD cohort, which also demonstrated a decrease in SRP-001's PK parameters on Day 5 versus Day 1. A plausible mechanistic explanation for this observed decrease is the induction of metabolic enzymes, likely involving UGT and potentially CYP3A enzymes, as elaborated above and substantiated by previous research^30^. While emesis was observed, its frequency did not increase over time, making it an unlikely factor in the reduced systemic exposure on Day 28. Moreover, no emesis was reported in the Phase 1 MAD cohort, which also showed a PK parameter decrease on Day 5 compared to Day 1. The NOAEL exposure parameters align well with the highest SAD dose in the Phase 1 trial, reinforcing the drug's safety profile. The observed PK decrease is not attributed to emesis but likely to enzymatic induction and will be the subject for future investigation.

#### A 28-day oral toxicity study of SRP-001 in Beagle dogs with a 14-day recovery (GLP study)

SRP-001 was supplied by Olon Ricerca Bioscience as an off-white powder. The test article was then prepared into dosing suspensions for oral administration via gavage (Calvert Labs/Altasciences, Scott Township, PA USA). Thirty-two experimentally naïve Beagle dogs (16 males and 16 females), 8–9 months old and weighing 6.7–10.5 kg for males and females at the outset of the study, were administered 0, 165, 330, or 495 mg/kg/d. Animals were dosed three times daily (3–5 h between doses) for 28 consecutive days. Prior to this dosing schedule, the dosing was initiated with once daily dosing at 0, 100, 300, or 900 mg/kg at a dose volume of 5 to 10 ml/kg. Due to adverse clinical signs noted following dosing of all groups at a dose volume of 10 ml/kg, dosing of the animals was suspended on day 2 for males and day 1 for females. The study was restarted with T.I.D. dosing in lower dose volume to reduce vehicle-related toxicity.

In-life data from the initial start of the study using once daily dosing were not included in the report but are maintained in the study file. Following the restart of the study, the study was conducted as follows. Mortality/morbidity was performed twice daily and once prior to the scheduled sacrifice. Animals were observed prior to each dose administration and 1–3 h after each dose administration. Animals were also observed once daily on non-dosing days and once prior to scheduled sacrifice. Body weights were recorded prior to randomization/selection, prior to the first dose administration on day 1 and weekly thereafter. Food consumption was determined daily. Ophthalmology examinations and electrocardiograms were performed before treatment initiation and during the last week of dosing. Blood for evaluation of hematology, coagulation and clinical chemistry and urine for urinalysis was collected prior to treatment initiation and on days 29 and 43. Blood for toxicokinetic evaluation was collected at selected timepoints on days 1 and 28. All surviving animals were sacrificed on day 29 or day 43. Selected tissues were harvested at necropsy, selected organs weighed, and selected tissues evaluated microscopically. SRP-001-related clinical signs included emesis, loose feces, and salivation. While emesis and loose feces were observed in all dose groups, including the control, they were observed at a higher frequency and severity with increasing dose levels.

Assessment of hematology parameters identified SRP-001-related decreases in red blood cells, hemoglobin, hematocrit, and mean corpuscular hemoglobin concentration and increases in mean corpuscular volume and absolute and relative reticulocytes. These changes correlated with increased erythroid cellularity of the bone marrow and extramedullary hematopoiesis of the spleen and liver, indicative of regenerative anemia. Due to the limited magnitude of the changes in hematology parameters and the corresponding changes in reticulocytes, this finding was not considered adverse.

Assessment of clinical chemistry parameters indicated SRP-001-related increases in triglycerides and alkaline phosphatase and decreases in cholesterol. Increases in alkaline phosphatase correlated with bile accumulation in the liver of Group-4 males. This finding was indicative of the biliary system starting to be overwhelmed and was considered adverse. Other microscopic findings of the liver included Kupffer cell pigment, Kupffer cell erythrophagocytosis, and Kupffer cell hypertrophy/hyperplasia. The Kupffer cell changes were thought to be, at least in part, due to phagocytosis of erythrocytes resulting in the accumulation of cytoplasmic pigment and increased demand for Kupffer cells resulting in hypertrophy/hyperplasia. These microscopic changes correlated with increased liver organ weights. Microscopic evaluation also identified thyroid gland follicular cell hypertrophy.

No SRP-001-related changes were observed in body weights, food consumption, ophthalmology examination, electrocardiology examination, coagulation parameters, or urinalysis parameters. Analysis of dogs following a 14-d recovery period indicated that nearly all SRP-001-related changes observed on day 29 were reversible. Only microscopic changes in Kupffer cell pigment and erythrophagocytosis and minimal increased erythroid cellularity of the bone marrow were still present in Group-4 animals following the recovery period. Exposure to SRP-001 was dose-dependent and generally dose-proportional to greater than dose-proportional from 165 to 330 mg/kg/d and less than dose-proportional from 330 to 495 mg/kg/d. Exposure to SRP-001 following 28 consecutive days of oral dosing was 2 to 5 times less than exposure observed on day 1. Based on the clinical signs, microscopic changes, and clinical pathology changes, the no-adverse-effect level for SRP-001 was 330 mg/kg/d when given T.I.D. for 28 d.

### Oral nanosuspension formulation for human use and determination of the optimal formulation stability

The drug therapeutic candidate is manufactured in a two-step process (Latitude Pharmaceuticals, Inc., San Diego, CA, USA). First, SRP-001 is jet milled. Second, the milled API is dispersed in a solution comprising 1% hydroxypropyl cellulose (1% HPC) and sterile water for injection and subjected to wet milling. The target particle size is approximately D_90_ 0.15 μm. Hence, using nanomilling and jet-milling techniques, we identified the optimal solvent in which to dissolve to develop SPR-001 as an oral nanoparticle suspension containing 100 mg/mL SRP-001 in preserved aqueous 1% HPC (Supplementary Fig. 20). An oral nanoparticle suspension containing 100 mg/mL SRP-001 in aqueous 1% HPC preserved with 0.1% sodium benzoate was manufactured for use in nonclinical studies as Lot LPI2021028 and stored at 5 °C ± 3 °C. All specifications were met at time 0, 1 month, and 3 months at 5 °C and 25 °C/60% RH (relative humidity). The same formulation as above was manufactured under cGMP for use in clinical studies as Lot LPI2021031 and stored at 5 °C ± 3 °C. All specifications were met. This lot has been placed on stability at 5 °C, 25 °C/60%RH, and 40 °C/75%RH. Finally, an appearance-matched placebo consisting of 2% microcrystalline cellulose in aqueous 1% HPC preserved with 0.1% sodium benzoate and 0.05% EDTA disodium dihydrate was manufactured under cGMP for use in clinical studies. The above oral suspensions were manufactured by Latitude Pharmaceuticals Inc. (San Diego, CA, USA). Furthermore, the active pharmaceutical ingredient (API, SRP-001) manufacturer, Olon Ricerca Biosciences, demonstrated that the API is stable for at least 9 months at 25 °C/60%RH and 6 months at 40 °C/75%RH.

### Human studies

A two-part randomized, double-blind, placebo-controlled Phase 1 trial was conducted to assess the safety, tolerability, PK and PD of SRP-001 ascending doses (Quotient Sciences, Miami, FL, USA). The study was conducted in accordance with the International Council for Harmonisation of Technical Requirements for Pharmaceuticals for Human Use (ICH), Guideline for GCP: Consolidated Guidance (E6), and applicable regulatory requirements(s) including clinical research guidelines established by the Basic Principles defined in the U.S. 21 CFR Parts 50, 56, and 312 and the principles enunciated in the Declaration of Helsinki (revised version Fortaleza 2013). The clinical protocol and informed consent form were reviewed and approved by an Institutional Review Board (IRB; Advarra IRB, 6100 Merriweather Drive, Suite 600, Columbia, MD 21,044; Quotient Protocol No. SRP-101 and Quotient Sciences Study No. QSC205130; Quotient Sciences Miami, 3898 NW 7th Street, Miami, FL 33,126.). The study comprises a SAD (Part 1) assessment that includes a food effect assessment that contributes data to inform a subsequent MAD (Part 2) dose-ranging study. Safety measurements were collected throughout the study for all subjects. Blood samples were collected to determine the PK parameters of **SRP-001** (ClinicalTrials.gov identifier: NCT05484414) (02/08/2022).

#### Inclusion criteria

The normal healthy volunteer (NHV) subjects are healthy adult male or female subjects, 18–55 years of age inclusive, with a body mass index of between 18.0 and 32.0 kg/m^2^ (inclusive) as measured at screening or, if outside the range, considered not clinically significant by the Investigator, and weighs within ± 15% of the desired weight at screening, according to the 1993 Metropolitan Life Insurance Table. Females must not be pregnant or breastfeeding. The subjects must be capable of understanding the Informed Consent Form and agreeing to comply with the requirements of the study during its duration. The subjects are to be in good health as determined by medical history, physical examination, clinical laboratory test results, and 12-lead ECG. Females must be of non-childbearing potential (defined as surgically sterile (i.e. had a bilateral tubal ligation, bilateral salpingectomy, hysterectomy, or bilateral oophorectomy) or post-menopausal for at least 1 year before the first dose of study medication) or agree to use an acceptable form of birth control from screening until 31 days after last SRP-001 administration. Male subjects must either be surgically sterile (vasectomy at least 3 months prior to first dose) or agree to the use of a birth control method such as a condom with spermicide from screening until 91 days after last SRP-001 administration. All subjects must also be willing and able to remain in the research center for the entire duration of the confinement period and return for the outpatient visits; and, have vital signs (measured after the subject has been in a supine position for a minimum of 5 min) at screening within the following ranges: heart rate: 40–100 bpm; systolic blood pressure (BP): 90–145 mmHg; diastolic BP: 50–95 mmHg. Out-of-range vital signs may be repeated once.

#### Exclusion criteria

NHVs were excluded from study participation for any of the following. A history or presence of clinically significant cardiovascular, pulmonary, hepatic, renal, hematologic, GI, endocrine, immunologic, dermatologic, neurologic, oncologic, or psychiatric disease or any other condition that, in the opinion of the Investigator, would jeopardize the safety of the subject or the validity of the study results (a clinically significant illness within 30 days preceding the screening visit). If he or she had been on a significantly abnormal diet during the 4 weeks preceding the first dose of study medication. Subjects who have received any investigational product in a clinical research study within 5 half-lives or within 30 days prior to first dose. However, in no event shall the time between the last receipt of investigational product and the first dose be less than 30 days. Any NHVs who have previously been administered SRP-001 in this study. Subjects who are taking, or have taken, any prescribed drug (other than hormone replacement therapy/hormonal contraception) in the 14 days before study medication, or OTC drug or herbal remedies in the 72 h before study medication. COVID-19 vaccines are accepted concomitant medications. NHVs were also excluded if they had been treated with any known drugs that are moderate or strong inhibitors/inducers of cytochrome P450 (CYP) enzymes (e.g., barbiturates, phenothiazines, cimetidine, carbamazepine) within 30 days before the first dose of study medication, and that, in the Investigator’s judgment, may impact subject safety or the validity of the study results; if they had a history of hypersensitivity (has developed an allergic reaction) to acetaminophen or similar chemical entities; or presence or history of clinically significant allergy requiring treatment, as judged by the Investigator. The NHV’s hemoglobin concentration and hematocrit should be within 5% of normal before participating in the study. It is recommended that blood/plasma donations not be made for at least 30 days after discharge from the study. Current smokers and those who have smoked within the last 12 months were also excluded, as anyone with a confirmed positive urine cotinine test at screening or admission; current users of e-cigarettes and nicotine replacement products and those who have used these products within the last 12 months; consumption of beverages or foods that contain alcohol, grapefruit, poppy seeds, broccoli, Brussels sprouts, pomegranate, star fruit, char-grilled meat, or caffeine/xanthine from 48 h before the first dose of study medication until discharge from the study. NHVs were instructed not to consume any of the above products. Also excluded is an NHV with a confirmed positive alcohol urine test at screening or admission; a female with a positive pregnancy test result; or a positive urine screen for drugs of abuse (amphetamines, barbiturates, benzodiazepines, cocaine, cannabinoids, or opiates); a positive test for hepatitis B surface antigen (HBsAg), hepatitis C antibody (HCV Ab), or human immunodeficiency virus (HIV) at screening or has been previously treated for hepatitis B, hepatitis C, or HIV infection. Anyone with the presence of active infection, mucositis, cold sores, aphthous ulcers, vesicles, viral lesions, local irritation/inflammation, or periodontal disease of the oral cavity was also excluded, as well as NHVs with a known glucose-6-phosphate-dehydrogenase (G6PD) deficiency.

#### Subject randomization scheme

NHVs were assigned a three-digit screening number after informed consent was obtained (e.g., 001, 002, 003, etc.). Four-digit subject numbers were allocated on the morning of dosing according to the code 1001 to 1020 for males and 1021 to 1040 for females using the lowest number available. Replacement subjects were allocated subject numbers 9001 to 9020 for males and 9021 to 9040 for females, where the last three digits are the same as those of the original subject (e.g. if Subject 1005 withdraws, the replacement will have the Subject Number 9005 and will receive the same regimen as Subject 1005. In addition, for the 900 mg fed cohort, subjects received the same regimen as the withdrawn subject in both the fasted and fed states.

#### Blinding pharmacy staff

An unblinded pharmacy staff was required at the Clinical Site to comply with the study’s randomization and blinding requirements. At the clinical research unit, prior to study initiation, the Principal Investigator was responsible for designating a qualified pharmacy staff to serve as the unblinded pharmacy staff in the study. Unblinded pharmacy staff could dose subjects but could not participate in any subject assessments. Throughout the study, the designated unblinded pharmacy staff was responsible for all drug accountability issues, including preparing, labeling, dispensing, and dosing study drug according to the randomization code provided, yet remain independent of all subject assessments. The pharmacy staff followed the standard operating procedures and Work Instructions related to pharmacy services and protocol-specific requirements. Randomization codes were provided to the unblinded pharmacy staff. Confirmation of receipt of the randomization code was required by the Sponsor. The Principal Investigator was ultimately responsible for ensuring that the integrity of the blind is maintained throughout the study at the site and required to notify the Safety Review Committee in the event of any breaking of the blind for any reason. *Clinical Research Staff –* All observers who evaluate any reported AE, laboratory abnormalities, ECGs, and changes in the ECGs were blinded as to what treatment each subject is assigned. *Study Subjects –* All subjects were blinded as to which treatment they received and dose. *Data Sciences Staff* – The unblinded statistician was not involved in any decisions relating to populations for analysis prior to unblinding. Interim PK parameter estimations were performed using bioanalytical data applied with subject aliases to maintain the study blinded. Further, the Data Sciences department did not have access to the randomization schedule before database lock and unblinding. *Pharmacovigilance* – A copy of the randomization schedule was made available to the pharmacovigilance provider for analysis of pharmacovigilance. *Bioanalytical Laboratory* – All samples were sent to the bioanalytical laboratory for analysis. The bioanalytical laboratory was unblinded, a copy of the randomization schedule provided, and only ran the analysis on active treatment subjects. *Pharmacokinetic Analysis* – PK analysis was completed only on the active treatment subjects and blinded by subject aliases for interim assessments.

#### Check-in procedures

At each check-in, all subjects were evaluated to confirm they continue to meet all the inclusion criteria and none of the exclusion criteria. General risk mitigation against COVID-19 was implemented in accordance with Quotient Sciences’ monitoring and prevention control measures. A urine sample was collected from all subjects at each study check-in to screen for drugs of abuse and alcohol. If at any time an alcohol or drug test is positive, the subject is discontinued from study participation. Female subjects of childbearing potential and male subjects will be asked to confirm that they still adhere to the contraceptive criteria. A serum pregnancy test was performed on all female subjects at each check-in using the clinical chemistry blood sample; this test had to be negative for the subject to continue study participation. Blood and urine samples were also collected at admission for clinical laboratory assessments.

#### Confinement

NHVs were admitted to the research center in the morning of the day before study drug administration (Day -1) to ensure a minimum 10-h fast before dose administration for the fasted regimens. Subjects enrolled in the study had remained in the research center until the completion of the study procedures scheduled for 48 h after dose administration (Day 3), and they returned to the research center for 3 outpatient visits (Days 4, 5, and 6).

#### Follow-up

A follow-up phone call took place between Days 7 and 10 (+ 2 days) to ensure the ongoing well-being of the subjects. If a subject reported any AEs which could present a cause for concern, they were required to attend the clinical unit for a further follow-up assessment (as an unscheduled visit) and were followed up until the AE had resolved. Completion of the last follow-up call or unscheduled follow-up visit was considered the end of the study.

#### Fasting/meals/beverages

An optional meal or snack was served the evening of check-in (Day -1). NHVs were allowed water up to 1 h before the scheduled dosing time and were provided with 240 mL of water at 1 h post-dose. Water was allowed ad libitum after 1 h post-dose. Decaffeinated fluids were allowed ad libitum from lunchtime on the day of dosing. Decaffeinated fluids were allowed ad libitum from lunchtime on the day of dosing until 1 h before and then from 1 h after the afternoon and evening dose.

#### Fasted and fed dosing

NHVs were dosed in the fasted state. The calorie/fat content of meals was not required to be controlled. Subjects were provided with a standardized menu. Subjects were provided with a light snack and then fasted from all food and drink (except water) for a minimum of 10 h on the day prior to dosing until approximately 4 h post-dose, at which time lunch was provided. An evening meal was provided at approximately 10 h post-dose/post-morning dose and an evening snack at approximately 14 h post-morning dose. Food effect was studied in the cohort receiving 900 mg SRP-001 in the fed state. A standard high-fat breakfast was given 30 min before dosing. On Day -1, these NHVs were provided with a light snack and fasted from all food and drink (except water) until the following morning, when they were provided with a standard high-fat breakfast. The breakfast had to be consumed over a maximum period of 25 min, with dosing occurring 30 min after the start of breakfast. Subjects were encouraged to eat their meal evenly over the 25 min period. It was acknowledged that some subjects took less time to eat, but dosing still occurred 30 min after the start of breakfast. Subjects had to consume at least 90% to be eligible for dosing. The start and stop time and percentage of the breakfast consumed were recorded in the source. Acceptable deviations for the pre-dose meal from the nominal time point were: (1) Pre-dose meal was provided within ± 5 min of the nominal time point, (2) Lunch was provided at approximately 4 h post-dose, (3) an evening meal at approximately 10 h post-dose, and (4) an evening snack at approximately 14 h post-dose. On subsequent days, meals were provided at appropriate times.

#### Safety – Holter (continuous ECG) monitoring

NHVs also underwent continuous ECG Holter monitoring from at least 1 h prior to dosing until 24 h post-dose, and resulting measurements were monitored consistently. All ECGs were collected electronically using a Mortara H12 + Holter monitor. Specific procedures for ECG Holter recording and extractions were provided to the Investigator by the Central ECG Laboratory, Banook Group. On Day 1, continuous Holter recording commenced at least 1 h prior to dosing until 24 h post-dose. Subjects were required to rest in the supine position with no external stimuli (e.g. music or television) for 15 min prior to the nominal time point where an ECG extraction was scheduled. This extraction was taken by the third-party Holter provider at the planned time, and therefore, no other procedures were performed within the resting period. Where one period of ECG rest ran into the rest period for the next ECG extraction, the minimum resting period before ECG extraction did not need to be started again except in cases where the rest was disturbed (e.g. subjects had to get up for a comfort break after the preceding extraction). Subjects were allowed to move more freely outside of the primary ECG rest periods. Comfort breaks for hygienic purposes were allowed each morning well in advance of the required supine/ECG extraction points. Where any scheduled rest times were missed, where there was less than a 10-min rest period, or where the final 5 min of a rest period was interrupted (for example, by subject movement or loss of leads), a protocol deviation was recorded, and the Holter provider was informed. Loss of leads or interruption of the recording outside of the scheduled rest times was not considered a protocol deviation but was recorded in the source. The acceptable deviations for ECG rest periods from the nominal time point were pre-dose and post-dose ECG resting periods ending within ± 15 min of the nominal time point. ECGs were collected electronically and over-read by cardiologists at the Central ECG Laboratory, Banook Group. The Central ECG Laboratory over-read was used for data analysis and report writing purposes.

#### Adverse event

An AE was defined as any untoward medical occurrence associated with the use of a drug in humans, whether or not considered drug-related. An AE could be any unfavorable and unintended sign (e.g. an abnormal laboratory finding), symptom, or disease temporally associated with the use of a drug, without judgment to causality. An AE could arise from any use of the drug (e.g. off-label, use in combinations with another drug) and from any route of administration, formulation, or dose, including an overdose.

#### Recording adverse events

AEs were recorded from the time of providing written informed consent until 30 days after the last dose of study drug. During each study visit, the subject was questioned directly regarding the occurrence of any adverse medical event according to the schedule in eCRF. All AEs, whether ascribed to study procedures or not, were documented immediately in the subject’s eCRF. This included the date and time of onset, a description of the AE, severity, seriousness, duration, actions taken, outcome, and an Investigator’s current opinion on the relationship between the study drug and the event. A diagnosis and final opinion on the relationship between the study drug and the event were provided at the end of the study by the Quotient Sciences Principal Investigator. Any subject who withdrew from the study due to an AE was followed up until the outcome was determined and written reports were provided by the Investigator.

#### Life-threatening adverse event/life-threatening suspected adverse reaction

A life-threatening AE/life-threatening suspected adverse reaction, in the view of either the Investigator or Sponsor, placed the patient or subject at immediate risk of death. It did not include an adverse reaction that, had it occurred in a more severe form, might have caused death. None of these occurred in the study. Any life-threatening AE was reported and managed as an SAE.

#### Serious adverse event/serious suspected adverse reaction

A SAE or serious suspected adverse reaction, in the view of the Principal Investigator, resulted in any of the following outcomes: Death, a life-threatening AE, inpatient hospitalization or prolongation of existing hospitalization, a persistent or significant incapacity or substantial disruption of the ability to conduct normal life functions, or a congenital anomaly/birth defect. Important medical events that might not result in death, be life-threatening, or require hospitalization could be considered serious when, based upon appropriate medical judgment, they might jeopardize the patient or subject and might require medical or surgical intervention to prevent one of the outcomes listed in this definition.

#### Unexpected adverse event/unexpected suspected adverse reaction

An unexpected AE/unexpected suspected adverse reaction was an AE or suspected adverse reaction that was not listed in the FDA Investigator Brochure (IB) or was not listed at the specificity or severity that had been observed; or, was not consistent with the risk information described in the general investigational plan or elsewhere in the current application, as amended.

#### Grading of adverse events

The severity of AEs was assessed as follows: *Mild* – An AE that was easily tolerated by the subject, caused minimal discomfort, and did not interfere with everyday activities. *Moderate* – An AE that was sufficiently discomforting to interfere with normal everyday activities; intervention might have been needed. *Severe* – An AE that prevented normal everyday activities; treatment or other intervention was usually needed. *Relationship to Study Treatment* – The relationship between the AE and the investigational product was determined by the Principal Investigator or Sub-Investigator on the basis of their clinical judgment and the following definitions: *Related*: The AE followed a reasonable temporal sequence from the study product administration and could not be reasonably explained by the subject's clinical state or other factors (e.g. disease under study, concurrent diseases, or concomitant medications). The AE followed a reasonable temporal sequence from the study product administration and represented a known reaction to the drug under study or other drugs in its class or was predicted by the known pharmacological properties of the drug. The AE resolved with discontinuation of the investigational product and/or recurred with rechallenge, if applicable. *Not Related*: The AE did not follow a reasonable temporal sequence from study product administration or could be reasonably explained by the subject’s clinical state or other factors (e.g. disease under study, concurrent diseases, and concomitant medications).

#### SRP-001 administration and PK/PD determination

Following a single administration of 300–2000 mg SRP-001 oral suspension in the fasted state (and in cohort 4, 900 mg in the fed state), plasma concentrations of SRP-001 were observed from the first sampling timepoint of 1 h post-dose for all subjects. Peak concentrations (Tmax) were also reached at 1 h post-dose for all subjects, after which concentrations declined in a mono- or bi-phasic manner and remained quantifiable up to between 4 to 12 h postdose, giving rise to a geometric mean T1/2 of 2.38 h. Maximum and overall plasma exposure of SRP-001 based on geometric mean (geometric CV%) Cmax, AUC(0–48) and AUC(0-inf) were 665 ng/mL (55.5%), 1630 ng.h/mL (37.6%) and 1630 ng.h/mL (37.4%) respectively.

Following a single administration of 600 mg SRP-001 oral suspension in the fasted state, plasma concentrations of SRP-001 were observed from the first sampling timepoint of 1 h post-dose for all subjects. Peak concentrations (Tmax) were reached between 1 and 2 h post-dose for all subjects, after which concentrations declined in a mono- or bi-phasic manner and remained quantifiable up to between 8.

to 24 h post-dose, giving rise to a geometric mean T1/2 of 5.04 h. Maximum and overall plasma exposure of SRP-3D based on geometric mean (geometric CV%) Cmax, AUC(0–24) and AUC(0-inf) were 1740 ng/mL (48.7%), 5660 ng.h/mL (25.3%) and 5840 ng.h/mL (29.5%), respectively. Increasing the dose from 300 to 600 mg also increased geometric mean Cmax and AUC(0–24) values by 2.6- and 3.5-fold.

Following a single administration of 900 mg SRP-001 oral suspension in the fasted state, plasma concentrations of SRP-001 were observed from the first sampling time-point of 1 h post-dose for all subjects. Peak concentrations (Tmax) were reached at 1 h post-dose for all subjects, after which concentrations declined in a mono- or bi-phasic manner and remained quantifiable up to between 12 to 24 h post-dose, giving rise to a geometric mean T1/2 of 3.26 h. Maximum and overall plasma exposure of SRP-3D (DA) based on geometric mean (geometric CV%) Cmax, AUC(0–24) and AUC(0-inf) were 2700 ng/mL (35.1%), 6870 ng.h/mL (39.0%) and 6970 ng.h/mL (39.5%), respectively. Increasing the dose from 600 to 900 mg also increased geometric mean Cmax and AUC(0–24) values by 1.6- and 1.2-fold.

Following a single administration of 900 mg SRP-001 oral suspension in the fed state, plasma concentrations of SRP-001 were observed from the first sampling time-point of 1 h post-dose for all subjects. Peak concentrations (Tmax) were reached between 1 and 8 h post-dose, after which concentrations declined in a mono- or bi-phasic manner and remained quantifiable up to between 12 and 24 h post-dose, giving rise to a geometric mean T1/2 of 3.79 h. Maximum and overall plasma exposure of SRP-001 based on geometric mean (geometric CV%) Cmax, AUC(0–24) and AUC(0-inf) were 1200 ng/mL (32.4%), 6250 ng.h/mL (22.3%) and 5910 ng.h/mL (17.1%), respectively. The relative bioavailabilities based on geometric mean Cmax, AUC(0–24), AUC(0-last) and AUC(0-inf) were 48.4% (21.9%), 99.2% (19.0%), 98.4% (16.3%) and 101% (20.3%), respectively. In a two-day oral dose toxicity and pharmacokinetic (PK) study of SRP-002 in Beagle dogs, peak plasma concentrations of SRP-001 were detected within a 30-min to 1-h window post-administration. Maximal plasma concentrations ranged from 3654 to 4879 ng/mL in the low-dose cohort and 9760–17,003 ng/mL in the high-dose cohort. Intriguingly, a nine-fold escalation in dose between the low and high-dose groups yielded only a 3–fourfold amplification in peak plasma concentration. This observation underscores a dose-dependent relationship in both AUC_0-inf_ and C_max_ values, in accordance with established PK principles. These results are congruent with the known PK behavior of compounds within this class.

Finally, PKs from the last SAD cohort (2000 mg fasted state) demonstrate that following SRP-001 oral suspension, plasma concentrations of SRP-001 are observed from the first sampling time-point of 1 h post-dose for all subjects. Peak concentrations (Tmax) were reached at 1 or 2 h post-dose, after which concentrations declined in a mono- or bi-phasic manner and remained quantifiable up to between 24 to 48 h post-dose, giving rise to a geometric mean T1/2 of 5.96 h. Maximum and overall plasma exposure of SRP-001 based on geometric mean (geometric CV%) Cmax, AUC(0–24) and AUC(0-inf) were 4590 ng/mL (34.0%), 18,900 ng.h/mL (25.7%) and 19,600 ng.h/mL (25.7%), respectively. Increasing the dose from 900 to 2000 mg resulted in a sub-proportional increase in geometric mean Cmax and AUC(0–24) values, with fold-increases of 1.7- and 2.8-fold, respectively, for the 2.2-fold increase in dose (Fig. 3h,i). The geometric mean Cmax and AUC(0–24) values represented 34% and 31% of the exposure limits defined in the protocol, respectively.

### Statistics

Changes in the withdrawal thresholds or latencies induced by a drug were first analyzed with a one-way ANOVA. Comparisons between the effects of different drugs were then subjected to t-test for unpaired means. A value of *p* < 0.05 was considered significant. All statistical analyses were performed using GraphPad Prism 9.5.1. Missing data points in the statistical analysis were addressed using Multiple Imputation, a robust technique that effectively mitigates missing values and minimizes bias. This methodology aligns with contemporary best practices and guidelines for clinical trials^82^. In contrast to the Last Observation Carried Forward (LOCF) method, which is prone to introducing bias, Multiple Imputation offers a more faithful representation of the data distribution.

#### Single-cell (scRNA) RNA sequencing

For cell nuclei isolation from the midbrain PAG, they were flash-frozen, dissected (by a cryotome), minced into small pieces, homogenized, filtered through 70 μ strainer and centrifuged. ReadiDrop 7-AAD cell viability dye (Bio-Rad) added, and flow sorted the live (7-AAD positive) nuclei in a BioRad Cell Sorter S3e using purity mode. Nuclei were counted using Nexcelom automated Cellometer with acridine orange/propidium iodide (AOPI) stain, and the Nuclei Stock Concentration used so that 10,000 nuclei could be targeted for each sample. Samples were bulk transposed and around 16,100 nuclei were loaded onto each channel of the microfluidic chip and GEMs generated using the 10 × Chromium controller, with the reagents from the Chromium Next GEM Single Cell ATAC Kit, 1,000,280 (10 × Genomics), following manufacturer instructions, followed by post-GEM cleanup with Dynabeads and SPRIselect (Beckman Coulter). Then after pre-amplification PCR, samples were divided for the ATAC and for the GEX library construction. Post library construction QC was performed using Agilent Bioanalyzer 2100 on High Sensitivity DNA chips, and after verification of sample traces, each library normalized based on the average fragment size, and the concentration determined using Qubit 4.0 fluorometer (Thermo Fisher Scientific). Pooled Libraries were then sequenced by using Illumina high output kits on a NovaSeq 6000 platform.

#### Data processing

Cell Ranger ARC (cellranger-arc) v.2.0.2 (10 × Genomics) was used to process Chromium Single Cell Multiome ATAC + Gene Expression (GEX) sequencing data. Briefly, raw BCL files from the Illumina NovaSeq were demultiplexed into paired-end, gzip-compressed FASTQ files that were generated using cellranger-arc *mkfastq* and default parameters. Read alignment, filtering, barcode counting, peak calling, and counting of both ATAC and GEX molecules were performed using cellranger-arc *count*. The rat genome mRatBN7 and its annotated transcriptome were used as the reference for alignment. Only confidently mapped reads with valid barcodes, unique molecular identifiers (UMIs), and non-PCR duplicated were retained. The overall sequencing quality was evaluated by looking at the summary metrics of the web_summary.html file generated for each sample. Processed data sets of multiple samples were then aggregated with cellranger-arc *aggr* to normalize input runs to the same median fragments per cell across samples. The linkage between chromatin accessibility and GEX was also established for each sample with this tool, as the ATAC and GEX measurements are on the very same cell.

### Seurat and single cell downstream analysis

Standard pre-processing through the Seurat workflow was performed for single-cell RNAseq data. Mitochondrial genes were identified, and cells expressing > 5 percent of mitochondrial features were removed. The dataset was log normalized with a size factor of 10,000 molecules for each cell using the NormalizeData function. Z-score transformation was performed across all cells to standardize expression values for genes using the ScaleData function^83^. Highly variable features for each group were identified using the FindVariableFeatures function. The top 10 variable features were plotted.

To visualize the data principal component analysis (PCA) was performed for dimensionality reduction, PCA dimensions were further reduced into two dimensions using UMAP. Weighted shared nearest neighbor and clustering of single cells was performed by Louvain algorithm. A table of logged twofold change and p values between each treatment group was calculated with Seurat. This calculation determines differential expression using non-parametric Wilcoxon rank sum test; these values were used in heatmaps and dot plots showing differential expression of top 50 DE and pain-related genes. Additionally, the top 50 differentially expressed genes were used in Gene Ontology enrichment analysis described below.

Cell type annotation was performed with the ScType package, which utilizes the largest database of human and mouse-specific cell markers to date^84^. The database was compiled by integrating CellMarker and PanglaoDB databases and manually curated 15 novel cell types by identifying marker genes from publications. The automatic cell annotations were based on clusters identified using Seurat PCA and UMAP, then integrated with the Seurat dataset for downstream analysis and visualization.

### Gene ontology enrichment analysis

Enrichment analysis was performed on the top 50 differentially expressed genes from comparisons between Vehicle vs CFA, CFA vs CFA_ApAP, and CFA vs CFA_SRP_001. The gene sets generated were used to determine the enrichment of GO categories, with R package clusterProfiler^85^. Visualization of the enrichment result object was achieved with R packages DOSE and enrichplot^86^. Visualization included: Barplots showing the enriched terms and corresponding enrichment score (p-values) and gene count, gene concept networks showing linkage of genes and GO terms, and enrichment maps showing the enriched terms organized into a network connecting overlapping gene sets.

### Qiagen ingenuity pathway analysis (IPA)

The genes with known gene IDs and their corresponding DESeq2 expression values were uploaded into the software using the batch upload feature. Each gene symbol was mapped to its corresponding gene object in the Ingenuity Pathways Knowledge Base. Unbiassed pathways and gene networks were algorithmically generated based on their connectivity and assigned a score. The score considers the number of focus genes in the network and the size of the network to approximate how relevant this network is to the original list of genes. The networks identified are then presented as a graph indicating the molecular relationships between genes/gene products. Upstream causal networks indicating potential/predicted upstream relationships based on the input gene list are also generated, as well as enrichment plots based on the most significant/highest-scoring pathways containing genes from the uploaded dataset. The “path designer” module was used to polish the network images. Data from IPA for canonical pathway enrichment was exported, and histograms were produced using RStudio 2021.09.0.

### Cell–cell communication analysis

To study intercellular communication, we applied the CellChat (version 1.5.0) R package^52^. First, the expression data, together with the identified cell clusters, were converted into a CellChat object using the “createCellChat” function. Known ligand-receptor pairs were obtained from the CellChatDB. Prior to running CellChat, the “subsetCellChat” function was used to filter out genes that were not included in the “CellChatDB”. Then, the “identifyOverExpressedGenes” and “identifyOverExpressedInteractions” functions were applied to identify cluster-specific ligand and receptor genes. The “computeCommunProb” function was utilized to calculate the communication probabilities among different cell clusters. We aggregated these probabilities using the “aggregateNet” function. To illustrate variations in the intensity of cell-to-cell communication, we utilized the “netVisual_diffInteraction” function. Following this, we employed non-negative matrix factorization (NMF) through the “identifyCommunicationPatterns” function to explore the underlying coordination among multiple cell clusters and signaling pathways. This helped us infer the number of distinct patterns governing intercellular communication. River plots were used to visualize the major signaling inputs and outputs among each of the cell types.

### Supplementary Information

Supplementary Information.

## Data Availability

All data supporting the experimental findings reported in this paper are available within the paper and its Supplementary Information. All the data are available from the corresponding authors upon reasonable request.

## Code availability

No custom codes were used for data analysis. All data analyses were performed using freely available open-source software for bioinformatics from Bioconductor and GitHub.
