# Supplementary material for: Transcriptomic signature, bioactivity and safety of a non-hepatotoxic analgesic generating AM404 in the midbrain PAG region
Source: Sci Rep. 2024 May 15;14:11103. doi: 10.1038/s41598-024-61791-z (PMC11096368; doi:10.1038/s41598-024-61791-z)
Supplement: Supplementary file 1 — Supplementary Information. [file 41598_2024_61791_MOESM1_ESM.docx]

**Supplementary Information**

**Transcriptomic signature, bioactivity and safety of a non-hepatotoxic analgesic generating AM404 in the mid-brain PAG region**

Hernan A. Bazan^1^*^†^, Surjyadipta Bhattacharjee^2†^, Madigan M. Reid^2^, Bokkyoo Jun^2^,  Connor Polk^2^, Madeleine Strain^2^, Linsey A. St Pierre^2^, Neehar Desai^2^, Patrick W. Daly^2^, Jessica A. Cucinello-Ragland^3^, Scott Edwards^2,3^, Javier Recio^4^, Julio Alvarez-Builla^4^, James J. Cai^5,6^, and Nicolas G. Bazan^2^*

^1^Section of Vascular/Endovascular Surgery, Department of Surgery, Ochsner Clinic, New Orleans, LA 70118, USA

^2^Neuroscience Center of Excellence, School of Medicine, Louisiana State University Health New Orleans, New Orleans, LA 70112, USA

^3^Department of Physiology, School of Medicine, Louisiana State University Health New Orleans, New Orleans, LA 70112, USA

^4^Department of Organic Chemistry and IQAR, University of Alcala, Alcala de Henares, Madrid 28805 Spain

^5^Department of Electrical and Computer Engineering, Texas A&M University, College Station, TX 77843, USA

^6^Department of Veterinary Integrative Biosciences, Texas A&M University, College Station, TX 77843, USA

^†^Equal first authors

*Corresponding authors: hbazan@ochsner.org and nbazan@lsuhsc.edu

Contents:

Supplementary Figs. 1-20

Supplementary Tables 1-2

**Supplementary Figures**

**Supplementary Fig. 1.** ApAP metabolic pathways, there is pronounced hepatotoxicity when NAPQI accumulation overwhelms glutathione conjugation*.*


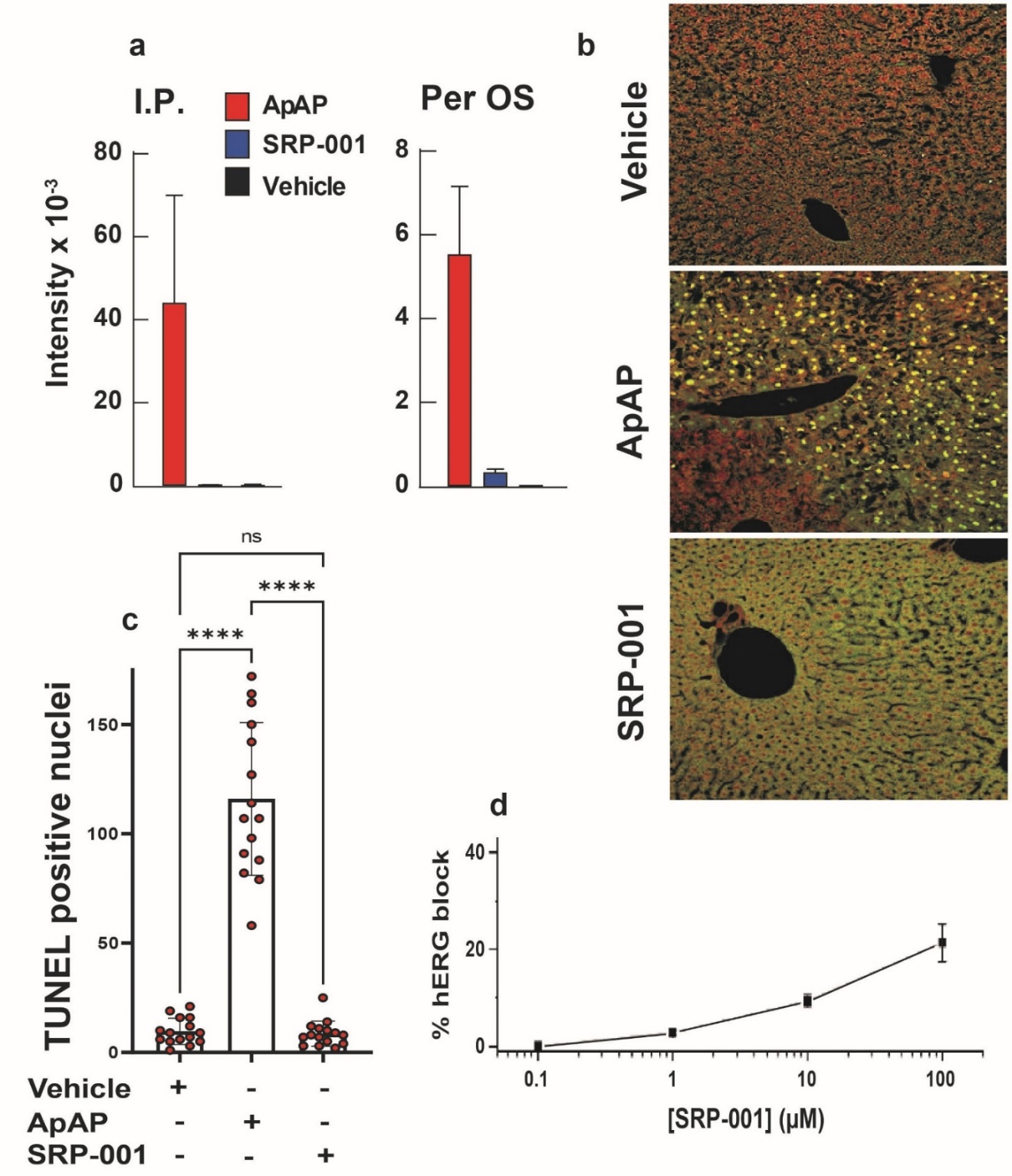


**Supplementary Fig. 2. SRP-001 is non-hepatotoxic and non-cardiotoxic.**

**a**, Histograms demonstrate that the serum NAPQI levels are significantly enhanced only for the ApAP-injected animals and not for vehicle- or SRP-001-injected animals. The significant rise in NAPQI levels for APAP-injected animals is regardless of the administration route (for both *IP* and *per os*). Hence, SRP-001 is considered non-hepatotoxic with the lack of production of NAPQI as compared to ApAP. **b**, TUNEL staining shows prominent TUNEL positive nuclei, indicating more apoptotic cells in ApAP dosed animals (600mg/kg), while vehicle and SRP-001 dosed animals (600 mg/kg) have almost no TUNEL signal. **c**, Quantification of TUNEL staining from liver sections from mice dosed with either vehicle or ApAP or SRP-001 (n=15) (p<0.0001). **d,** SRP-001 is considered non-cardiotoxic as evidenced by an absent relevant signal in an *in vitro* hERG assay in human embryonic kidney cells.


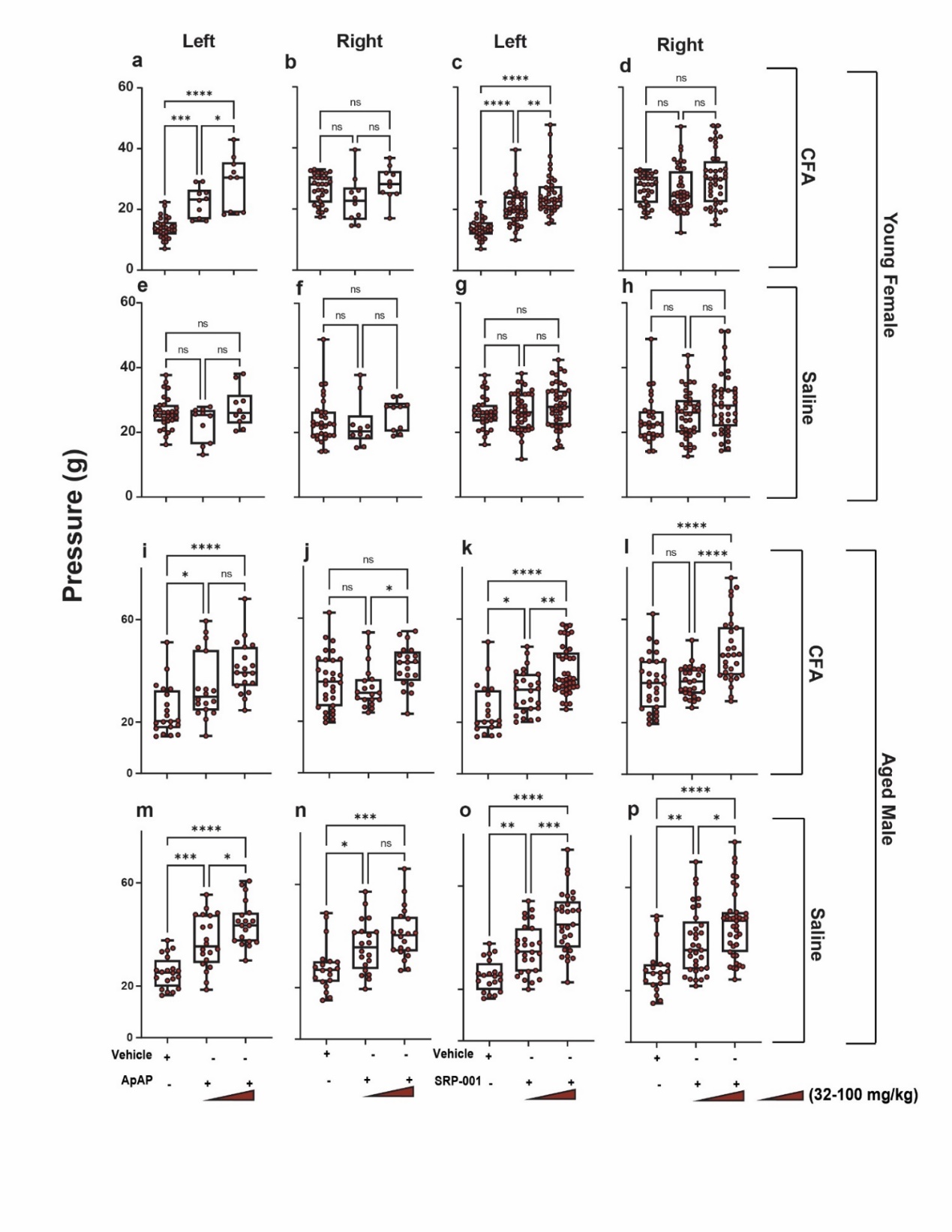


**Supplementary Fig. 3. Von Frey for young female and aged male rats.**

**a-h**, Von-Frey data of young female rats. **i-p**, Von Frey data of aged male rats. For the cohort of young female and aged male rats, two separate doses of ApAP and SRP-001—32 and 100 mg/kg body weight —were tested, respectively. SRP-001 oral nanosuspension and ApAP showed comparable analgesic activity for both animal cohorts (**a**, **i**) For SRP-001-treated animals, in the CFA-injected left hind paw, the threshold for paw withdrawal increased from 25 g to 32 g and subsequently to 40 g for (32 mg/kg and 100 mg/kg) dose, respectively. (*n=20*) rats for each treatment group for both cohorts.


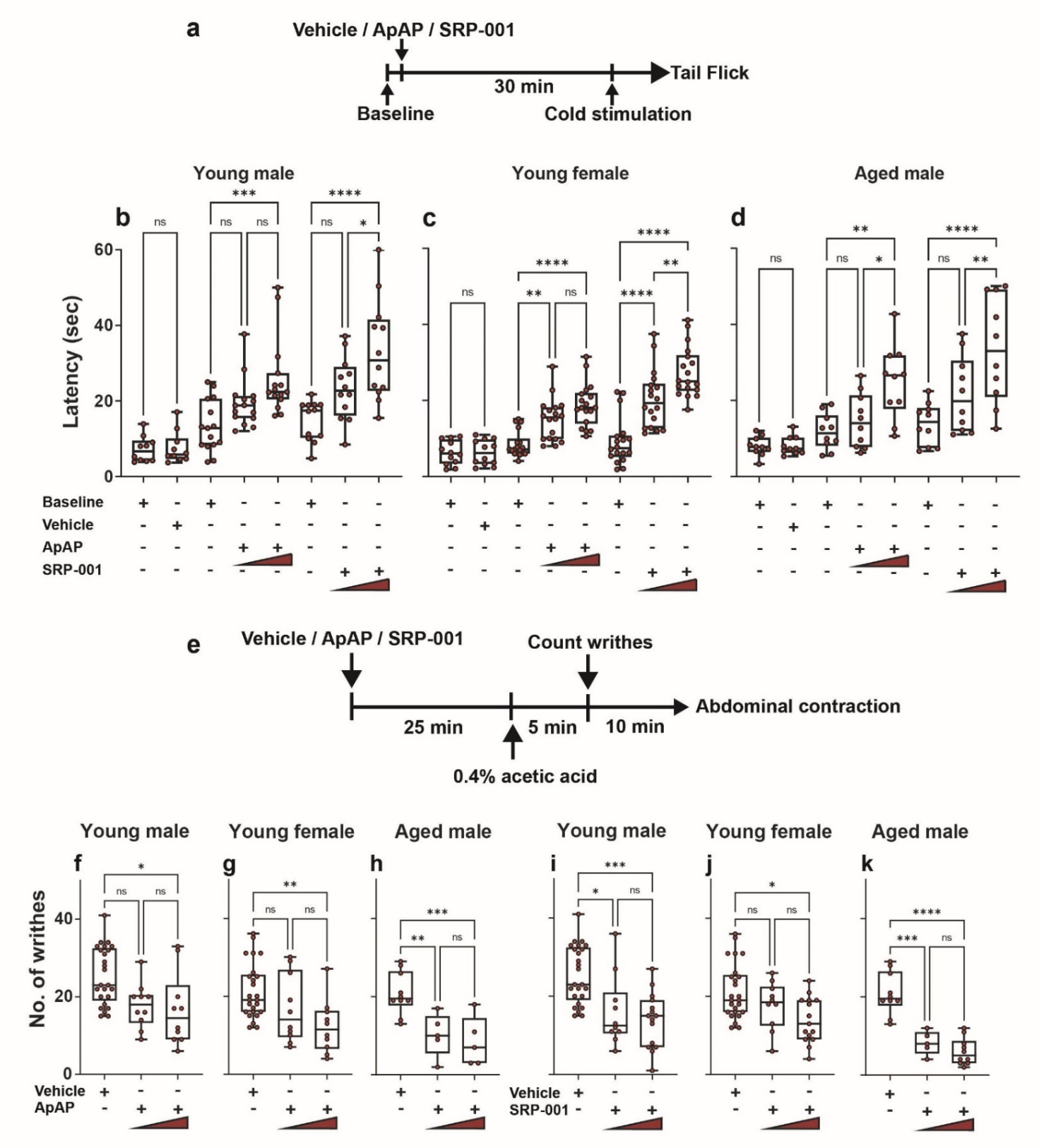


**Supplementary Fig. 4. Tail Flick (somatic pain) and Acetic acid writhing (visceral pain) assays.**

**a**, Timeline showing the experimental design of the cold tail-flick assay. **b**,**c**, Cold tail-flick assay of young (male and female). **d**, aged (male) mice. At the high dose (100 mg/kg) of ApAP or **SRP-001**, there is significant analgesia in all experimental cohorts. (*n=10*) mice for young male and aged male experimental groups and (*n=20*) or young female mice. **e**, Timeline showing the experimental design of the acetic acid-induced writhing assay of aged (male) and young (male and female) mice. Experimental groups: young female (**f**, **i**), young male (**g**, **j**), and aged male (**h** ,**k**) for ApAP and SRP-001 respectively. At the high dose (100 mg/kg) of ApAP or SRP-001, there is significant analgesia in all experimental cohorts. (*n=10*) mice per treatment group for aged male mice, (*n=20*) mice for vehicle, and (*n=10*) per treatment group for young male and young female.


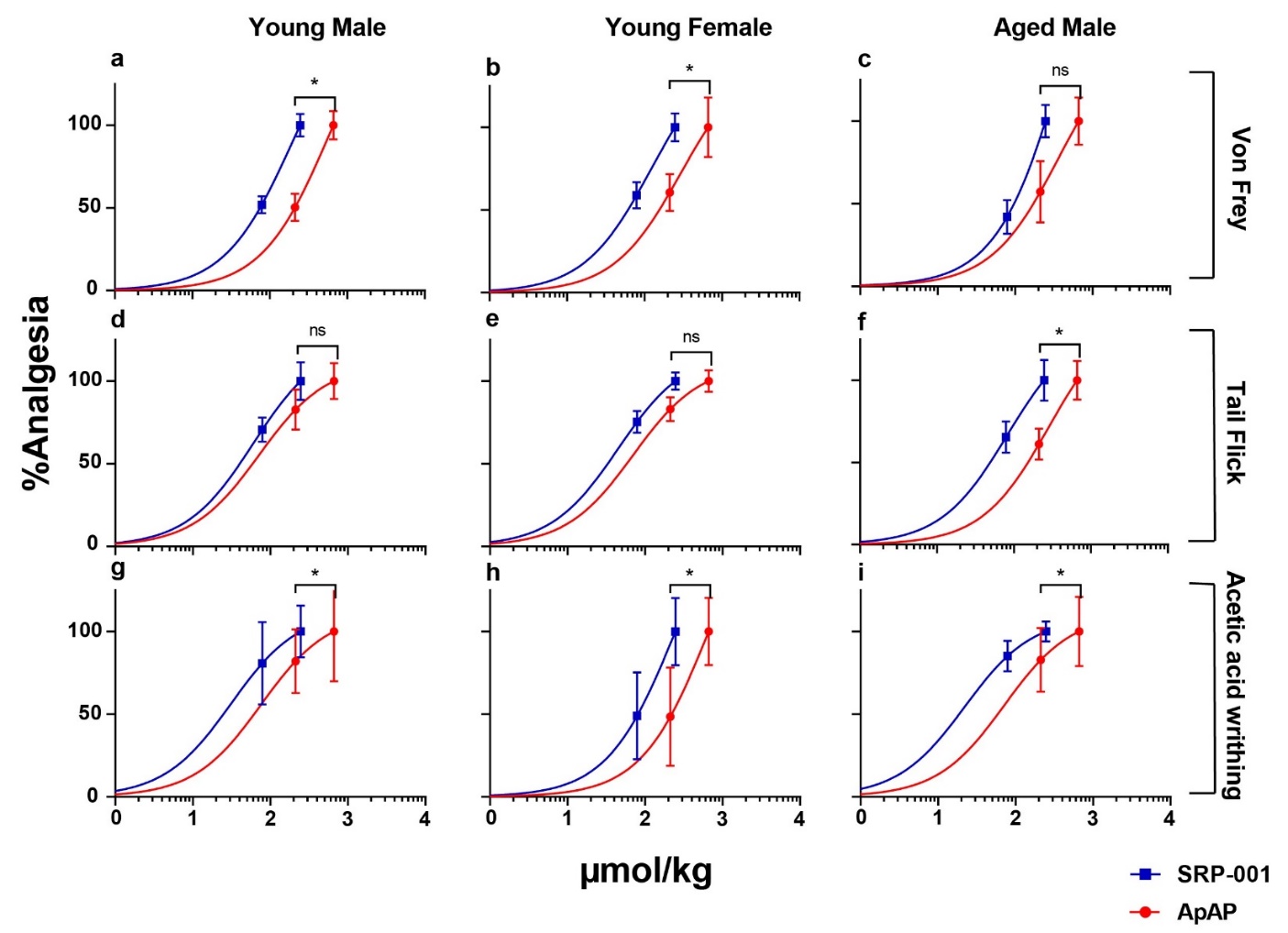


**Supplementary Fig. 5. Equimolar dose-response curves (µmol/kg) for Von Frey, tail flick, and acetic acid writhing assays.** Dose-response curves of von Frey (**a-c**, hyperalgesia), tail flick (**d-f**, somatic pain), and acetic acid writhing/abdominal contractions (**g-i**, visceral pain) assays for young male and female animals and aged male animals. All the different analgesic assays and resulting dose-response curves show a clear shift to the left of the SRP-001 curve compared to the dose-response curve of ApAP with similar equimolar doses of both drugs.


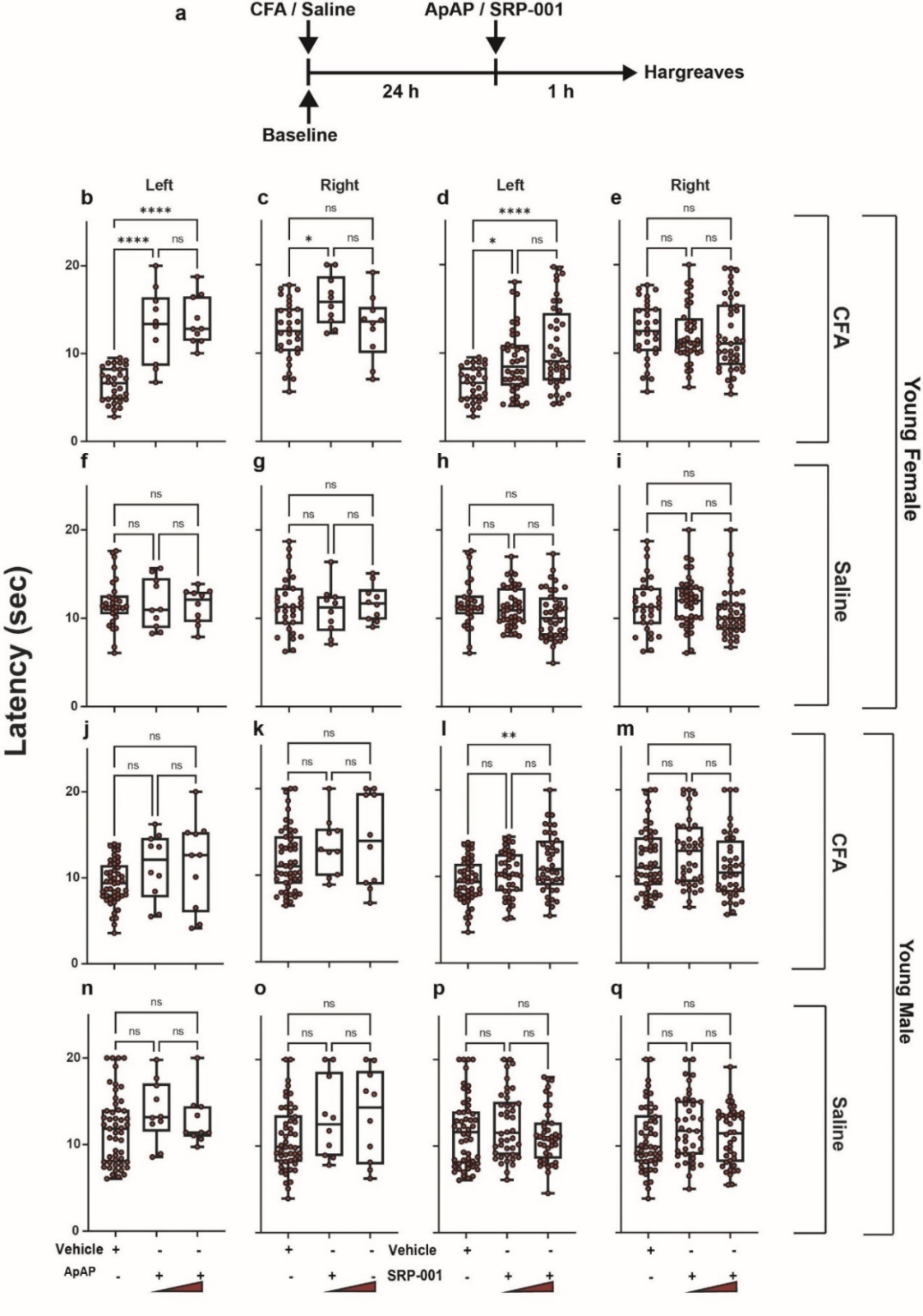


**Supplementary Fig. 6. Hargreaves assay for young male and young female rats.**

**a**, Timeline showing the experimental design of the Hargreaves assay for young female, young male, and for aged male rats (data showed in **Supplementary Fig. 7**). **b-i**, Paw withdrawal latencies for young male rats and **j-q**, for young female rats. At the high dose (100 mg/kg) of ApAP or SRP-001, there is significant analgesia in all experimental cohorts. (*n=20*) rats for each treatment group.


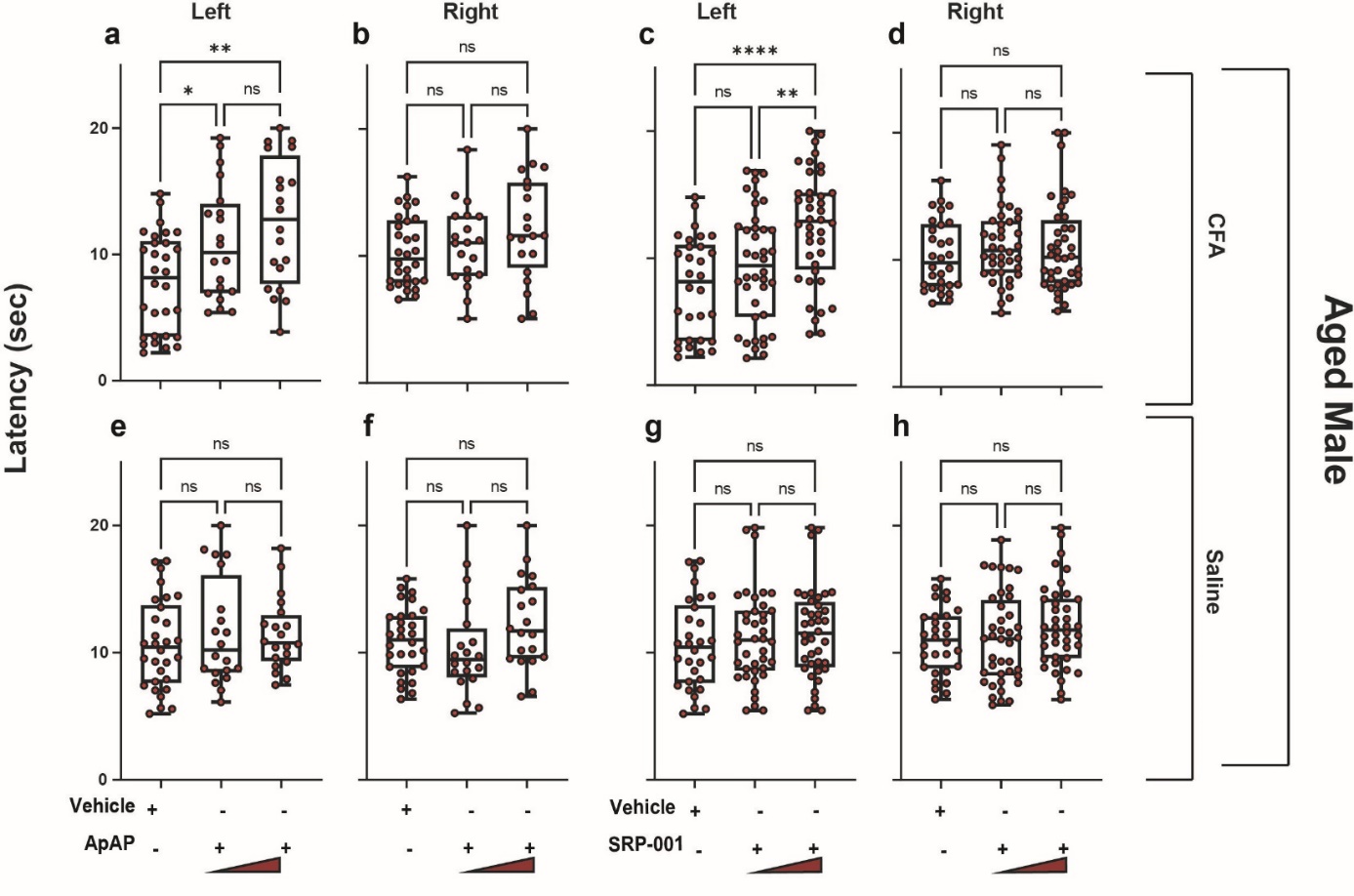


**Supplementary Fig. 7. Hargreaves assay for aged male rats.**

**a-h**, Paw withdrawal latencies for aged male rats. At the high dose (100 mg/kg) of ApAP or SRP-001, there is significant analgesia in all experimental cohorts. (*n=20*) rats for each treatment group.


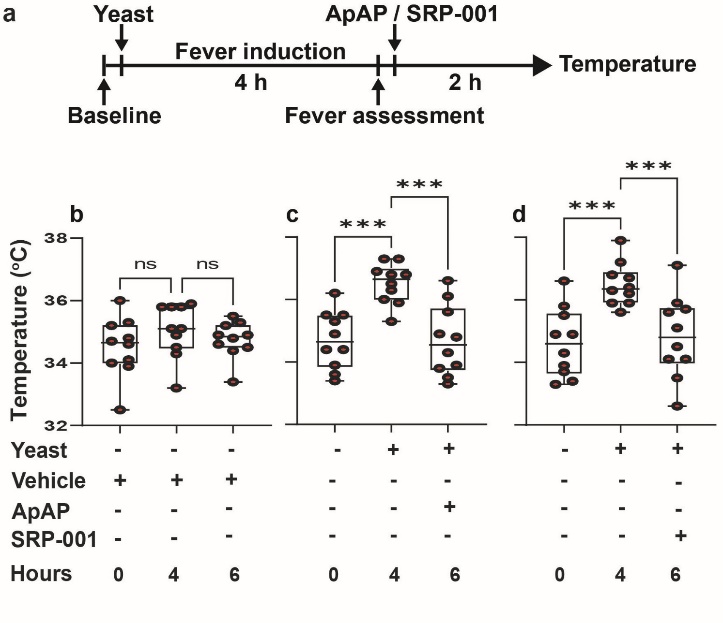


**Supplementary Fig. 8. Antipyresis-yeast.**

**a**, Timeline for experimental design of fever induction by yeast derived from *Saccharomyces cerevisiae* Type II, and subsequent administration of either ApAP or SRP-001 at 75 mg/kg body weight. **b**, No significant changes in body temperature of mice injected with 0.9% saline (vehicle) (Sham) throughout the course of the experiment. **c**, **d**, after yeast injection, there is significant fever induction, followed by a subsequent reduction in core body temperature with the *per os* administration of either ApAP or SRP-001. (*n=10*) mice per treatment group.


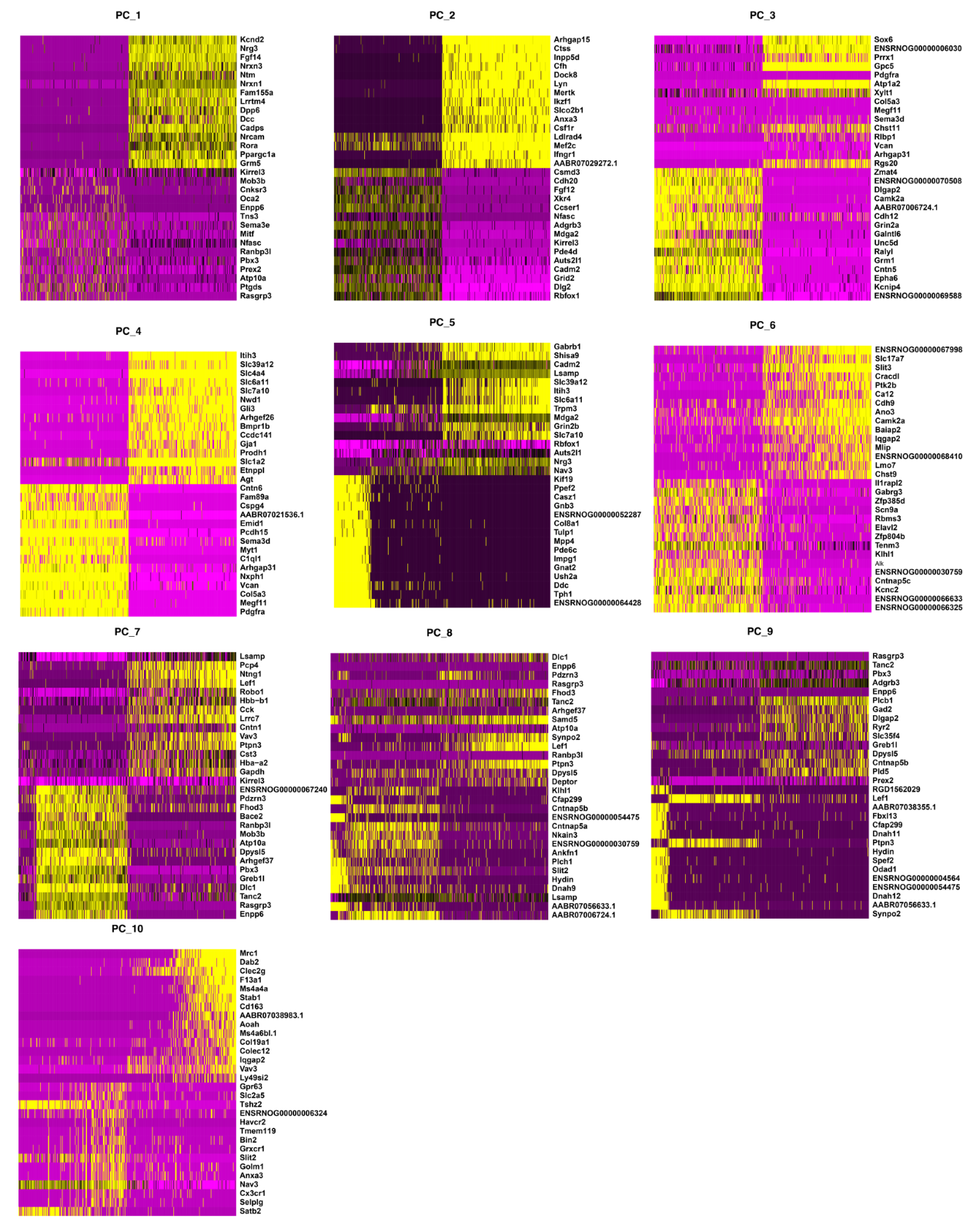

**Supplementary Fig. 9.** **PCA Dimension Plots –** Single cell heatmaps showing the top 30 differentially expressed genes in each cluster for the first 10 principal clusters (PCs) selected based on the elbow plot.


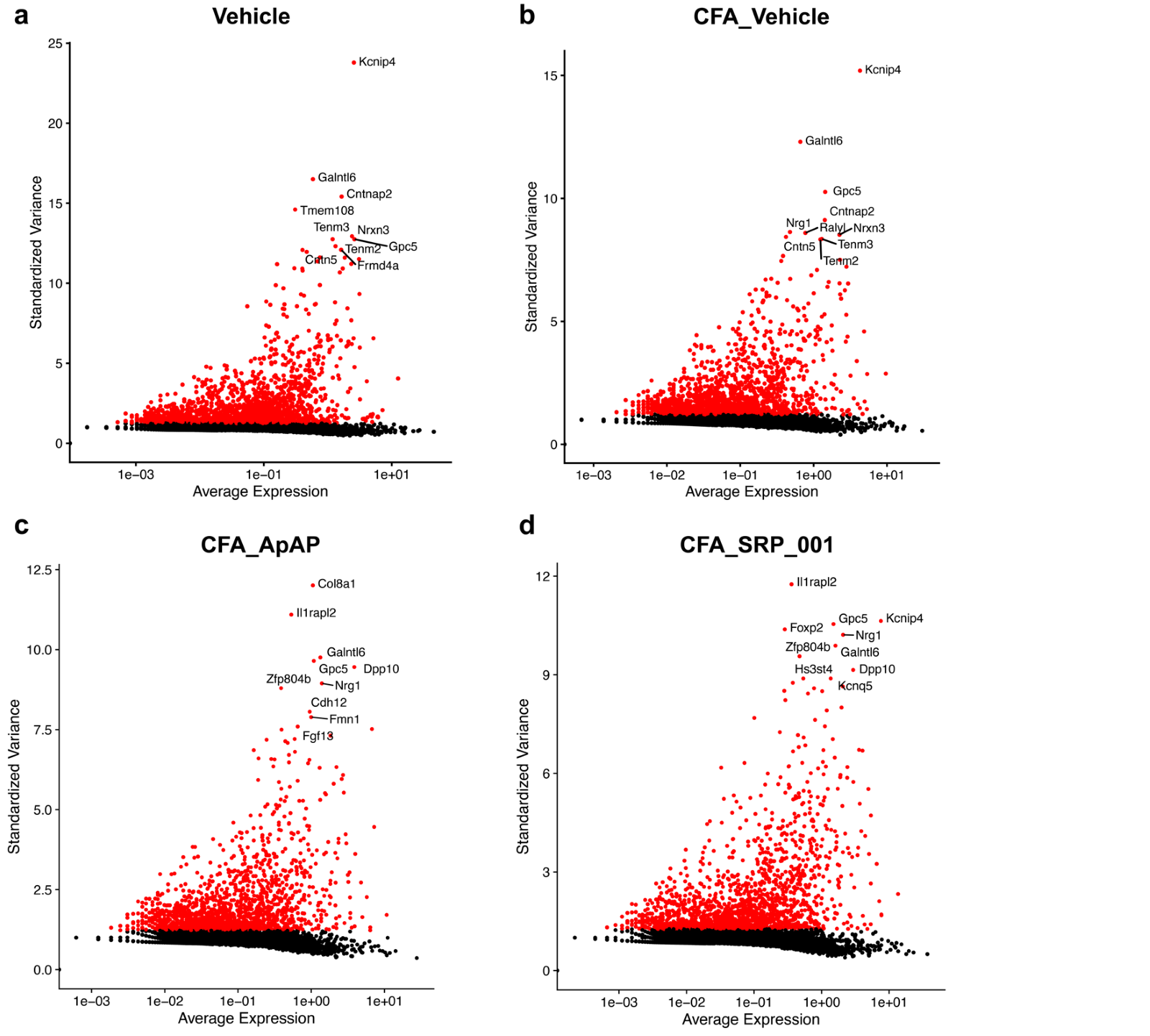


**Supplementary Fig. 10.** **Highly** **Variable Gene** **(HVG)** plots for each sample show top 10 HVGs across all cell clusters for each sample – **Vehicle**, **CFA_Vehicle**, **CFA_ApAP**, and **CFA_SRP-001**.


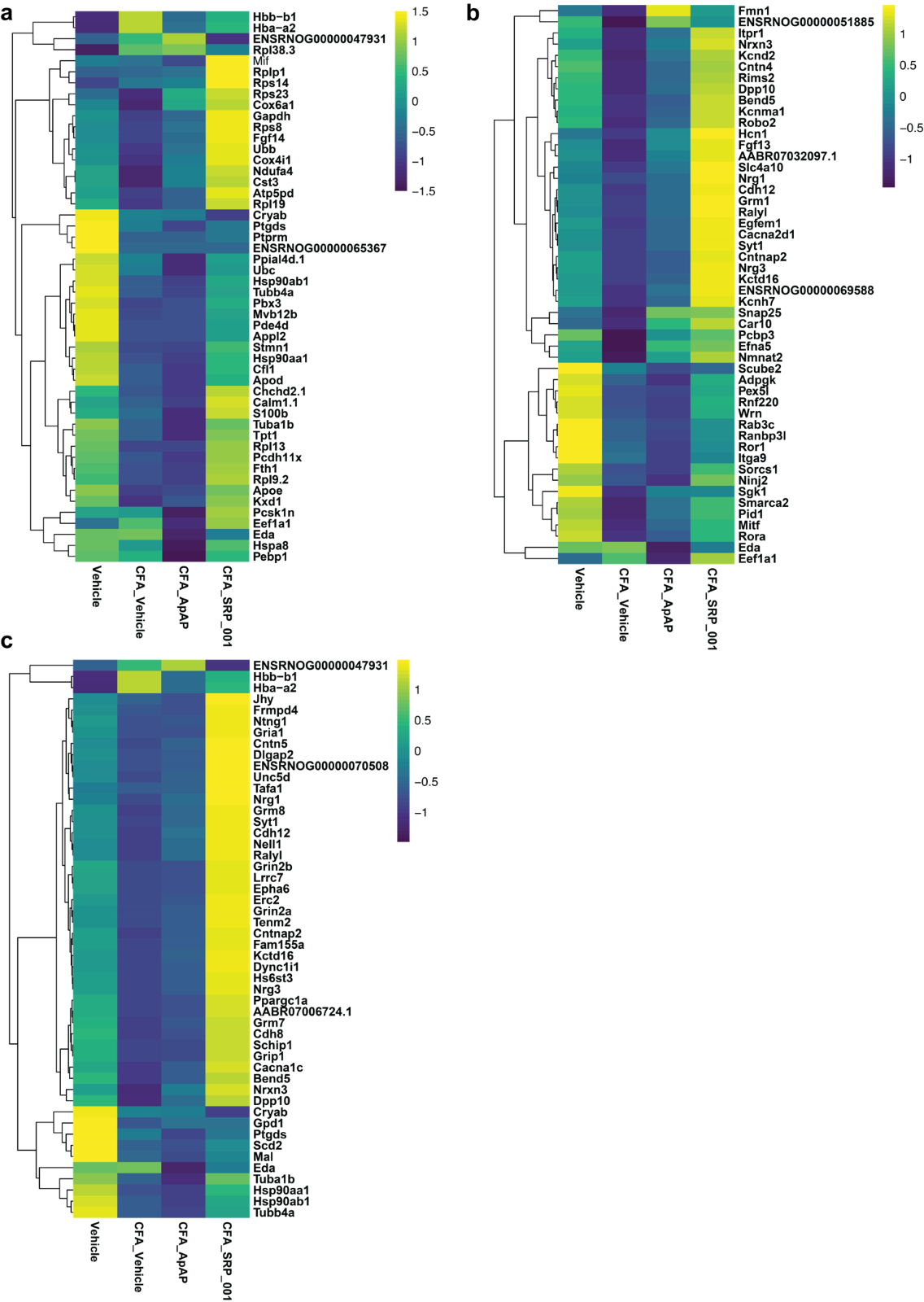


**Supplementary Fig. 11.** Heatmap of top 50 differentially expressed genes from DE analysis using Seurat for all samples showing comparison between Vehicle and CFA_Vehicle, and CFA_Vehicle vs CFA_ApAP and CFA_Vehicle vs CFA_SRP-001.


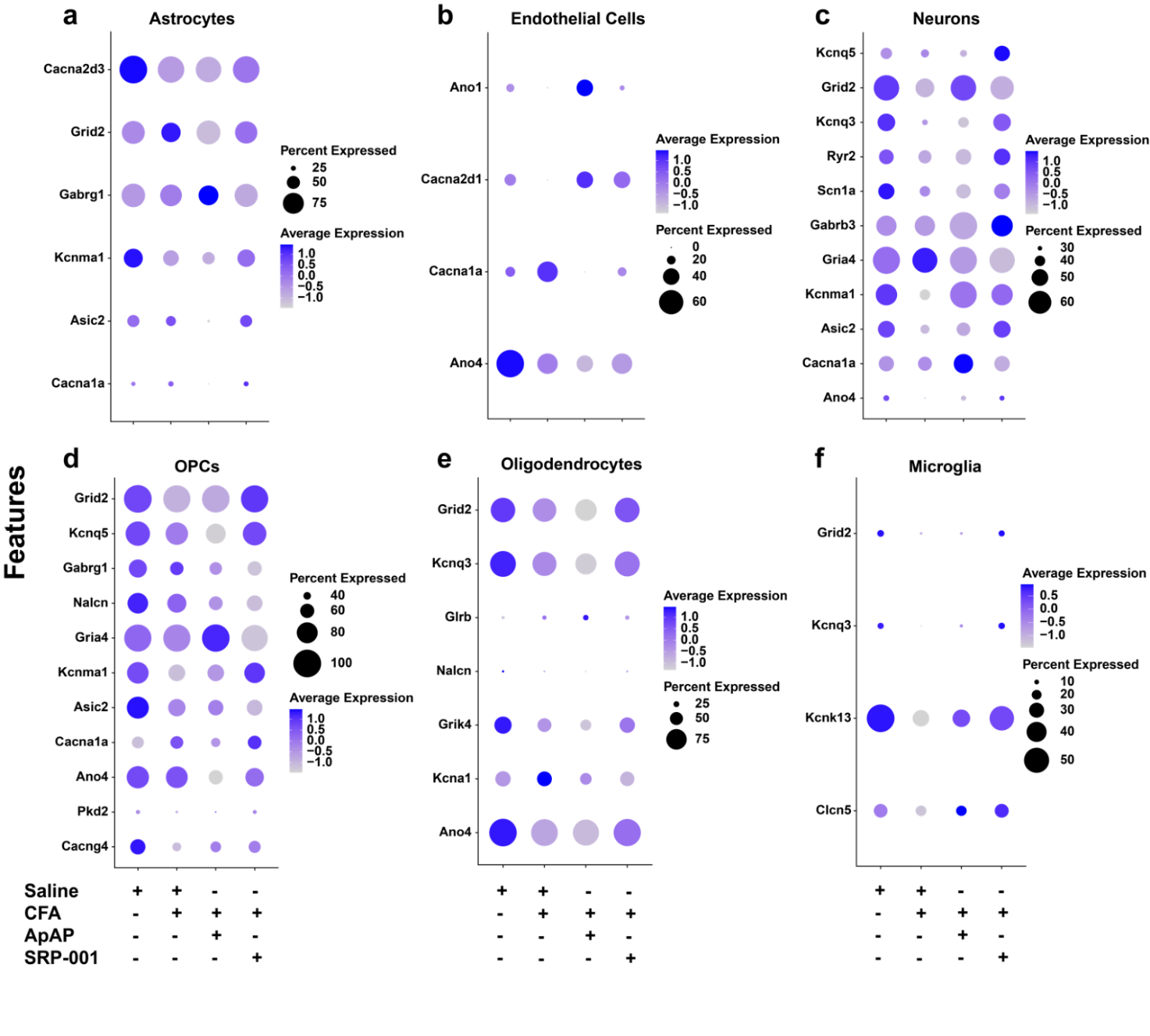


**Supplementary Fig. 12.** **Dot plots showing differential expression of pain-related genes – Ion Channels across treatment groups –** **Saline (Vehicle), CFA_Vehicle, CFA_ApAP, and CFA_SRP-001, in different cell clusters – a,** Astrocytes, **b,** Endothelial cells, **c,** Neurons, **d,** OPCs, **e,** Oligodendrocytes, and **f,** Microglia. The size of the dots represents the percentage of barcodes within a cluster and the color corresponds to the average expression (scaled data) within a cluster for each gene shown.


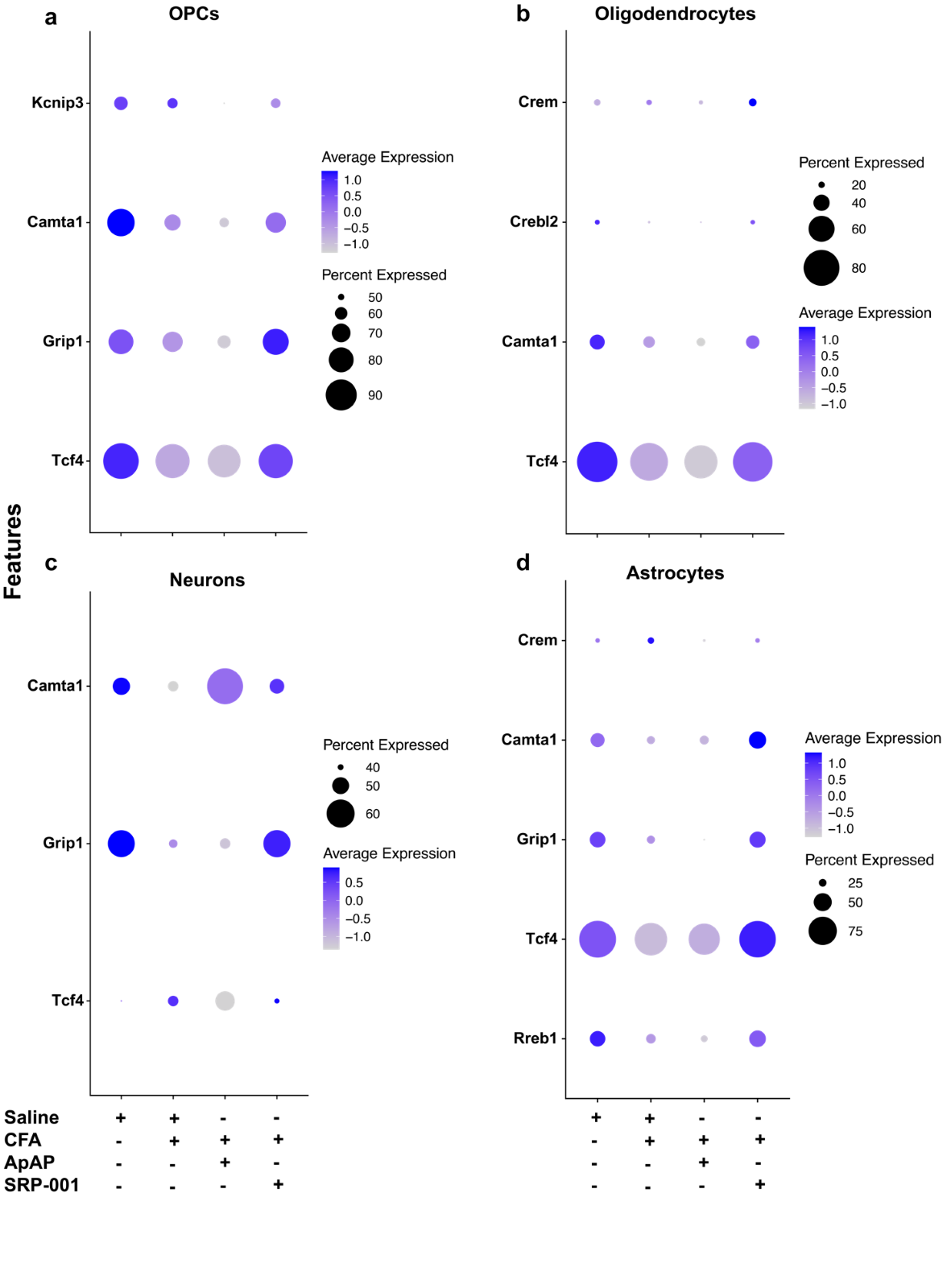


**Supplementary Fig. 13.** **Dot plots showing differential expression of pain-related genes – Transcription factors across treatment groups –** **Saline (Vehicle), CFA_Vehicle, CFA_ApAP, and CFA_SRP-001, in different cell clusters – a,** OPCs, **b,** Oligodendrocytes, **c,** Neurons, and **d,** Astrocytes.


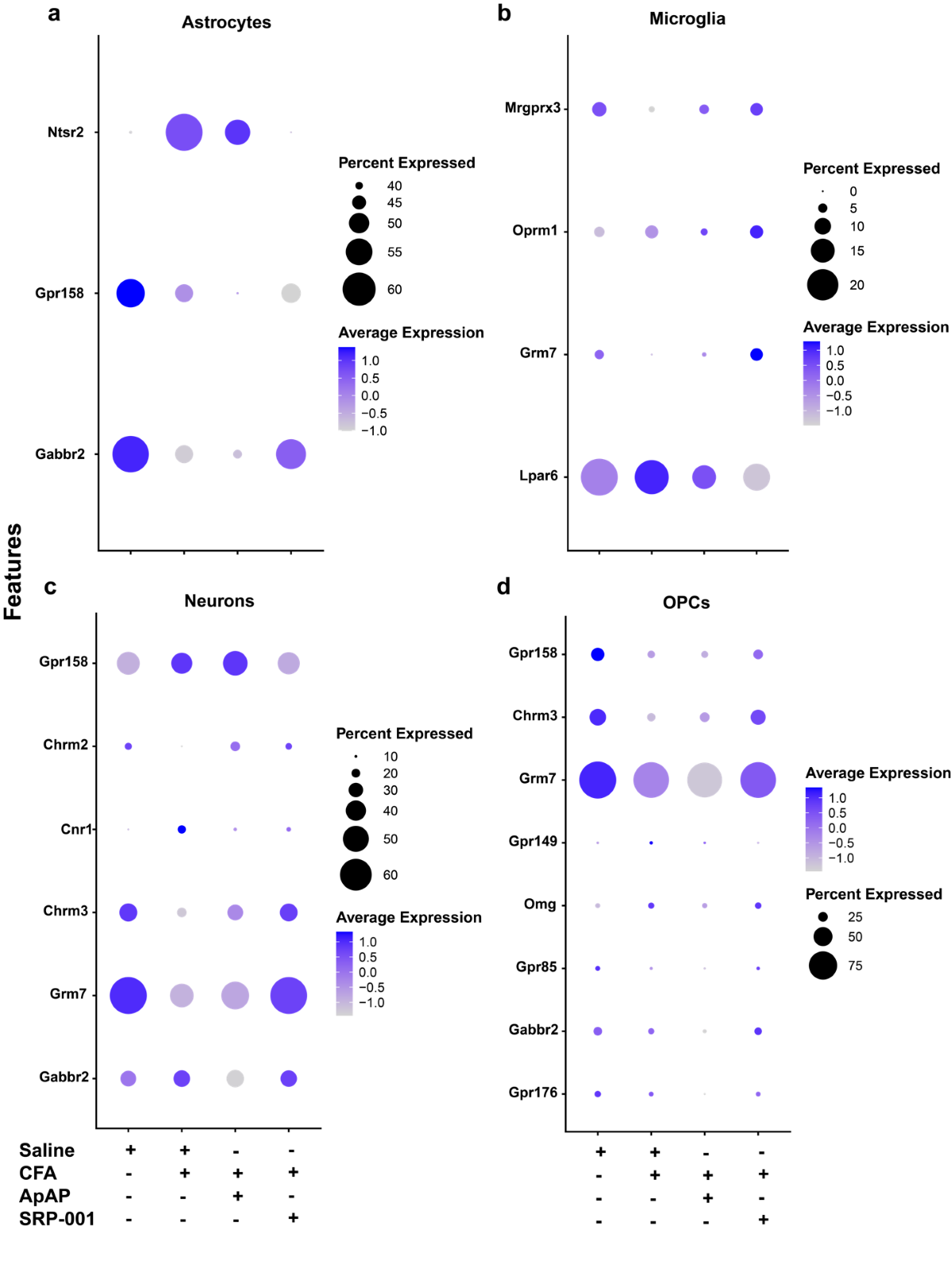

**Supplementary Fig. 14.** **Dot plots showing differential expression of pain-related genes – GPCRs across treatment groups –** **Saline (Vehicle), CFA_Vehicle, CFA_ApAP, and CFA_SRP-001, in different cell clusters – a,** Astrocytes, **b,** Microglia, **c,** Neurons, and **d,** OPCs.

***
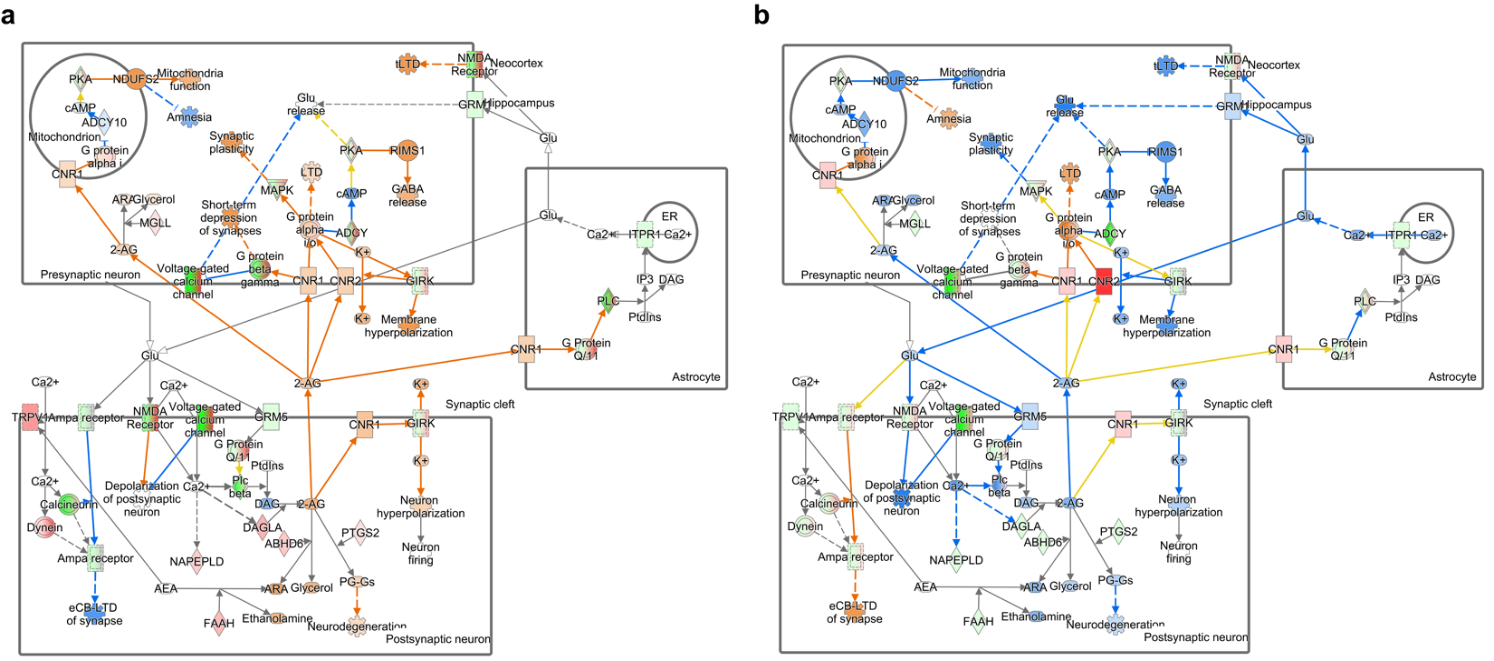
***

**Supplementary Fig. 15. Endocannabinoid signaling pathway associated gene regulation in Vehicle vs CFA_Vehicle and CFA_Vehicle** vs **CFA_ApAP** or **CFA_Vehicle** vs **CFA_SRP-001 comparisons.** Pathway overlay red and green color indicates decreased or increased expression measured experimentally respectively. Orange and blue overlay indicated predicted molecular relationships based on dataset measurements of genes know to interact/effect the predicted molecule/function. **a,** Expression and prediction overlay from DSeq2 comparison values between **CFA_Vehicle vs CFA_ApAP**. **b,** Expression and prediction overlay from DSeq2 comparison values **between CFA_Vehicle vs CFA_SRP-001.**

**
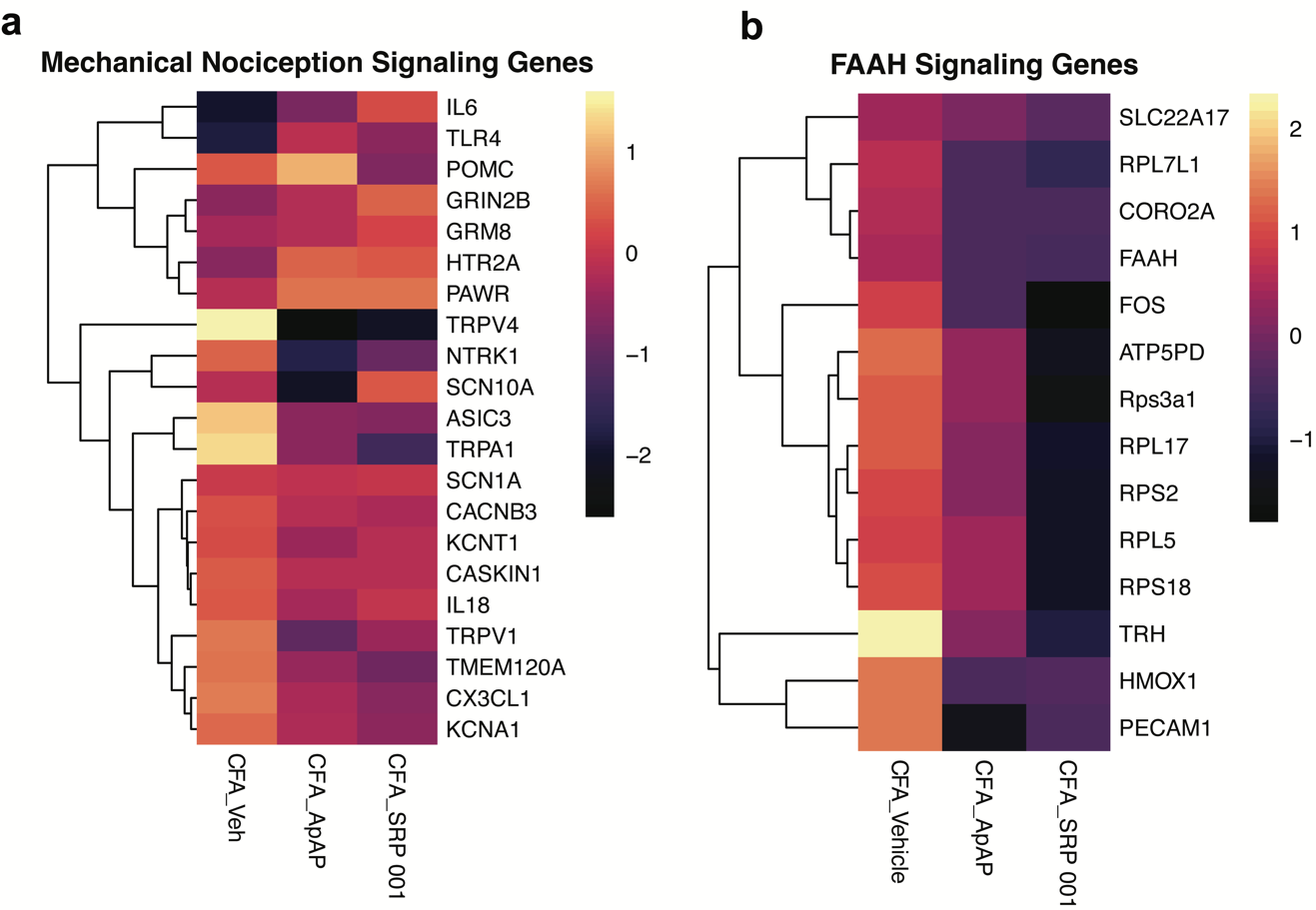
**

**Supplementary Fig. 16. Gene Network Expression for mechanical nociception and FAAH pathways generated by Ingenuity Pathway Analysis (IPA)**.

**a,** Heatmap of DSeq2 computed expression values from scRNAseq data involved in **genes pertaining to** **mechanical nociception**. Similarities in the **gene modulatory mechanism of ApAP and SRP-001** are highlighted by the shared trend in expression values of the genes shown. **b,** Heatmap of DSeq2 computed expression values for **FAAH related genes**. Similarities in the gene modulatory mechanism of ApAP and SRP-001 are highlighted by the shared trend in expression values of the genes shown.


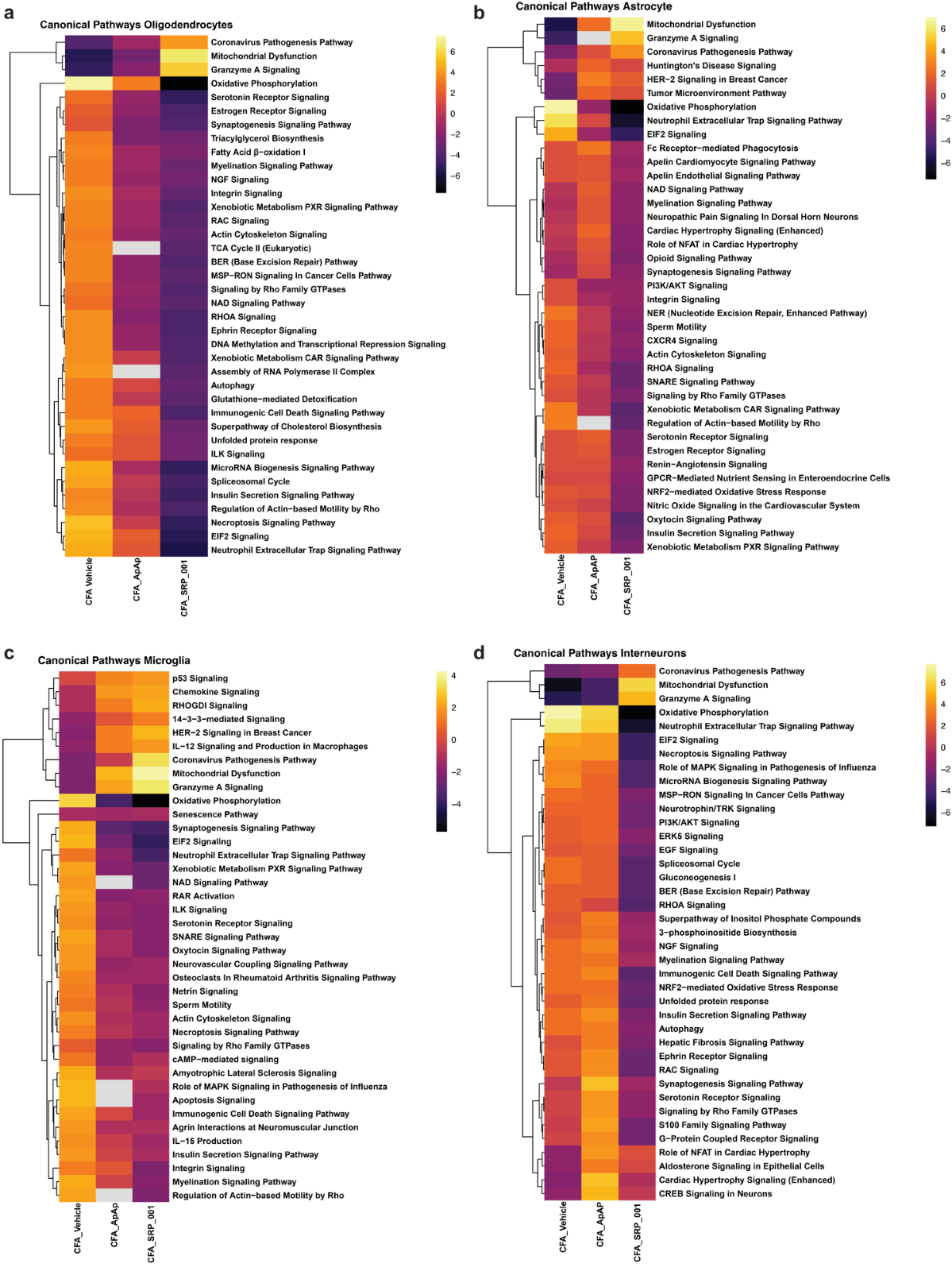


**Supplementary Fig. 17. Heat Maps of Canonical Pathways for different cell clusters.** a, Oligodendrocytes, b, Interneurons, c, Microglia, d, Astrocytes across the 4 sample groups – **Saline (Vehicle)**, **CFA_Vehicle**, **CFA_ApAP** and **CFA_SRP-001** with the comparisons **– CFA_Vehicle vs Vehicle, CFA_Vehicle vs CFA_ApAP and CFA_Vehicle vs CFA_SRP-001** treatment groups.


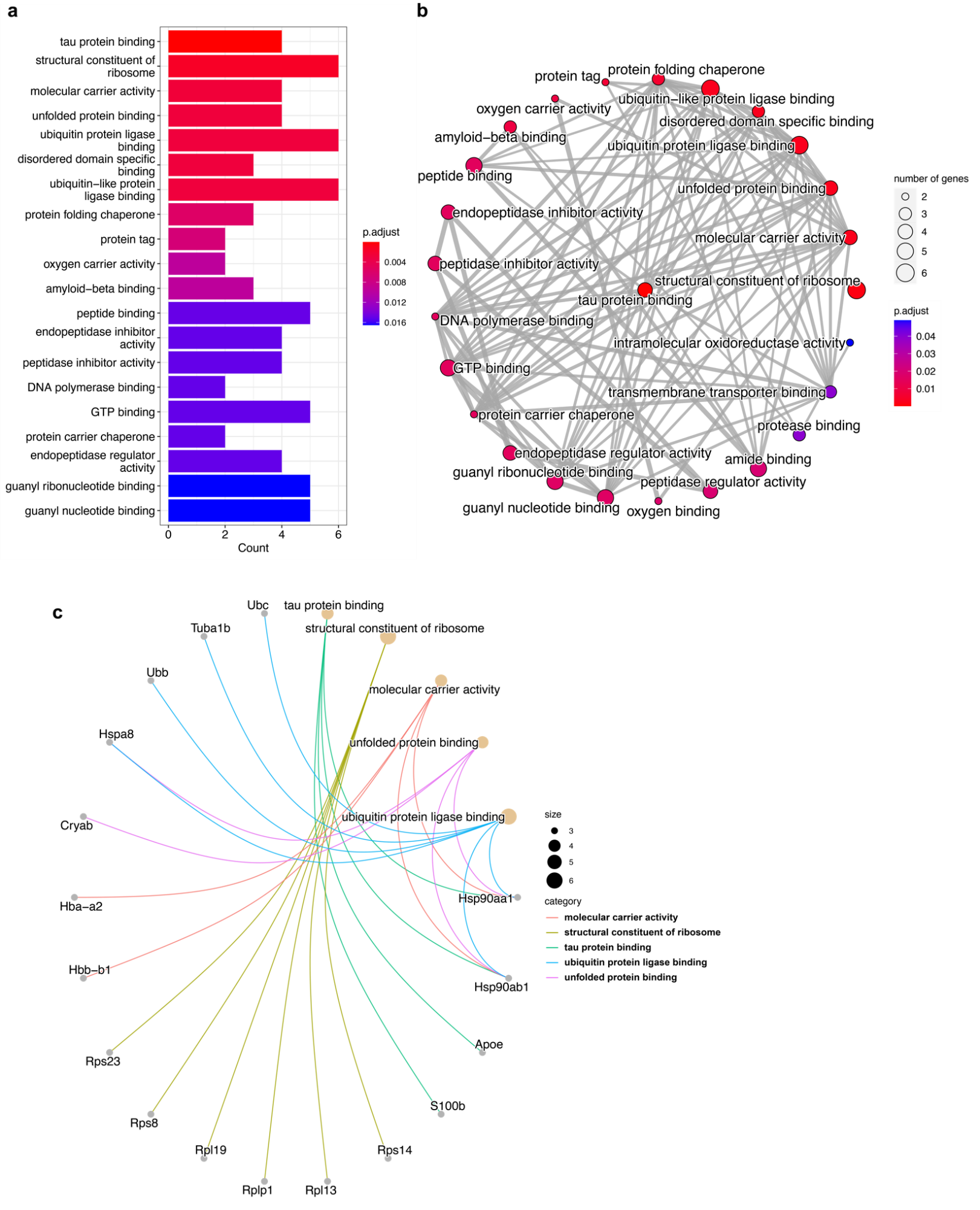


**Supplementary Fig. 18. GO enrichment analysis results for top 50 differentially expressed genes between Vehicle and CFA_Vehicle a,** Barplot of the enriched GO terms from the selected genes **b**, enrichment map of the GO terms showing linkage between the terms based on overlapping gene sets **c,** gene concept network showing the linkage between the DE genes and GO terms. ­­­


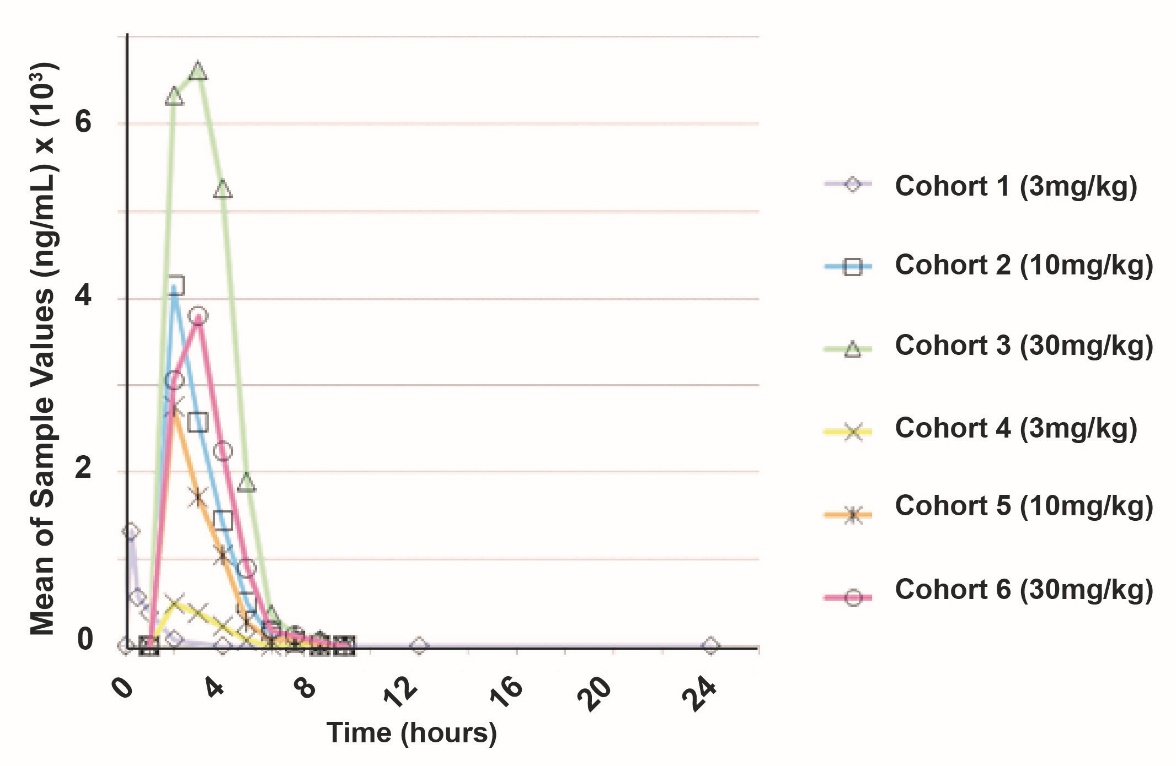


**Supplementary Fig. 19. Toxicokinetic (TK) beagle dog data.** Following IV & PO Dosing in beagle dogs, SRP-001’s uptake is rapid with Cmax in 15-30 min, and there is a dose-dependent response. Furthermore, SRP-001 is considered non-cardiotoxic, as evidenced by 1) a lack of findings in 28-day studies in beagle dogs or rats and 2) a normal telemetered safety pharmacology study in dogs (no prolonged QT_c_; data not shown for brevity).


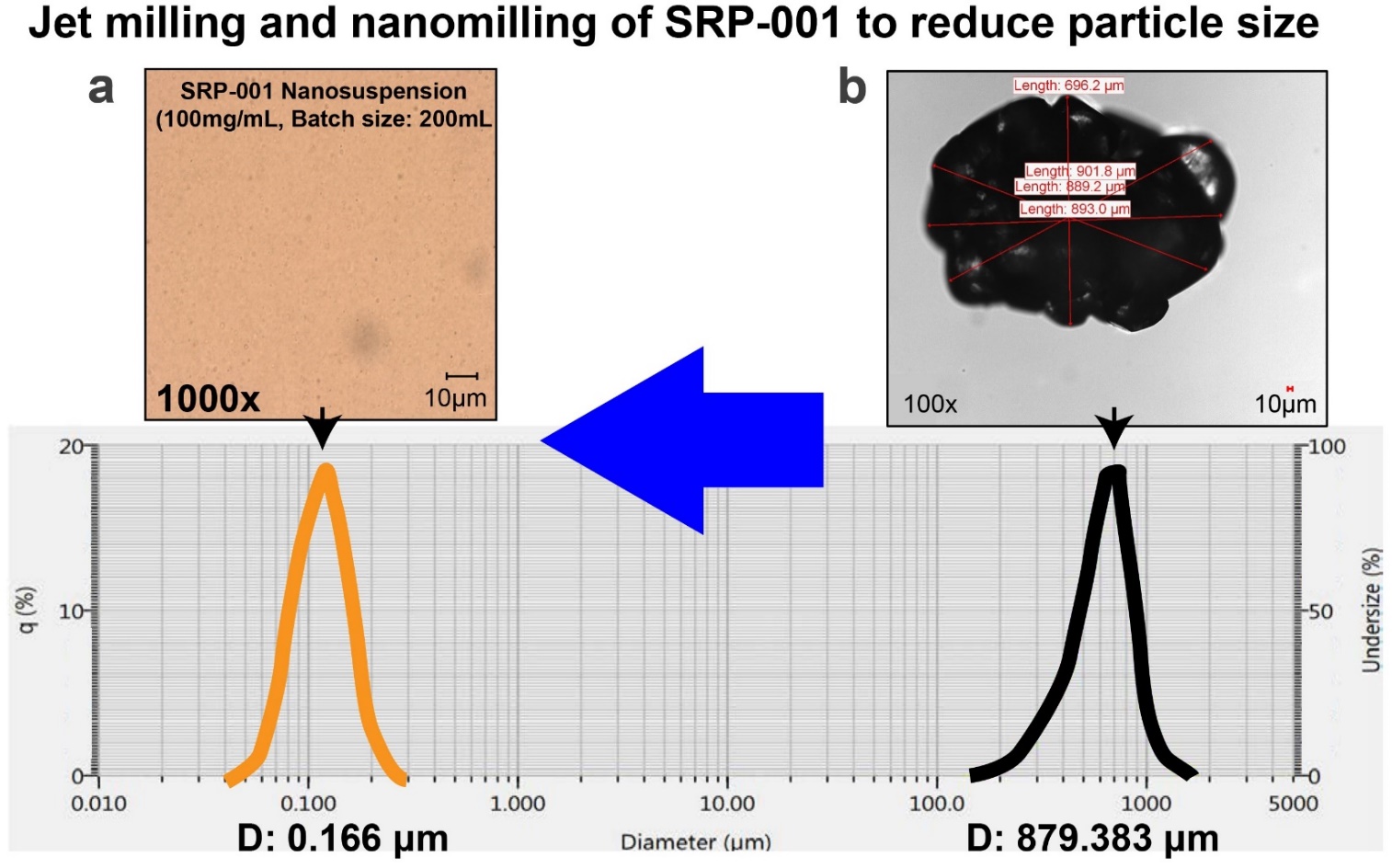


**Supplementary Fig. 20. Oral formulation: Jet milling nano-milling.** Jet milling and nano-milling of SRP-001 for 17 h reduce particle size from 879.33 µm to 0.1666 µm and increase the solubility of SRP-001 to optimize for both oral (chronic pain) and IV (acute pain) formulations.

**Supplementary Tables**

**Supplementary Table 1: Summary of ED_50_ values calculated from dose response curves generated by equimolar dosing (µmol/kg) of ApAP and SRP-001 in the different antinociceptive/analgesia assays.**

| **Analgesia Assay** | **ED_50_** | **Aged (Male) (μmol/Kg)** | **P value** | **Young (Male) (μmol/Kg)** | **P value** | **Young (Female) (μmol/Kg)** | **P value** |
| --- | --- | --- | --- | --- | --- | --- | --- |
| Tail Flick Assay (Mice) | ApAP | 281.154 | P <0.05 | 71.848 | P >0.05 (ns) | 70.258 | P >0.05 (ns) |
|  | SRP-001 | 81.64 |  | 59.98 |  | 44.924 |  |
| von-Frey Assay (Rats) | ApAP | 358.489 | P >0.05 (ns) | 573.582 | P <0.05 | 291.645 | P <0.05 |
|  | SRP-001 | 454.327 |  | 189.811 |  | 120.911 |  |
| Acetic acid writhing Assay  (Mice) | ApAP | 71.607 | P <0.05 | 75.822 | P <0.05 | 660.862 | P <0.05 |
|  | SRP-001 | 22.079 |  | 31.068 |  | 237.039 |  |

**Supplementary Table 2:**


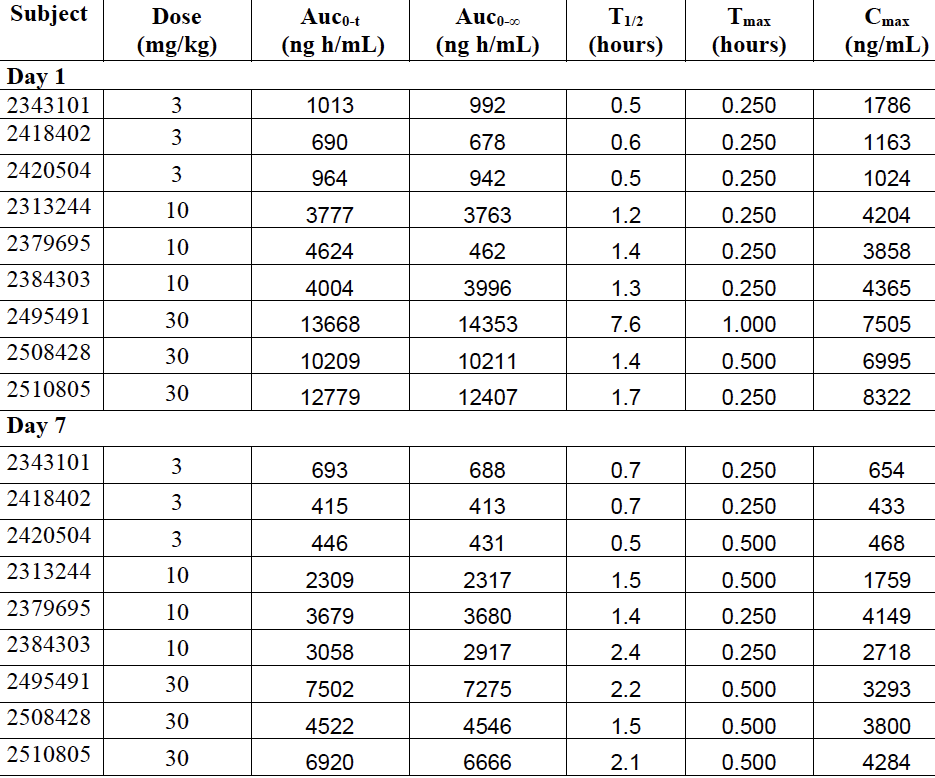


**PK by Subject** (male **Beagle dog**) receiving **SRP-001** **oral nanosuspension dose** up to **7days.**


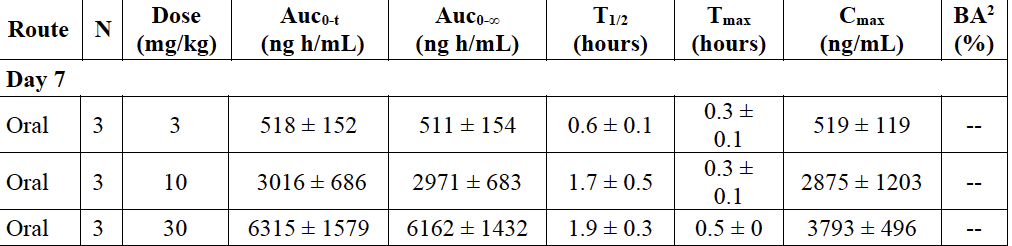

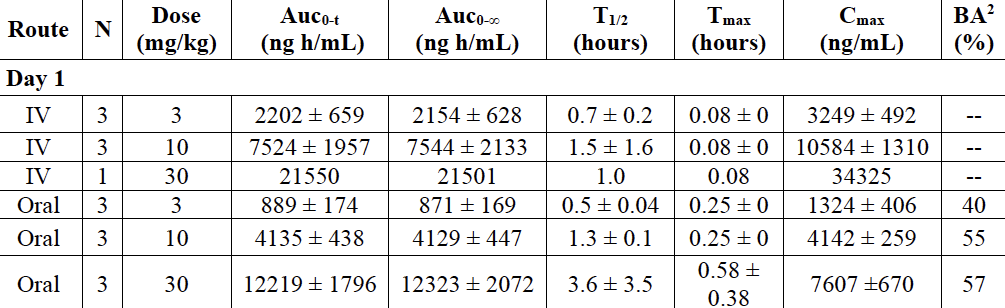


**Dog PK Parameters** receiving **oral** or **IV SRP-001 nanosuspension dose.**
